# Minocycline for acute basilar artery occlusion stroke undergoing endovascular treatment: a randomized, evaluator-blinded, open-label, pilot trial

**DOI:** 10.1186/s12916-026-05092-4

**Published:** 2026-07-27

**Authors:** Zhirong Fan, Jingjing Zhao, Rui Shi, Li Yao, Huiping Zhang, Rong Yin, Weiwang Li, Liping Yu, Xiangjun Yuan, Yi Jia, Bo Song, Baiya Fan, Xingshun Ma, Xue Li, Deshuai Li, Xiao Zhang, Yang Li, Ling Wang, Duolao Wang, Dong Wei, Wen Jiang, on the behalf of the MIST-B Study Group

**Affiliations:** 1 Department of Neurology, Xijing Hospital, Fourth Military Medical University, Xi’an, China; 2 Department of Neurology, Xi’an XD Group Hospital, Xi’an, China; 3 Department of Neurology, Baoji High-tech Hospital, Baoji, China; 4 Department of Neurology, Gansu Provincial Maternity and Child-care Hospital, Gansu Provincial Central Hospital), Lanzhou, China; 5 Department of Neurology, Xi’an Daxing Hospital, Xi’an, China; 6 Department of Neurology, The First People’s Hospital of Xianyang, Xianyang, China; 7 Department of Neurology, Weinan Central Hospital, Weinan, China; 8 Department of Neurology, Xi’an Gaoxin Hospital, Xi’an, China; 9 Department of Neurosurgery, Xianyang Hospital of Yan’an University, Xianyang, China; 10 Department of Neurology, Yan’an People’s Hospital, Yan’an, China; 11 Department of Neurology, The First Hospital of Yulin, Yulin, China; 12 Department of Neurology, Yulin Hospital, The First Affiliated Hospital of Xi’an Jiaotong University, Yulin, China; 13 Department of Health Statistics, Fourth Military Medical University, Xi’an, China; 14 Department of Clinical Sciences, Liverpool School of Tropical Medicine, Liverpool, UK

**Keywords:** Minocycline, Acute ischemic stroke, Endovascular treatment, Basilar artery occlusion

## Abstract

**Background:**

Minocycline is a promising anti-inflammatory agent for reducing futile recanalization, but whether it improves clinical outcomes in basilar-artery stroke patients treated with endovascular therapy (EVT) remains unclear. In this study, we aimed to assess the efficacy and safety of oral minocycline combined with EVT on outcomes in patients who had basilar-artery stroke.

**Methods:**

The Minocycline for acute Ischemic Stroke undergoing endovascular Treatment due to Basilar artery occlusion (MIST-B) trial was a multicenter, randomized, evaluator-blinded, open-label, pilot trial in 12 Chinese hospitals. Adults with basilar-artery stroke no more than 24 h after onset were randomized (1:1) to receive oral minocycline (200 mg loading, then 100 mg every 12 h for 5 days) plus EVT or EVT alone. The primary efficacy outcome was the expanded National Institutes of Health Stroke Scale (e-NIHSS) score at day 5; the primary safety outcome was symptomatic intracranial hemorrhage (sICH) within 24 h. Participants were followed up 90 days after randomization. Modified intention-to-treat analysis examined treatment effect for the primary efficacy outcome using linear mixed models, with the baseline measurement of e-NIHSS score as a covariate.

**Results:**

From March 4, 2023, to March 4, 2025, 90 patients were randomized, and 89 were analyzed (mean age 61.7 years, 31.5% women). At day 5, median e-NIHSS was 12.0 (IQR 3.5 to 30.0) in the minocycline group and 10.5 (IQR 2.0 to 35.0) in the control group; corresponding mean values were 15.0 (SD 13.5) and 17.2 (SD 16.9), respectively (adjusted mean difference − 2.83 [95% CI -7.86 to 2.20]; *P* = 0.27). sICH within 24 h occurred in 2/37 (5.4%) of participants in the minocycline group vs. 6/41 (14.6%) in the control group (adjusted risk ratio, 0.70; 95% CI, 0.11 to 4.67). Other secondary efficacy and safety outcomes, including 90-day modified Rankin Scale, mortality, and adverse events, showed no significant differences between groups.

**Conclusions:**

Minocycline was well-tolerated in this population; however, these results did not provide evidence to support prescribing minocycline to improve early neurological outcomes. Larger trials of minocycline with extended treatment duration are warranted.

**Registration:**

The trial was registered at ClinicalTrials.gov (NCT05512910) on August 23, 2022.

**Supplementary Information:**

The online version contains supplementary material available at https://doi.org/10.1186/s12916-026-05092-4.

## Background

Basilar artery occlusion (BAO) is a catastrophic subtype of ischemic stroke and accounts for approximately 1% of all ischemic strokes and 5% to 10% of large-vessel intracranial occlusions. It is associated with severe disability or death in nearly 70% of cases [1]. Although recent randomized clinical trials have shown that endovascular therapy (EVT) is superior to medical management alone for acute BAO [2, 3], fewer than half of patients who undergo successful recanalization achieve functional independence [4]. This persistent gap underscores the need for adjunctive neuroprotective strategies to enhance outcomes following reperfusion.

Several agents, including methylprednisolone and edaravone dexborneol, have been reassessed in the context of reperfusion therapy, but clinical trials have yielded negative results [5, 6]. These findings reflect a broader translational challenge, in which therapies with strong preclinical efficacy fail to demonstrate clinical benefit. Minocycline, a highly lipophilic tetracycline derivative, readily crosses the blood-brain barrier (BBB) and exerts pleiotropic neuroprotective actions [7, 8]. It inhibits microglial activation [9], reduces T-cell migration [8], suppresses pro-inflammatory signaling [8], attenuates neuronal apoptosis [10], decreases oxidative stress [11], and downregulates matrix metalloproteinases-9 [12], thereby stabilizing the neurovascular unit and limiting BBB disruption and hemorrhagic transformation [13]. In experimental stroke models, minocycline consistently improved behavioral recovery, reduced infarct volume, and mitigated reperfusion injury [9, 14]. Despite these promising findings, clinical evidence for minocycline’s efficacy in ischemic stroke remains inconclusive. Most prior trials were conducted in the pre-EVT era and were limited by small sample sizes [15–17]. More recently, the EMPHASIS trial reported improved clinical outcomes with minocycline, supporting further investigation in the modern reperfusion era, where EVT may enhance drug delivery to ischemic brain tissue and thereby potentiate its therapeutic effects [18, 19].

In this study, we conducted a prospective, randomized, multicenter, open-label, pilot trial with blinded outcome assessment, to evaluate the efficacy and safety of oral minocycline in patients with acute basilar-artery stroke undergoing EVT.

## Methods

### Trial design and oversight

The Minocycline for acute Ischemic Stroke undergoing endovascular Treatment due to Basilar artery occlusion (MIST-B) study was a multicenter, prospective, randomized, controlled, open-label, evaluator-blinded, pilot trial, conducted at 12 comprehensive stroke centers in China. Patients were enrolled between March 4, 2023 and March 4, 2025, with final follow-up on June 5, 2025.

The trial was designed and conducted by a steering committee composed of independent academic investigators and monitored by an independent data and safety management board (DSMB). The trial was conducted according to the recommendation of Good Clinical Practice and principles of the Declaration of Helsinki Harmonization.

### Standard protocol approvals, registrations, and patient consents

The study design and protocol were approved by the Ethics Committee at Xijing Hospital, Xi’an, China (XJLL-KY2022184). The study protocol and statistical analysis plan are available in Additional file 1 and Additional file 2, respectively. Written informed consents were obtained before study enrollment from participants or their legally authorized representatives. On November 22, 2022, before enrollment, the steering committee, in agreement with the DSMB, revised the study design, inclusion criteria, and primary outcome (see Additional file 1). This trial was registered at ClinicalTrials.gov, NCT05512910.

### Participants

Patients who planned to undergo EVT were eligible for inclusion if they were adults aged 18 years or older, had AIS due to occlusion (modified Thrombolysis in Cerebral Infarction score 0–1) in basilar artery or intracranial segments of vertebral arteries (VAs) determined by computed tomographic angiography (CTA) or magnetic resonance angiography (MRA), had a baseline expanded National Institutes of Health Stroke Scale (e-NIHSS) of at least 6, had a pre-stroke modified Rankin Scale (mRS) no more than 1, and presented within 24 h of symptom onset or since they were last known well. Neuroimaging inclusion criteria included posterior circulation Acute Stroke Prognosis Early CT score (pc-ASPECTS) ≥ 5 and Pons-midbrain-index (PMI) of < 3. Patients were excluded in three categories: (1) Neuroimaging exclusion criteria at baseline, including intracranial hemorrhage and complete cerebellar infarct with significant mass effect; (2) Pre-existing illness that interferes with neurological evaluation or prognostic, including stroke within 90 days, cardiopulmonary resuscitation within 10 days, uncontrolled diabetes or hypertension, known hereditary or acquired hemorrhagic diathesis, acute or chronic renal failure, or mental illness; (3) Other conditions that interfere with the study, including known allergy or hypersensitivity of investigational drugs, pregnancy or breastfeeding, participated in other clinical studies, unlikely to be available for follow-up at day 90 due to illness (except for stroke) or compliance. Detailed inclusion and exclusion criteria are provided in Table S1.

### Randomization and masking

Patients were randomized in a 1:1 ratio to the minocycline group (oral minocycline combined with EVT) or the control group (EVT only) through a web-based randomization procedure (http://iwrs.xarsk.com/). Researchers had access to the randomization results except the independent assessor at each trial site and statisticians. The randomization sequence was generated by an independent statistician using SAS V.9.4 software with a block size of 4. Randomization was not stratified by participating center or other parameters. Treatment allocation was unblinded to the patients or their legal representatives, and researchers, excluding those who evaluated imaging, assessed outcome, and conducted analysis.

### Interventions and procedures

Patients assigned to minocycline group were prescribed minocycline capsules (Hisun Pharmaceutical Group Co., Ltd., Hangzhou, China) as soon as possible after randomization, ideally before the EVT or no more than 1 h after EVT. Previous pharmacokinetic study demonstrated that a 200 mg oral loading dose of minocycline achieves therapeutic drug concentrations within 2–3 h [20]. In addition, inflammatory responses related to ischemic and reperfusion are most active during the first several days after stroke onset [21]. Therefore, we selected a regimen of 200 mg orally followed by 100 mg every 12 h for 5 consecutive days after enrollment, consistent with previous stroke studies of minocycline [22]. Patients who were considered at risk for aspiration or unable to swallow were administered via a feeding tube. Both groups in the trial underwent rapid EVT using available devices. The choice of EVT technique was left to the discretion of the treating physician. Intravenous/intraarterial thrombolysis before or after endovascular recanalization was allowed and recorded in details. Both groups received standard medical treatment and postoperative care that were guided by the stroke national practice guidelines international [23].

### Data collection

At the time of baseline screening, demographics, medical history (hypertension, atrial fibrillation, diabetes, cerebral stroke, smoking, alcohol use), results of physical examinations, vital signs, laboratory tests, clinical scores (NIHSS, e-NIHSS, modified Barthel Index[mBI], mRS), and head image results were recorded. Visits were scheduled for 24 h, 5 days, 30 days and 90 days after randomization (Table S2). During the hospitalization, concomitant treatments, ICU/hospital length of stay, mechanical ventilation, and inflammatory markers were documented. At all visits, the neurological deficit (e-NIHSS and NIHSS), long-term functional outcome (mBI and mRS), adverse events (AEs), and serious AEs (SAEs) were assessed. For patients discharged prior to 5-day visit, clinical assessments at discharge were recorded. If regularly face-to-face visits were unavailable, telephone follow-up about the mRS and mBI were performed. Head CT scans were repeated at 24 h or following neurological deterioration with increase of NIHSS ≥ 4, and head MRI was performed at 5 days.

### Outcomes

The primary efficacy outcome was e-NIHSS score at day 5. The secondary efficacy outcomes included NIHSS and e-NIHSS at 24 h after randomization; NIHSS, ICU/hospital length of stay, rate of mechanical ventilation, incidence of pneumonia, leukocyte count, and neutrophil percentage/absolute value at day 5; mBI and mRS at day 30 and day 90. All scales outcomes were assessed by the independent assessor in each center during a face-to-face visit or a structured telephone interview.

The primary safety outcome was symptomatic intracranial hemorrhage (sICH) at 24 h after randomization. Other safety measures included sICH at day 3, mortality due to any cause, AEs and SAEs at day 90.

### Statistical analysis

Due to the lack of prior application of e-NIHSS scores in RCTs, this trial used the NIHSS parameters in previous studies to estimate the sample size. The sample-size calculation was based on the ATTENTION trial, with an expected NIHSS scores of 16 ± 5.3 in patients with thrombectomy at 5–7 days or discharge calculated by the methods of Iztok Hozo [2, 24]. Given the lack of previous data on oral minocycline to EVT, it is considered a reasonable prediction that NIHSS scores in the minocycline group would be 3 points lower than those in the control group at 5 days after randomization, based on the discussion by the Academic Committee and the Executive Committee. Therefore, we calculated that a sample size of 90 patients (45 patients in each group with a dropout rate of 10%) would detect a 3 points difference (16 ± 5.3 for the control group and 13 ± 5.3 for the intervention group) in e-NIHSS scores of patients at day 5, with a power of 80% and two-sided significance levels of 0.05 for the analysis of the primary effectiveness outcome.

The primary analysis of both primary and secondary efficacy outcomes was conducted in the modified intention-to-treat (mITT) population, defined as those according to their randomization assignment with a valid assessment of e-NIHSS score at 5 days. The analysis of efficacy outcomes for per-protocol (PP) population was repeated to assess the robustness of our conclusion. Analyses of AEs were based on the safety population, which consisted of all randomized participants who had at least one follow-up safety assessment.

The treatment effect for the primary efficacy outcome was measured by linear mixed models (mean difference) with 95% CIs. The mixed model included treatment (intervention vs. control), time (24 h, 5 days), interaction between treatment and time as fixed effects, baseline measurement as a covariate, and subject as random effect. Safety and secondary efficacy analyses with continuous outcomes were analyzed using linear regression. The win ratio method was used if normality assumption was seriously violated. Binary outcomes were analyzed using modified Poisson regression models. Ordinal outcomes were analyzed using generalized odds ratio (win ratio). The treatment effects were reported as the risk ratio, mean difference or win ratio as appropriate, along with the 95% CIs. Normality was assessed using Q-Q plot. The effects of minocycline were adjusted for five prespecified covariates (age, sex, time from onset to random assignment, PMI index and baseline e-NIHSS score) and one additional post-hoc covariate (intravenous thrombolysis). The covariates adjusted risk ratio, mean difference and win ratio were calculated using inverse probability of treatment weighting method. The treatment effect on the primary efficacy outcome was further assessed in subgroups as follows: age, sex, time from onset to recanalization, PMI index, baseline pc-ASPECTS score, baseline e-NIHSS score, occlusion location, TOAST etiology, and intravenous thrombolysis. Post-hoc subgroup analysis was performed to explore the consistency of the treatment effect across subgroup defined by baseline NIHSS (≥ 10 vs. <10). In addition, an exploratory post-hoc analysis within the minocycline group compared outcomes according to the timing of minocycline administration (before arterial puncture vs. within 1 h after EVT).

For the primary outcome, a 2-sided *P* value of less than 0.05 was considered statistically significant. All statistical analyses will be performed using Python version 3.12 and R version 4.3.1.

## Results

### Characteristics of the patients

**Fig. 1:**
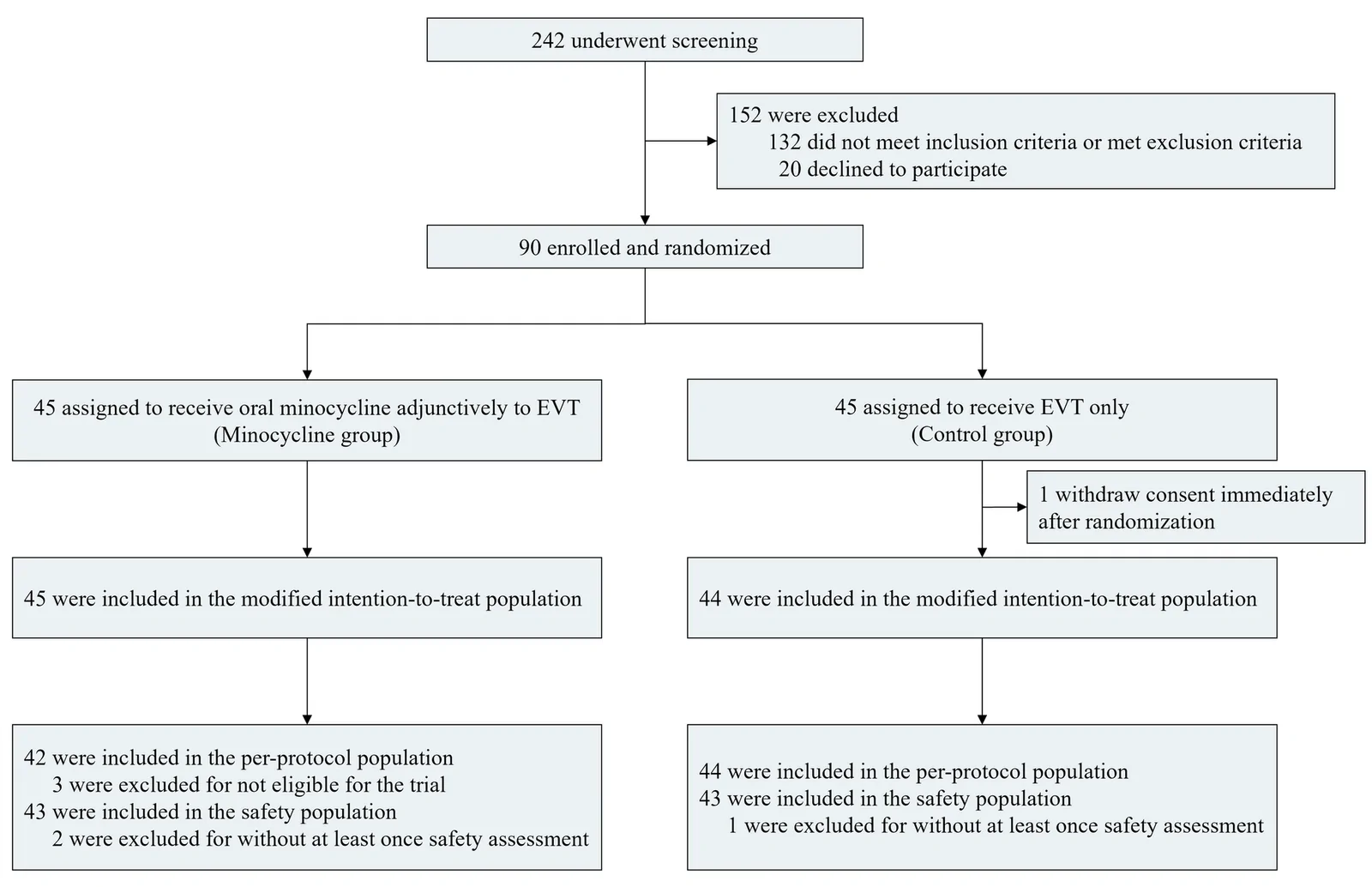
CONSORT diagram. EVT indicates endovascular treatment

Between March 4, 2023, and March 4, 2025, 242 patients with AIS undergoing EVT due to occlusion in basilar artery or intracranial segments of vertebral arteries were screened, of whom 90 patients underwent randomization at 12 centers in China (Fig. S1 and Table S3). After excluding 1 patient who withdrew consent immediately after randomization, 89 patients (mean [SD] age, 61.7 [12.3] years; 28 females [31.5%]) were included in the final mITT analysis, consisting of 45 patients in the minocycline group and 44 patients in the control group (Fig. 1). Three patients in the minocycline group, who were not eligible for the MIST-B trial, could not be included in the per-protocol analyses set. Demographic and clinical characteristics were similar between two groups in both mITT analyses and per-protocol analyses **(**Table 1, Table S4, and Table S5).

**Table 1:** Baseline characteristics of study participants (modified intention-to-treat population)

Characteristics	Minocycline group (*n* = 45)	Control group (*n* = 44)
Age, mean (SD), yr	62.0 (12.5)	61.4 (12.1)
Female, n/N (%)	14/45 (31.1)	14/44 (31.8)
Body-mass index, kg/m^2^	24.4 (22.9, 25.4)	23.5 (22.2, 25.7)
Medical history ^a^, n/N (%)		
Hypertension	32/45 (71.1)	30/44 (68.2)
Atrial fibrillation	4/45 (8.9)	4/44 (9.1)
Diabetes	11/45 (24.4)	11/44 (25)
Cerebral stroke	3/45 (6.7)	7/44 (15.9)
Smoking	16/45 (35.6)	16/44 (36.4)
Alcohol use	9/45 (20.0)	8/44 (18.2)
Pre-stroke mRS score, median (IQR)	0 (0, 0)	0 (0, 0)
Disease severity		
NIHSS score, median (IQR)	18.0 (10.0, 35.0)	19.5 (10.5, 35.0)
e-NIHSS score, median (IQR)	20 (12.0, 35.0)	20.5 (11.0, 35.0)
pc-ASPECTS ^b^, median (IQR)	7.0 (6.0, 9.0)	8.0 (6.0, 8.0)
PMI index ^b^, median (IQR)	1.0 (0.0, 2.0)	1.0 (0.0, 2.0)
TOAST etiology, n/N (%)		
Cardioembolic	8/45 (17.8)	7/44 (15.9)
Large artery atherosclerosis	34/45 (75.6)	35/44 (79.5)
Others	0/42 (0.0)	1/44 (2.3)
Unknown	3/45 (6.7)	1/44 (2.3)
Occlusion site based on baseline image ^c^, n/N (%)		
V4 segments of vertebral arteries	17 (37.8)	17 (38.6)
Basilar artery	27 (60.0)	27 (61.4)
Intravenous thrombolysis, n/N (%)	10/45 (22.2)	15/44 (34.1)
Time from stroke onset, median (IQR), mins		
To randomization	449 (326, 903)	344 (163, 684)
To puncture	480 (360, 955)	386 (185, 727)
To recanalization ^d^	609 (434, 1078)	476 (275, 827)
Blood glucose, median (IQR), mmol/L	8.3 (6.9, 10.0)	8.2 (6.5, 10.3)
SBP, median (IQR), mm Hg	148 (132, 165)	140 (131, 152)
DBP, median (IQR), mm Hg	85 (78, 97)	82 (76, 90)

Abbreviations: *CTA* computed tomography angiography, *DBP* diastolic blood pressure, *e-NIHSS* expanded National Institutes of Health Stroke Scale, *mRS* modified Rankin Scale, *MRA* magnetic resonance angiography, *NIHSS* National Institutes of Health Stroke Scale, *pc-ASPECTS* posterior circulation Alberta Stroke Program Early Computed Tomography Score, *PMI* Pons-midbrain-index, *SBP* systolic blood pressure, *TOAST* Trial of Org 10,172 in Acute Stroke Treatment

^a^ Comorbidities based on family or patient report

^b^ Values of pc-ASPECTS and PMI were determined by diffusion-weighted imaging or non-contrast cranial computed tomography at baseline assessment. On the basis of assessment, 1 patient in the minocycline group were excluded from the analyses due to only 1 point of pc-ASPECTS

^c^ Values of occlusion location were determined by CTA or MRA at baseline assessment. Images were lost or unable to complete CTA/MRA examination for 5 patients in the minocycline group and for 2 in the control group. For these 7 patients, the occlusion of V4 segments of vertebral arteries or basilar artery were confirmed by digital subtraction angiography. On the basis of assessment, 1 patient in the minocycline group were excluded from the analyses due to no occlusion of basilar artery or V4 segments of vertebral arteries

^d^ Recanalization was defined as the first visualization of successful reperfusion, as indicated by a modified Thrombolysis in Cerebral Infarction (mTICI) grade of 2b or 3. Data on the time from stroke onset to recanalization were missing for 1 patient in the minocycline group and 4 in the control group, due to unsuccessful recanalization

### Intervention

Predominant occlusion locations on baseline imaging were the V4 segments of vertebral arteries (34 in 89, 38.2%) and basilar artery (54 in 89, 60.7%) (Table 1). We observed a discrepancy between CTA/MRA and digital subtraction angiography (DSA) ratings of the occlusion location, with predominant V1-V3 segments of vertebral arteries for 3 patients in the minocycline group and for 1 in the control group (Table S4). All participants in mITT population received EVT, and 94.4% had a final mTICI score of 2b or 3 (indicating successful reperfusion). The median interval between hospital admission and arterial puncture was 110 (69–181) mins in the minocycline group and 101 (70–142) mins in the control group. Most patients received minocycline capsules before arterial puncture (38 in 45, 84.4%), while only 7 patients (15.6%) received minocycline within 1 h after EVT (Table S4).

### Primary efficacy outcome

**Fig. 2:**
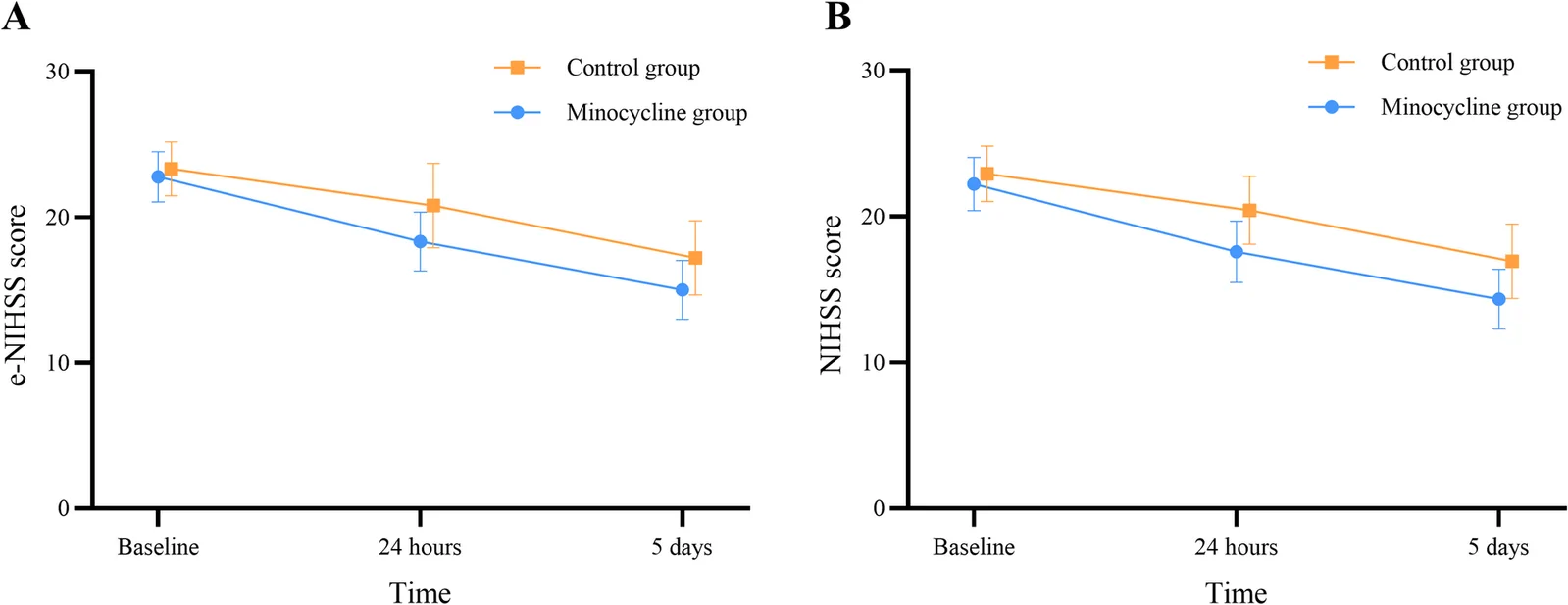
The mean score (± SE) of e-NIHSS (**A**) and NIHSS (**B**) during the in-hospital period (modified intention-to-treat population). For patients who died within the study period (1 patients in the control group at 24 h, and 4 patients in the control group at 5 days), the scores were assigned as 42. NIHSS, National Institutes of Health Stroke Scale; e-NIHSS, expanded National Institutes of Health Stroke Scale

**Fig. 3:**
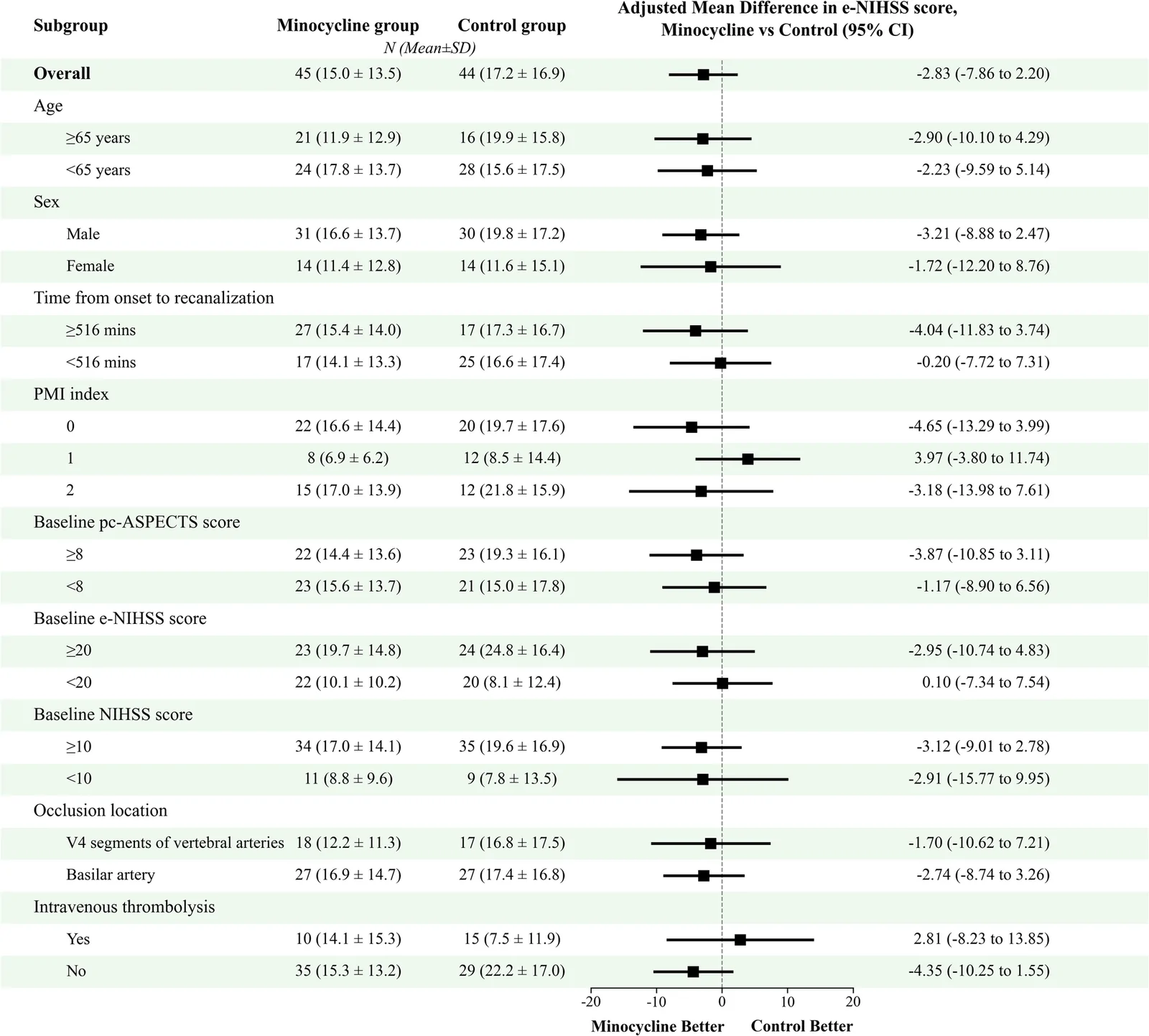
Subgroups analyses of e-NIHSS scores at 5 days (modified intention-to-treat population). The treatment effect was assessed in prespecified subgroups (age, sex, time from onset to recanalization, PMI index, baseline pc-ASPECTS score, baseline e-NIHSS score, occlusion location, TOAST etiology, and intravenous thrombolysis) and post-hoc subgroup (baseline NIHSS score). The widths of confidence intervals have not been adjusted for multiple comparisons, and no conclusions can be drawn from these results. The time from stroke onset to recanalization and baseline e-NIHSS score were divided at median of the whole population as prespecified in the statistical analysis plan. Only two common types of TOAST etiology, cardioembolic and large artery atherosclerosis, were retained in subgroup analyses. The arrow indicates that the 95% CI was beyond the scale. PMI, Pons-midbrain-index; pc-ASPECTS, posterior circulation Alberta Stroke Program Early Computed Tomography Score; NIHSS, National Institutes of Health Stroke Scale; e-NIHSS, expanded National Institutes of Health Stroke Scale; TOAST, Trial of Org 10,172 in Acute Stroke Treatment

The baseline e-NIHSS score was comparable in the minocycline group and the control group (Table 1). There was no significant difference in e-NIHSS score at day 5 between the minocycline group (15.0 ± 13.5) and the control group (17.2 ± 16.9), yielding an unadjusted mean difference of -1.84 (95% CI, -7.04 to 3.36; *P* = 0.51) and an adjusted mean difference of -2.83 (95% CI, -7.86 to 2.20; *P* = 0.27) (Table 2). The changes of NIHSS score and e-NIHSS score in the first 5 days after enrollment are shown in Fig. 2. In terms of per-protocol analyses and subgroup analyses, the treatment effect of minocycline also did not show superiority in the e-NIHSS score at day 5 (Table S6 and Fig. 3). In post-hoc subgroup analysis stratified by baseline NIHSS (≥ 10 vs. <10), no significant between-group differences in 5-day e-NIHSS scores were observed (Fig. 3). In the minocycline group, an exploratory post-hoc analysis showed no significant differences in 5-day e-NIHSS scores between patients who received minocycline before arterial puncture (*n* = 38, mean 15.5 [SD 13.7]) and those treated within 1 h after EVT (*n* = 7, mean 12.4 [SD 12.8]) (*P* = 0.70).

**Table 2:** Primary and secondary outcomes by treatment (modified intention-to-treat population)

Outcome	Minocycline group (*n* = 45)	Control group (*n* = 44)	Measure of effect	Unadjusted value (95% CI) ^a^	Adjusted value (95% CI) ^b^
Efficacy outcomes					
e-NIHSS score at 5 d (primary outcome), no, Mean (SD) ^c^	45, 15.0 (13.5)	44, 17.2 (16.9)	Mean difference	-1.84 (-7.04, 3.36)	-2.83 (-7.86, 2.20)
e-NIHSS score at 24 h, no, Mean (SD) ^c^	45, 18.3 (13.7)	44, 20.8 (15.2)	Mean difference	-2.12 (-7.32, 3.08)	-3.11 (-8.14, 1.92)
mRS score at 90 d, No. of wins/total No. of pairs ^d^	881/1935 (45.5%)	680/1935 (35.1%)	Win ratio	1.30 (0.72, 2.34)	1.36 (0.75, 2.45)
mRS score of 0–1 at 90 d, n/N (%) ^e^	19/45 (42.2)	16/43 (36.4)	Risk ratio	1.13 (0.68, 1.90)	1.24 (0.75, 2.05)
mRS score of 0–2 at 90 d, n/N (%) ^e^	23/45 (51.1)	20/43 (45.5)	Risk ratio	1.10 (0.72, 1.69)	1.23 (0.83, 1.82)
mRS score of 0–3 at 90 d, n/N (%) ^e^	26/45 (57.8)	23/43 (52.7)	Risk ratio	1.08 (0.74, 1.57)	1.21 (0.86, 1.70)
NIHSS score at 24 h, no, Mean (SD) ^c^	45, 17.6 (14.1)	44, 20.4 (15.4)	Mean difference	-2.37 (-7.64, 2.90)	-3.40 (-8.52, 1.71)
NIHSS score at 5 d, no, Mean (SD) ^c^	45, 14.3 (13.7)	44, 16.9 (16.9)	Mean difference	-2.14 (-7.41, 3.13)	-3.17 (-8.29, 1.95)
mBI scores at 90 d, No. of wins/total No. of pairs ^d^	768/1935 (39.7%)	616/1935 (31.8%)	Win ratio	1.25 (0.64, 2.38)	1.29 (0.66, 2.53)
Safety outcome					
sICH at 24 h, n/N (%) ^e f^	2/37 (5.4)	6/41 (14.6)	Risk ratio	0.37 (0.08, 1.72)	0.70 (0.11, 4.67)
sICH at 3 d, n/N (%) ^e f^	3/41 (7.3%)	6/41 (14.6%)	Risk ratio	0.50 (0.13, 1.87)	0.73 (0.18, 2.95)
Mortality at 90 d, n/N (%) ^e^	10/43 (23.3%)	14/42 (33.3%)	Risk ratio	0.70 (0.35, 1.39)	0.72 (0.38, 1.39)
AE at 90 d, n/N (%) ^e^	28/43 (65.1%)	35/43 (81.4%)	Risk ratio	0.80 (0.62, 1.04)	0.78 (0.61, 1.00)
SAE at 90 d, n/N (%) ^e^	16/43 (37.2%)	18/43 (41.9%)	Risk ratio	0.89 (0.53, 1.50)	0.88 (0.54, 1.43)

Abbreviations: *AE* adverse event, *e-NIHSS* expanded National Institutes of Health Stroke Scale, *mRS* modified Rankin Scale, *mBI* modified Barthel Index score, *NIHSS* National Institutes of Health Stroke Scale, *sICH* symptomatic Intracranial Hemorrhage, *SAE* severe adverse event

^a^ Analyses of efficacy outcomes and safety outcomes were conducted in modified intention-to-treat population and in safety population, respectively

^b^ Covariates adjusted included age, sex, time from onset to random assignment, PMI index, baseline e-NIHSS score and intravenous thrombolysis. If a model with covariates added did not converge, IPTW approach was used

^c^ The linear mixed model was used, which included treatment, time, interaction between treatment and time as fixed effects, baseline measurement as a covariate, and subject as random effect, from which treatment difference with 95% CI at each time was calculated. Normality was assessed using Q-Q plot

^d^ No. of wins/total No. of pairs (%), win ratio (generalized odds ratio) were calculated using WINS package

^e^ Unadjusted and adjusted risk ratio were calculated using modified Poisson regression model

^f^ Patients unable to undergo the scheduled CT because of clinical instability or death after EVT were excluded from the analysis (24 h: 6 vs. 2; 3 days: 2 vs. 2, minocycline vs. control)

### Secondary efficacy outcomes

There was no significant difference in secondary efficacy outcomes between the two groups (Table 2 and Table S7). The median 90-day mRS score was 2 (0–5) in the minocycline group and 3 (1–6) in the control group (adjusted win ratio, 1.36; 95% CI, 0.75 to 2.45) (Fig. S2). Other secondary outcomes, including early neurological deficit (e-NIHSS at 24 h, and NIHSS at 24 h and day 5), clinical events (rates of mechanical ventilation and pneumonia), laboratory values (day 5 leukocyte count and neutrophil parameters), length of stay (ICU and hospital), and functional independence (mBI and dichotomized mRS at 90 days), were all comparable between the two groups. (Table 2 and Table S7). In terms of per-protocol analyses, the treatment effect of minocycline also did not show superiority in secondary efficacy outcomes (Table S6).

### Safety outcomes

The primary safety outcome (sICH at 24 h) was observed in 2 of 37 patients (5.4%) in the minocycline group and 6 of 41 patients (14.6%) in the control group (adjusted risk ratio, 0.70 [95% CI, 0.11 to 4.67; *P* = 0.72]. The rate of any AEs or SAEs, and other secondary safety outcomes did not differ between the two groups (Table 2). Over the 90-day follow-up period, the most common SAEs were mortality (minocycline group 10 [23.3%] vs. control group 14 [33.3%]) and pneumonia with respiratory failure (minocycline group 12 [27.9%] vs. control group 16 [37.2%]), which did not differ between groups. Details of AEs and SAEs are shown in Table S8, Table S9, and Table S10.

## Discussion

In this multicenter, randomized pilot trial, oral minocycline did not significantly improve early neurological outcomes in patients with basilar artery occlusion undergoing endovascular therapy. Nevertheless, this trial provided high-quality randomized evidence and robust safety data in a population with limited treatment options. These findings highlight the difficulty of translating neuroprotective strategies into effective therapies in the reperfusion era and emphasize the need for better-designed future trials rather than routine clinical use.

Futile recanalization (FR) continues to affect approximately 50% of patients undergoing EVT and likely results from a combination of baseline factors and post-reperfusion mechanisms. These include infarct core size and the eloquence of the affected brain regions, as well as processes such as microvascular injury (e.g., no-reflow and hemorrhagic transformation), early arterial re-occlusion, inadequate collateral circulation, and impaired cerebrovascular autoregulation [25]. Given the potential role of post-reperfusion inflammation in these processes, anti-inflammatory therapies have been explored as adjunctive strategies for ischemic stroke. Preclinical models have indicated potential benefits of agents such as methylprednisolone, fingolimod, natalizumab, and cyclosporine in stroke, their efficacy remains unconfirmed in clinical trials [26–29]. Most of the prior trials investigating anti-inflammatory agents were conducted before the advent of EVT as a viable treatment option. Recent EVT-era trials, such as MAVEL and INSIST-ED, similarly failed to prove benefits of adjunctive neuroprotective strategies on long-term functional outcomes [5, 6].

Minocycline differs in targeting multiple pathways relevant to ischemia, including reperfusion injury, microglial activation, oxidative stress, apoptosis, and blood brain barrier disruption, and consistently reduced infarct size and reperfusion injury in experimental models [15, 22]. The MIST-B trial aimed to evaluate the efficacy of oral minocycline in BAO patients undergoing EVT. Successful reperfusion following EVT may enhance the delivery of minocycline to the ischemic territory, thereby potentiating its neuroprotective effects [30]. However, in this pilot trial, there was no statistically significant difference in the primary outcome between groups, although numerical differences in several efficacy and safety outcomes favored the minocycline group. These numerical trends may be attributable to higher baseline NIHSS scores and lower successful recanalization rates in control group. In addition, the relatively high rate of symptomatic intracranial hemorrhage in the control group may also have influenced outcome measures, possibly due to random variation, center-level heterogeneity, or differences in thrombectomy strategies or intravenous thrombolysis. Nevertheless, this pilot study contributes to the evolving clinical evidence on minocycline in acute ischemic stroke and complements the recently published EMPHASIS trial, which suggested clinical benefits in patients (NIHSS score 4–25) irrespective of reperfusion therapy and raised the hypothesis that its effects may be greater in patients undergoing reperfusion [19].

Several factors may explain the discrepancy between the robust benefits observed in animal models and the outcomes of our clinical trial. Firstly, the pathophysiological context, immune regulation, and pharmacokinetic profiles differ substantially between rodents and humans, which may limit the translational validity of preclinical findings. Moreover, the inflammation responses after ischemic injury are complex and bifacial; part of the inflammatory cascades is involved in increased damage and edema formation whereas other inflammatory pathways are important for neuroprotection, regeneration and restoration [31]. Increasing evidence suggests that these post-ischemic responses are further regulated by heterogeneous and temporally dynamic cell-specific molecular programs [32, 33]. In addition, functional recovery after reperfusion may depend not only on focal ischemic injury, but also on broader structural reserve and adaptive network-level responses, such as dynamic interhemispheric interactions [34]. Such biological complexity may partly explain why single-target neuroprotective strategies frequently demonstrate promising preclinical efficacy but inconsistent clinical translation in heterogeneous stroke populations. Secondly, high-volume center expertise (≥ 50 EVTs/year) achieved superior reperfusion rates (mTICI 2b-3: 94.2%) compared to those reported in the BAOCHE and ATTENTION trials (84–88%), which was associated with higher functional independence rates of 54.7% vs. 46% in BAOCHE and ATTENTION trials [2, 3]. Reperfusion is an effective treatment for improving the prognosis of stroke, while anti-inflammatory therapy targets the inflammatory changes that occur after reperfusion. When the efficacy of stroke reperfusion is high, the improvement in prognosis offered by medication may be overshadowed by the direct effects of reperfusion.

Beyond these biological considerations, several methodological aspects of the trial also warrant consideration. Firstly, the 5-day treatment regimen was selected to target the early post-reperfusion inflammatory phase and to maintain consistency with previous stroke studies of minocycline [22]. However, the microglial activation remains significant after 2 weeks of cerebral ischemia, which indicated the effect of minocycline might be diminished due to the short duration of drug administration. A recent study with cell and animal middle cerebral artery occlusion models showed that extended minocycline treatment duration (14 days vs. 5 days) significantly improved neuroprotective effects by anti-inflammation [35]. Whether alternative dosing strategies, including longer treatment duration, might improve efficacy remains unclear and warrants further investigation. Secondly, given the exploratory nature of adjunctive minocycline therapy, we selected the 5-day e-NIHSS score as a surrogate endpoint for the 90-day mRS. This choice was based on evidence that early NIHSS scores at 5–7 days show moderate-to-good agreement with 90-day outcomes and may improve trial feasibility [36]. Moreover, the e-NIHSS score may provide greater sensitivity for detecting neurological changes in BAO patients [37]. However, the observed variability in e-NIHSS scores (SD = 15.2) was greater than anticipated (SD = 5.3 based on NIHSS data from the ATTENTION trial) [2], reducing the statistical power to detect a treatment effect and increasing the possibility of a Type II error. Although the e-NIHSS has been adopted in several observational studies [37, 38], it has not been validated as a standard efficacy endpoint in randomized trials. In addition, early neurological improvement scales may not fully capture the complex and multidimensional process of post-stroke recovery. Therefore, to address these limitations and prevent Type II errors, larger trials using the commonly employed 90-day mRS as the primary outcome and incorporating an adequate sample size are warranted.

### Limitations

There are several limitations in this trial. Firstly, the limited sample size determined the exploratory nature of this study, so the null findings should be interpreted cautiously and considered exploratory. Secondly, the open-label design without placebo may have introduced bias related to concurrent rehabilitation, supportive care, and patient reporting, potentially influencing secondary functional outcomes such as the mRS and mBI. To minimize this risk, the clinical treatment team was not involved in the study, and clinical outcomes were assessed by independent assessors who were blinded to treatment allocation. Thirdly, the efficacy of minocycline could be limited by the short duration of drug administration. Therefore, a longer treatment duration (≥ 14 days) is needed in further research to confirm the therapeutic effect of minocycline. Fourthly, our trial lacks biomarkers such as matrix metalloproteinase-9 or neutrophil-to-lymphocyte ratio that might help identify patients most likely to benefit from anti-inflammatory therapy. Thus, future studies should incorporate such biomarkers to improve patient selection. Another limitation is potential center-level heterogeneity, as randomization and statistical analyses were not stratified. Given the modest sample size and limited enrollment at several centers, modeling center effects would likely be unstable and were not performed. Future trials should incorporate stratified randomization or statistical models that account for center-level variation. Lastly, the study population consisted almost entirely of Han Chinese patients, which may limit the generalizability of the findings to other ethnic populations. Validation in more diverse populations is warranted.

## Conclusions

Among patients with acute ischemic stroke due to BAO undergoing EVT, this study did not provide conclusive evidence regarding the effect of oral minocycline on early neurological outcomes. Larger trials, using the commonly employed 90-day mRS as the primary outcome and incorporating adequate sample size, extended treatment duration, and prespecified center-level stratification, are required to determine whether minocycline offers clinical benefit.

## Supplementary Information

Below is the link to the electronic supplementary material.

Supplementary Material 1: Additional file 1: Original protocol (version 1.0), Final protocol (version 3.0) and Summary of changes between versions 1 and 3.

Supplementary Material 2: Additional file 2: Original statistical analysis plan (version 1.0), Final statistical analysis plan (version 3.0) and Summary of changes between versions 1 and 3.

Supplementary Material 3: Additional file 3: Tables S1–S10 and Figs. S1–S2.

## Data Availability

Data will be available for non-commercial purpose and after approval of a study proposal by the executive committee 12 months after publication. Data access requests should be sent to jiangwen@fmmu.edu.cn.

## Abbreviations

EVT: endovascular therapy
e-NIHSS: expanded National Institutes of Health Stroke Scale
sICH: symptomatic intracranial hemorrhage
BAO: Basilar artery occlusion
BBB: blood brain barrier
DSMB: data and safety management board
VAs: vertebral arteries
CTA: computed tomographic angiography
MRA: magnetic resonance angiography
mRS: modified Rankin Scale
pc-ASPECTS: posterior circulation Acute Stroke Prognosis Early CT score
PMI: Pons-midbrain-index
AEs: adverse events
SAEs: serious adverse events
mITT: modified intention-to-treat
PP: per-protocol
FR: futile recanalization
