# Supplementary material for: Minocycline for acute basilar artery occlusion stroke undergoing endovascular treatment: a randomized, evaluator-blinded, open-label, pilot trial
Source: BMC Med. 2026 Jul 27;24:580. doi: 10.1186/s12916-026-05092-4 (PMC13688725; doi:10.1186/s12916-026-05092-4)
Supplement: Supplementary file 1 [file 12916_2026_5092_MOESM1_ESM.docx]

**This supplement contains the following items**

**1. Protocol**

**a. Original protocol (first version submitted to ethical committee), version 1.0 (p 2 - 39)**

**b. Final protocol, version 3.0 (p 40 - 82)**

**c. Summary of changes between versions 1 and 3 (p 83 - 92)**

**TRAIL PROTOCOL**

**Minocycline for acute basilar artery occlusion stroke undergoing endovascular treatment: a randomized, evaluator-blinded, open-label, pilot trial**

**27th June 2022**

**Version 1.0**

**TABLE OF CONTENTS**

[**1. Abbreviations and Definitions of Terms	1**](#_Toc206973994)

[**2. Trail Summary	2**](#_Toc206973995)

[**3. Background and Rationale	8**](#_Toc206973996)

[**4. Trail Methods	11**](#_Toc206973997)

[**5. Other information	21**](#_Toc206973998)

[**6. Appendix	22**](#_Toc206973999)

[**7. References:	34**](#_Toc206974000)

| **Abbreviations and Definitions of Terms** | |
| --- | --- |
| **EVT** | Endovascular treatment |
| **BAO** | Basilar artery occlusion |
| **e-NIHSS** | expanded National Institutes of Health Stroke Scale |
| **sICH** | symptomatic Intracranial Hemorrhage |
| **mRS** | modified Rankin Scale |
| **NIHSS** | National Institutes of Health Stroke Scale |
| **mBI** | modified Barthel index |
| **mTICI** | modified Thrombolysis In Cerebral Infarction |
| **ICU** | Intensive Care Unit |
| **VA** | vertebral arteries |
| **CTA** | Computed tomography angiography |
| **MRA** | magnetic resonance angiography |
| **pc-ASPECTS** | posterior circulation Alberta Stroke Program Early CT score |
| **DWI** | diffusion-weighted imaging |
| **NCCT** | non-contrast cranial computed tomography |
| **PMI** | Pons-midbrain-index |
| **DSA** | digital subtraction angiography |
| **CIs** | confidence intervals |
| **RCT** | randomized controlled trial |
| **FR** | futile recanalization |
| **AIS** | acute ischemic stroke |
| **ITT** | Intention-to-Treat |
| **PP** | Per-Protocol |

**Trail Summary**

| **Protocol title** | Effects of Minocycline on Patients with Acute Ischemic Stroke Undergoing Endovascular Treatment due to Basilar Artery Occlusion (MIST-B): the study protocol for an evaluator-blinded, randomized, open-label, proof of concept trial. |
| --- | --- |
| **Short Title** | MIST-B |
| **Protocol version** | Version 1.0, 27.06.2022 |
| **Trial registration** | Clinicaltrials.gov (NCT05512910) |
| **Background and Objective** | Successful recanalization after endovascular treatment (EVT) has been achieved in 85% of the patients with acute basilar artery occlusion (BAO), while less than half of them had good functional outcomes. Minocycline is a potential cytoprotectant with synergistic combination of antioxidant, anti-inflammatory, anti-apoptotic and protection of blood-brain barrier. Our trial aims to evaluate the safety and efficacy of adjunctive oral minocycline to EVT for patients with acute BAO. |
| **Study design** | An evaluator-blinded, randomized, open-label, proof of concept trial |
| **Outcomes** | The primary effectiveness outcome is proportion of modified Rankin Scale (mRS) 0 to 3 at 90 days from randomization.  The primary safety outcome is the incidence of symptomatic intracranial hemorrhage (sICH) at 24 hours.  Secondary efficacy outcomes include:  1.Ordinal Shift analysis of mRS toward a better functional outcome at 90 days.  2.Dichotomized mRS scores (0-2 vs. 3-6; 0-1 vs. 2-6) at 90(-14/+14) days from randomization.  3. National Institutes of Health Stroke Scale (NIHSS) score at baseline, 24 hours, 5 days, 30 days and 90 days from randomization.  4. expanded National Institutes of Health Stroke Scale (e-NIHSS) score at baseline, 24 hours, 5 days, 30 days and 90 days from randomization.  5. modified Barthel index (mBI) scores at baseline, 30 days and 90 days from randomization.  6.Incidence of sICH at 3 days from randomization.  7.Mortality at 90 days from randomization.  8.Changes in infarct volume from baseline to 5 days.  9.Proportion of modified Thrombolysis In Cerebral Infarction(mTICI) 2b-3 after endovascular treatment (Binary data).  10.Intensive care outcomes are the following: (1) Length of Intensive Care Unit (ICU) stay and hospital stay; (2) Time of mechanical ventilation or non-invasive ventilation at 5 days from randomization; (3) Pneumonia at 5 days from randomization.  11.Lab hematology outcomes are the following: Change in percentage of the lymphocyte subpopulations, matrix metalloproteinase-9, IL-6, IL-10, TNF-α, leucocytes, percentage of neutrophilic granulocyte and absolute neutrophil value at 5 days. |
| **Inclusion /**  **Exclusion criteria** | Inclusion criteria:  1) Age 18 years or older.  2) Patients had acute symptoms and signs compatible with acute ischemia stroke due to BAO or intracranial segments of vertebral arteries (VA) resulting in no flow to the basilar artery (eg, functional BAO).  3) Last known well to groin puncture between 0 to 24 hours, whether or not patients had thrombolysis with rt-PA.  4) Pre-stroke mRS score of 0-1.  5) Baseline e-NIHSS score ≥ 6.  6) Neuroimaging Inclusion Criteria: (1) Proven large vessel occlusion in BAO or VA-V4 occlusion (mTICI score 0-1) determined by Computed tomography angiography (CTA) /magnetic resonance angiography (MRA); (2) posterior circulation Alberta Stroke Program Early CT score (pc-ASPECTS) ≥ 5 determined by diffusion-weighted imaging (DWI) or non-contrast cranial computed tomography (NCCT); (3) Pons-midbrain-index (PMI) of < 3.  7) Final mTICI score of 2b or 3 in BAO based on last digital subtraction angiography (DSA) run.  8) Signed Informed Consent obtained.  Exclusion criteria:  1) Age < 18 years old.  2) Complete cerebellar infarct with significant mass effect or has the imaging features of acute hydrocephalus in NCCT.  3) Intracranial hemorrhage at baseline assessment.  4) Previous stroke in the past 90 days.  5) Cardiopulmonary resuscitation was performed within 10 days prior to onset.  6) Known hereditary or acquired hemorrhagic diathesis, coagulation factor deficiency, international normalized ratio > 3, or platelet < 40×10^9^/L.  7) Glucose < 2.2 mmol/L or > 22 mmol/L.  8) Systolic blood pressure persistently >185mmHg or diastolic blood pressure persistently >110mmHg despite antihypertensive intervention.  9) Acute or chronic renal failure of CKD grade 3-4.  10) Known allergy or hypersensitivity to contrast dye or tetracycline group of drugs.  11) Epileptic seizure at symptom onset.  12) Life expectancy (except for stroke) < 3 months.  13) Female who is pregnancy or breastfeeding, or whom do not use effective contraception at childbearing age.  14) Pre-existing mental illness that interferes with neurological evaluation.  15) Known current participation in another clinical investigation with experimental drug.  16) Unlikely to be available for 90 days follow-up. |
| **Measurements and procedures** | Patients presenting with posterior circulation acute ischemic stroke (AIS) due to occlusion in BAO or intracranial segments of VAs are enrolled. Randomization will be performed within 24 hours from symptom onset or last known well in patients meeting all in- and exclusion criteria. Forty patients will be randomized (1:1) to either EVT only or oral minocycline adjunctively to EVT (20 per group).  All these participants will be followed up for 3 months. All patients undergo assessment of the e-NIHSS, NIHSS and mRS at baseline, 24 hours, 5 days, 30 days and 90 days from randomization. All patients undergo assessment of the mBI at baseline, 30 days and 90 days from randomization. At 5 days, MRI is repeated to determine changes in infarct volume. A treatment-blinded person will assess the outcomes dependency. |
| **Intervention** | Both groups will receive endovascular thrombectomy and standard medical.  Patients allocated to the adjunctive minocycline group will receive oral minocycline in addition to endovascular treatment and other standard medical. After randomization, oral minocycline should be given as soon as possible after randomization, ideally before the recanalization during EVT. The first dose of minocycline will be administered 200 mg orally, followed by 100 mg every 12 hours times for a total of 5 days. |
| **Trial Duration** | 15 months |
| **Trial Schedule** | Submission to Ethical Committee in China: 07/2022  10/2022: First-Patient-In (planned)  12/2023: Last-Patient-In (planned) |
| **Statistical Consideration** | The primary analysis will be performed in the intention-to-treat population. Continuous outcomes will be analyzed using linear regression or generalized linear models as appropriate. Categorical outcomes will be analyzed using modified Poisson regression models. Ordinal outcomes will be analyzed using multivariable ordinal logistic regression. The effect parameter will be reported as the risk ratio, the common odds ratio or mean differences as appropriate, along with its 95% CI. |
| **Sample size** | The primary hypothesis is that patients receiving adjunctive minocycline in addition to EVT within 24 hours after stroke onset, will have a higher proportion of functional independence (mRS 0-3) at 90 days. Considering the nature of exploring study in our trail, we plan to enroll 40 patients (20 patients in each group). |
| **GCP Statement** | The study adheres to the latest principles of the Declaration of Helsinki and follows the Good Clinical Practice guidelines. |

# **1. Background and Rationale**

Two recent randomized controlled trials (RCTs) in posterior circulation demonstrated that endovascular treatment (EVT) confers significant benefits over medical therapy alone in patients with acute basilar artery occlusion (BAO)^[1, 2]^. However, a high proportion of patients (55%) continue to experience unfavorable functional outcomes (modified Rankin Scale [mRS] 4–5) or had died by the 90-day follow-up period) in a meta-analysis of the four recent posterior circulation trials^[3]^. The high incidence of futile recanalization (FR) in the posterior circulation might be highly associated with the poor prognosis after patients with acute BAO received EVT^[4]^.

The exact pathophysiological mechanisms underlying FR remain uncertain, but they may involve several factors, including the no-reflow phenomenon, early arterial re-occlusion, inadequate collateral circulation, hemorrhagic transformation, impaired cerebrovascular autoregulation, and a larger area of hypoperfusion^[5]^. Minocycline has been previously demonstrated individually to reduce infarction in animal models of stroke, and have potentially mechanisms of antioxidant, anti-inflammatory, anti-apoptotic and protection of blood-brain barrier^[6, 7]^. There is still no consensus whether minocycline have a clear advantage in neurologic outcomes because data have been limited to small randomized trials in acute ischemic stroke (AIS) without EVT^[8-10]^. Considering the effective in immediate tissue reperfusion of thrombectomy techniques, the adjunctive minocycline with EVT might be a viable pathway to improve neurological deficits early after stroke in patients with AIS due to BAO.

**1.1 Research question and research in context**

The purpose of current study is to evaluate the effects of adjunctive oral minocycline in patients with AIS due to BAO in the setting of receiving endovascular treatment.

**1.2 Clinical evidence to date**

*Limitations of EVT on large-vessel occlusions*

Futile recanalization is a crucial factor that impaired efficacy of EVT. A better understanding of the predictors of FR, such as disease severity at baseline, reperfusion delay, incomplete recanalization, may provide important insights into management of the disease^[11]^. The pathological mechanism of FR is still unclear, but reperfusion injury and its subsequent inflammatory are involved. It may be worth exploring the use of a cytoprotectant as a viable intervention to mitigate subsequent injury caused by ischaemia and reperfusion.

*Evidences of anti-inflammatory therapy on large-vessel occlusions with EVT*

Preclinical models suggested potential benefits of methylprednisolone, fingolimod, natalizumab, and cyclosporine in stroke treatment, yet none have been validated by clinical trials^[12-16]^. Minocycline is a second-generation tetracycline capable of inhibiting bacterial protein synthesis with high blood-brain barrier permeability. It affects numerous molecular pathways that have been implicated in neurologic injury, including abilities to inhibit microglial activation, reduce T-cell migration, scavenge free radicals, decrease chemokine expression, and inhibit matrix metalloproteases, minimizing degradation of the basal lamina and stabilizing blood brain barrier integrity^[17]^. Animal model studies and several researches showed its effect on reducing infarction of stroke, while the evidence was limited due to sample size and trail design. MIST-B distinguishes itself from previously researches primarily through the application of minocycline on acute stroke patients receiving endovascular treatment. Considering the effective in immediate tissue reperfusion of thrombectomy techniques, the adjunctive minocycline with EVT might be a viable pathway to reduce FR.

*Interpretation of existing evidence*

Effective delivery to ischemic tissue is a key determinant of minocycline's potential utility following EVT, where successful recanalization may facilitate drug access^[18]^. Given the compelling biological rationale linking minocycline's mechanisms to ischemia/reperfusion injuries, rigorous evaluation as an adjunctive therapy to EVT is warranted^[19]^. In summary, there is clinical equipoise regarding whether minocycline in addition to EVT has a positive treatment effect in AIS patients with a BAO and we therefore propose a multi-center, evaluator-blinded, randomized, open-label, comparison of oral minocycline plus EVT to EVT alone with the mRS at 90 days as the main outcome parameter.

# **2. Trail Methods**

**2.1 Design**

Minocycline for acute Ischemic Stroke undergoing endovascular Treatment due to Basilar artery occlusion (MIST-B) is an evaluator-blinded, randomized, open-label, proof of concept trial. The aim of the MIST-B is to assess the safety and efficacy of adjunctive oral minocycline to EVT for patients with acute BAO. The study has a planned duration of 15 months to recruit 40 patients in China. The study patient flow outline is shown in **Figure 1**. All enrolled patients will be randomized with 1:1 to EVT only or oral minocycline adjunctively to EVT within 24 hours of symptom onset and receive continuous application for 5 days. All these participants will receive standard medical care afterwards and be followed up for 3 months. The study adheres to the latest principles of the Declaration of Helsinki and follows the Good Clinical Practice guidelines. The MIST-B trail (www.clinicaltrials.gov. NCT05512910) has received approval from The Ethical Committee of Xijing Hospital (XJLL-KY2022184).

Figure 1. MIST-B flow chart.

**2.2 Patient population**

Patients presenting with posterior circulation AIS due to occlusion in BAO or intracranial segments of vertebral arteries (VAs) are enrolled. Patients will be randomized within 24 hours from symptom onset or last known well.

Inclusion criteria:

1) Age 18 years or older.

2) Patients had acute symptoms and signs compatible with acute ischemia stroke due to BAO or intracranial segments of VAs resulting in no flow to the basilar artery (eg, functional BAO).

3) Last known well to groin puncture between 0 to 24 hours, whether or not patients had thrombolysis with rt-PA.

4) Pre-stroke mRS score of 0-1.

5) Baseline expanded National Institutes of Health Stroke Scale (e-NIHSS) score ≥ 6.

6) Neuroimaging Inclusion Criteria: (1) Proven large vessel occlusion in BAO or VA-V4 occlusion (modified Thrombolysis In Cerebral Infarction [mTICI] score 0-1) determined by Computed tomography angiography (CTA) /magnetic resonance angiography (MRA); (2) posterior circulation Alberta Stroke Program Early CT score (pc-ASPECTS) ≥ 5 determined by diffusion-weighted imaging (DWI) or non-contrast cranial computed tomography (NCCT); (3) Pons-midbrain-index (PMI) of < 3.

7) Final mTICI score of 2b or 3 in BAO based on last digital subtraction angiography (DSA) run.

8) Signed Informed Consent obtained.

Exclusion criteria:

1) Age < 18 years old.

2) Complete cerebellar infarct with significant mass effect or has the imaging features of acute hydrocephalus in NCCT.

3) Intracranial hemorrhage at baseline assessment.

4) Previous stroke in the past 90 days.

5) Cardiopulmonary resuscitation was performed within 10 days prior to onset.

6) Known hereditary or acquired hemorrhagic diathesis, coagulation factor deficiency, international normalized ratio > 3, or platelet < 40×10^9^/L.

7) Glucose < 2.2 mmol/L or > 22 mmol/L.

8) Systolic blood pressure persistently >185mmHg or diastolic blood pressure persistently >110mmHg despite antihypertensive intervention.

9) Acute or chronic renal failure of CKD grade 3-4.

10) Known allergy or hypersensitivity to contrast dye or tetracycline group of drugs.

11) Epileptic seizure at symptom onset.

12) Life expectancy (except for stroke) < 3 months.

13) Female who is pregnancy or breastfeeding, or whom do not use effective contraception at childbearing age.

14) Pre-existing mental illness that interferes with neurological evaluation.

15) Known current participation in another clinical investigation with experimental drug.

16) Unlikely to be available for 90 days follow-up.

**2.3 Randomization**

Block randomization will be used to randomize subjects. After occlusion of basilar artery has been confirmed and written informed consent has been obtained, patients will be immediately randomly assigned to adjunctive minocycline group or control group in a 1:1 ratio. Randomization will not be stratified by other parameters. The randomization sequence will be generated using SAS V.9.4 software in the independent statistical group. Researches had the access to the randomization results except the independent assessor at each trial site.

**2.4 Intervention**

Both groups will receive endovascular thrombectomy and standard medical. Patients allocated to the adjunctive minocycline group will receive oral minocycline in addition to endovascular treatment and other standard medical. After randomization, oral minocycline should be given as soon as possible, ideally before the recanalization during EVT. The first dose of minocycline will be administered 200 mg orally, followed by 100 mg every 12 hours times for a total of 5 days. If the patient is considered at risk for aspiration or unable to swallow based on a swallowing evaluation, minocycline will be administered via a feeding tube.

Documentation of Deviations: Investigators must adhere strictly to the study protocol. Deviation from the treatment regimen is defined as the subject not receiving any assigned treatment, unless the subject met the criteria for treatment discontinuation. All protocol deviations will be documented in a protocol deviation log, which will be reviewed regularly by the trial steering committee to ensure consistency in applying the exclusion criteria. The rationale for excluding each participant will be documented in detail.

**2.5 Study assessment and treatment masking**

Treatment allocation is known only to the patient or their families, and their treating clinicians. An independent assessor in charge of assessment of baseline data, in-hospital data and standardized visits will be blinded to treatment assignment and actual treatment. These assessments are recorded, and a comprehensive follow-up report is compiled. Centralized interpretation will be performed for all study-related clinical outcomes and imaging data. The accuracy of outcome events is finally assessed by Data and Safety Management Board (DSMB). The DSMB will be blinded to treatment allocation, drug use, and participant identity.

During the hospital stay, all concomitant treatments, adverse events, and any serious adverse events will be recorded. For patients discharged prior to 5-day visit, clinical assessments at discharge were recorded. At 30 days and 90 days from randomization, a face-to-face or telephone follow-up will be conducted by independent certified assessors. If all attempts to reach the patients are exhausted without success, they will be classified as lost to follow-up upon finalization of the case report form. The day-30 outcome was allowed a time window of ±7 days, and the day-90 outcome a window of ±14 days from the target date.

All patients undergo assessment of the e-NIHSS, National Institutes of Health Stroke Scale (NIHSS) and mRS at baseline, 24 hours, 5 days, 30 days and 90 days from randomization. All patients undergo assessment of the modified Barthel Index (mBI) at baseline, 30 days and 90 days from randomization. At 5 days, MRI is repeated to determine changes in infarct volume.

**2.6 Contents of Investigational Product Kit**

Minocycline Hydrochloride Capsules are manufactured by Hisun Pharmaceutical Group Co., Ltd., Hangzhou, China. All study medication will be provided by Xijing Hospital.

**2.7 End of Study and Length of Study**

The study will conclude on the date of the last patient's last visit. The conclusion is anticipated to take place 90 days post-randomization of the final participant. The entire study, from the initial patient screening to the last patient's last visit, is estimated to complete within 15 months.

**2.8 Primary outcomes**

The primary effectiveness outcome is proportion of mRS 0 to 3 at 90 days from randomization.

The primary safety outcome is the incidence of symptomatic intracranial hemorrhage (sICH) at 24 hours from randomization.

**2.9 Secondary outcomes**

1.Ordinal Shift analysis of mRS toward a better functional outcome at 90 days.

2.Dichotomized mRS scores (0-2 vs. 3-6; 0-1 vs. 2-6) at 90 days from randomization.

3.NIHSS at baseline, 24 hours, 5 days, 30 days and 90 days from randomization.

4.e-NIHSS at baseline, 24 hours, 5 days, 30 days and 90 days from randomization.

5.mBI scores at baseline, 30 days and 90 days from randomization.

6.Incidence of sICH at 3 days from randomization.

7.Mortality at 90 days from randomization.

8.Changes in infarct volume from baseline to 5 days.

9. Proportion of mTICI 2b-3 after endovascular treatment.

10. Intensive care outcomes are the following: (1) Length of Intensive Care Unit (ICU) stay and hospital stay; (2) Time of mechanical ventilation or non-invasive ventilation at 5 days from randomization; (3) Pneumonia at 5 days from randomization.

10.Lab hematology outcomes are the following: Change in percentage of the lymphocyte subpopulations, matrix metalloproteinase-9, IL-6, IL-10, TNF-α, leucocytes, percentage of neutrophilic granulocyte and absolute neutrophil value at 5 days.

**2.10 Adverse events and Safety**

Laboratory assessments will be performed to monitor adverse events at baseline, 24 hours, 5 days, 30 days and 90 days from randomization. CT is repeated to determine intracranial hemorrhages at 24 hours after randomization, or anytime a neurological deterioration on the NIHSS. The abnormal laboratory results or intracranial hemorrhages in CT will be evaluated by the safety committee, and be assessed their relation to the minocycline. Minocycline should be withdrawn if white blood cell counts below 3.0 * 10^9/L, neutrophil counts below 1.5 * 10^9/L, platelet counts below 100 * 10^9/L, hemoglobin levels below 30g/L, or marked increases in bilirubin, aspartate aminotransferase, alanine aminotransferase, creatinine, or urea. All adverse events will be meticulously recorded, and serious adverse events will be promptly reported to both the safety and ethics committees within 24 hours. Participants who experience serious harm due to their involvement in the study will receive appropriate financial compensation.

**2.11 Data Quality and Monitoring**

Data Monitoring Committee will be established to uniform data quality across participating centers. The committee will prepare periodic reports on data integrity, protocol adherence, and patient outcomes. The Principal Investigator will oversee quality control to ensure that the trial is conducted in accordance with the protocol. A data management plan will be implemented, and all information required for monitoring and analysis of the study will be stored securely in a locked filing cabinet within a restricted-access office. The digital records will be safeguarded on a computer that is secured with a password and a firewall. Study data will be regularly monitored for protocol compliance, data accuracy and completeness onsite by the sponsor or their delegates, in accordance with the study’s monitoring plan. The data controller for the study will be the trial's sponsor.

**2.12 Sample size estimates**

The primary hypothesis is that patients receiving adjunctive minocycline in addition to EVT within 24 hours after stroke onset, will have a higher proportion of functional independence (mRS 0-3) at 90 days. Considering the nature of exploring study in our trail, we plan to enroll 40 patients (20 patients in each group).

**2.13 Statistical analyses**

Intention-to-Treat (ITT) population, Per-Protocol (PP) population and Safety population are planned for the studies. The primary analysis will be performed in the intention-to-treat population. Continuous variables will be reported as means (SD), and categorical variables will be presented as percentages. The Kolmogorov-Smirnoff test is used to assess the normality of the continuous variables. Differences in baseline characteristics between the two groups will be compared by either Student’s t test or Mann-Whitney U test for continuous variables and with the chi-square test for categorical variables. Differences in primary outcomes between the two groups will be analyzed using linear models. The mean differences and 95% confidence intervals (CIs) of coefficients in the model will be presented to describe differences between the two groups.

Missing values for baseline characteristics will be reported. Missing baseline characteristics will be imputed using regression imputation. All hypothesis tests will adopt a two-sided alpha level of significant set at 0.05 (α=0.05).

**2.14 Patient Involvement and Informed consent**

We recruit participants through Green Channel of Acute Ischemic Stroke and conduct trials with sufficient information on the study design. All participants will be thoroughly informed about the study's goals, the potential side effects associated with the procedures and interventions, who will have access to their personal information, and any compensation available for individuals who may experience harm as a result of participating in the trial. It will be clearly emphasized that participation is entirely voluntary and that participants have the right to withdraw from the study at any time without any influence on their subsequent care. There is one informed consent document required for MIST-B. Patients or the public involves in the design, or conduct, or reporting, or dissemination plans of our research.

**2.15 Ethics and Dissemination**

Central ethics approval was obtained from The Ethics Committee of Xijing Hospital, Xi’an, China (XJLL-KY2022184). The trail is registered at Clinicaltrials.gov (NCT05512910). Any modifications to the protocol which may impact the conduct of the study, potential benefit of the participant, or may affect participant safety, will require a formal amendment to the protocol. Such amendments will be agreed on by the study steering committee and approved by the ethics committee prior to implementation. A report describing the results of the study will be submitted to international meetings and peer-reviewed journals.

# **3 Other information**

**3.1 Trial site and Investigators**

**Chief Investigator:**

Dr. Wen Jiang, Xijing Hospital, Department of Neurology, Fourth Military Medical University;

**3.2 Statisticians**

Pro. Lei Shang and Pro. Ling Wang, Department of Health Statistics, Fourth Military Medical University.

**3.3 Independent assessors**

Dr. Le Wang, Department of Neurology, Xijing Hospital, Fourth Military Medical University;

**3.4 Data and Safety Management Board (DSMB)**

Dr. Zhirong Liu, Department of Neurology, Xijing Hospital, Fourth Military Medical University;

Dr. Hongzeng Li, Department of Neurology, Tangdu Hospital, Fourth Military Medical University;

Pro. Lei Shang, Department of Health Statistics, Fourth Military Medical University.

# **4. Appendix**

**4.1 Expanded National Institutes of Health Stroke Scale (e-NIHSS)**

The e-NIHSS is a modified NIHSS focused on posterior circulation strokes, with the advantage to improve sensitivity of outcome assessment. The e-NIHSS consists of 13 items with scores from 0 to 42, with 5 categories added on the existing items 4, 6, and 9(showed as the bolded words in below scale).

| Instructions | Scale definition |
| --- | --- |
| 1a. Level of consciousness. The investigator must choose a response if a full evaluation is prevented by such obstacles as an endotracheal tube, language barrier, orotracheal trauma/bandages. A 3 is scored only if the patient makes no movement (other than reflexive posturing) in response to noxious stimulation. | 0=Alert; keenly responsive.  1=Not alert; but arousable by minor stimulation to obey, answer, or respond.  2=Not alert; required repeated stimulation to attend, or is obtunded and requires strong or painful stimulation to make movements (not stereotyped).  3=Responds only with reflex motor or autonomic effects or totally unresponsive, flaccid and areflexic. |
| 1b. LOC Questions: The patient is asked the month and his/her age. The answer must be correct – there is not partial credit for being close. Phasic and stuporous patients who do not comprehend the questions will score 2. Patients unable to speak because of endotracheal intubation, orotracheal trauma, severe dysarthria from any cause, language barrier, or any other problem not secondary to aphasia are given a 1. It is important that only the initial answer be graded and that the examiners not “help” the patient with verbal or nonverbal clues. | 0=Answers both questions correctly.  1=Answers one question correctly.  2=Answers neither question correctly. |
| 1c. LOC Commands: The patient is asked to open and close the eyes and then to grip and release the non-paretic hand. Substitute another one step command if the hand cannot be used. Credit is given if an unequivocal attempt is made but not completed due to weakness. If the patient does not respond to command, the task should be demonstrated to him or her (pantomime), and the result scored (i.e. follows none, one or two commands). Patients with trauma, amputation, or other physical impediments should be given suitable one-step commands. Only the first attempt is scored. | 0=Performs both tasks correctly.  1=Performs one task correctly.  2=Performs neither task correctly. |
| 2. Best Gaze: Only horizontal eye movements will be tested. Voluntary or reflexive (oculocephalic) eye movements will be scored, but caloric testing is not done. If the patient has a conjugate deviation of the eyes that can be overcome by voluntary or reflexive activity, the score will be a 1. If a patient has an isolated peripheral nerve paresis (CN III, IV or VI), score a 1. Gaze is testable in all aphasic patients. Patients with ocular trauma, bandages, preexisting blindness, or other disorder of visual acuity or fields should be tested with reflexive movements, and a choice made by the investigator. Establishing eye contact and then moving about the patient from side to side will occasionally clarify the presence of a partial gaze palsy. | 0= Normal.  1= Partial gaze palsy; gaze is abnormal in one or both eyes, but forced deviation or total gaze paresis is not present.  **1 = nystagmus and/or Horner’s syndrome**  2= Forced deviation; or total gaze paresis not overcome by the oculocephalic maneuver. |
| 3. Visual: Visual fields (upper and lower quadrants) are tested by confrontation, using finger counting or visual threat, as appropriate. Patients may be encouraged, but if they look at the side of the moving finger appropriately, this can be scored as normal. If there is unilateral blindness or enucleation, visual fields in the remaining eye are scored. Score 1 only if a clearcut asymmetry, including quadrantanopia, is found. If patient is blind from any cause, score 3. Double simultaneous stimulation is performed in this case. If there is extinction, the patient receives a 1, and the results are used to respond to item 11. | 0=No visual loss.  1= Partial hemianopia.  2= Complete hemianopia.  3= Bilateral hemianopia (blind including cortical blindness) |
| 4. Facial palsy: Ask or use pantomime to encourage the patient to show teeth or raise eyebrows and close eyes. Score symmetry of grimace in response to noxious stimuli in the poorly response or non-comprehending patient. If facial trauma/bandages, orotracheal tube, tape or other physical barriers obscure the face, these should be removed to the extent possible. | 0 = Normal symmetrical movements.  1= Minor paralysis (flattened nasolabial fold, asymmetry on smiling)  2= Partial paralysis (total or near-total paralysis of lower face)  3= Complete paralysis of one or both sides (absence of facial movement in the upper and lower face).  **3 = deficit of IX nerve (soft palate paralysis)**  **3 = deficit of XII nerve** |
| 5. Motor arm: The limb is placed in the appropriate position: extend the arms (palms down) 90 degrees (if sitting) or 45 degrees (if supine). Drift is scored if the arm falls before 10 seconds. The aphasic patient is encouraged using urgency in the voice and pantomime, but not noxious stimulation. Each limb is tested in turn, beginning with the non-paretic arm. Only in the case of amputation or joint fusion at the shoulder, the examiner should record the score as untestable (UN), and clearly write the explanation for this choice. | 0= No drift; limb holds 90 (or 45) degrees for full 10 seconds.  1= Drift; limb holds 90 (or 45) degrees, but drifts down before full 10 seconds; does not hit bed or other support.  2= Some effort against gravity; limb cannot get to or maintain (if cued) 90 (or 45) degrees, drifts down to bed, but has some effort against gravity.  3= No effort against gravity; limb falls.  4= No movement.  UN = Amputation or joint fusion: explain:  5a = Left Arm.  5b = Right arm. |
| 6. Motor leg: The limb is placed in the appropriate position: hold the leg at 30 degrees (always tested supine). Drift is scored if the leg falls before 5 seconds. The aphasic patient is encouraged using urgency in the voice and pantomime, but not noxious stimulation. Each limb is tested in turn, beginning with the non-paretic leg. Only in the case of amputation or joint fusion at the hip, the examiner should record the score as untestable (UN), and clearly write the explanation for this  choice. | 0= No drift; leg holds 30-degree position for full 5seconds.  1= Drift; leg falls by the end of the 5-second period but does not hit bed.  2= Some effort against gravity; leg falls to bed by 5 seconds, but has some effort against gravity.  3= No effort against gravity; leg falls to bed immediately.  4= No movement.  UN = Amputation or joint fusion: explain:  6a. Left Leg  6b. Right Leg. |
| 7. Limb ataxia: This item is aimed at finding evidence of a unilateral cerebellar lesion. Test with eyes open. In case of visual defect, ensure testing is done in intact visual field. The finger-nose-finger and heel-shin tests are performed on both sides, and ataxia is scored only if present out of proportion to weakness. Ataxia is absent in the patient who cannot understand or is paralyzed. Only in the case of amputation or joint fusion, the examiner should record the score as untestable (UN), and clearly write the explanation for this choice. In case of blindness, test by having the patient touch nose from extended arm position. | 0=Absent.  1= Present in one limb.  **1 = imbalance in Romberg position.**  2= Present in two limbs.  **2 = trunk ataxia or retro- or lateropulsion.**  UN = Amputation or joint fusion: explain: |
| 8. Sensory: Sensation or grimace to pinprick when tested, or withdrawal from noxious stimulus in the obtunded or aphasic patient. Only sensory loss attributed to stroke is scored as abnormal and the examiner should test as many body areas (arms [not hands], legs, trunk, face) as needed to accurately check for hemisensory loss. A score of 2, 'severe or total sensory loss', should only be given when a severe or total loss of sensation can be clearly demonstrated. Stuporous and aphasic patients will, therefore, probably score 1 or 0. The patient with brainstem stroke who has bilateral loss of sensation is scored 2. If the patient does not respond and is quadriplegic, score 2. Patients in a coma (item 1a=3) are automatically given a 2 on this item. | 0= Normal; no sensory loss.  1= Mild-to-moderate sensory loss; patients feels pinprick is less sharp or is dull on the affected side; or there is a loss of superficial pain with pinprick, but patient is aware of being touched.  2= Severe to total sensory loss; patient is not aware of being touched in the face, arm and leg. |
| 9. Best language: A great deal of information about comprehension will be obtained during the preceding sections of the examination. For this scale item, the patient is asked to describe what is happening in the attached picture, to name the items on the attached naming sheet and to read from the attached list of sentences. Comprehension is judged from responses here, as well as to all of the commands in the preceding general neurological exam. If visual loss interferes with the tests, ask the patient to identify objects placed in the hand, repeat, and produce speech. The intubated patient should be asked to write. The patient in a coma (item 1a=3) will automatically score 3 on this item. The examiner must choose a score for the patient with stupor or limited cooperation, but a score of 3 should be used only if the patient is mute and follows no one-step commands. | 0= No aphasia; normal  1= Mild-to-moderate aphasia; some obvious loss of fluency or facility of comprehension, without significant limitation on ideas expressed or form of expression. Reduction of speech and/or comprehension, however, makes conservation about provided materials difficult or impossible. For example, in conversation about provided materials, examiner can identify picture or naming card content from patient's response.  2= Severe aphasia; all communication is through fragmentary expression; great need for inference, questioning, and guessing by the listener. Range of information that can be exchanged is limited; listener carries burden of communication. Examiner cannot identify materials provided from patient response.  3= Mute, global aphasia: no usable speech or auditory comprehension. |
| 10. Dysarthria: If patient is thought to be normal, an adequate sample of speech must be obtained by asking patient to read or repeat words from the attached list. If the patient has severe aphasia, the clarity of articulation of spontaneous speech can be rated. Only if patient is intubated or has other physical barriers to producing speech, the examiner should record the score as untestable (UN), and clearly write an explanation for this choice. Do not tell the patient why he or she is being tested. | 0= Normal.  1= Mild-to-moderate dysarthria; patient slurs at least some words and, at worst, can be understood by some difficulty.  2= Severe dysarthria: patient's speech is so slurred as to be unintelligible in the absence of or out of proportion to any dysphasia, or is mute/anarthric.  UN = Intubated or other physical barrier. |
| 11. Extinction and Inattention (formerly Neglect): Sufficient information to identify neglect may be obtained during the prior testing. If the patient has a severe visual loss preventing visual double simultaneous stimulation, and the cutaneous stimuli are normal, the score is normal. If the patient has aphasia but does appear to attend to both sides, the score is normal. The presence of visual spatial neglect or anosagnosia may also be taken as evidence of abnormality. Since the abnormality is scored only if present, the item is never untestable. | 0= No abnormality.  1= Visual, tactile, auditory, spatial, or personal inattention or extinction to bilateral simultaneous stimulation in one of the sensory modalities.  2= Profound hemi-inattention or extinction to more than one modality; does not recognize own hand or orients to only one side of space. |

**4.2 National Institutes of Health Stroke Scale (NIHSS)**

| **Instructions** | **Scale definition** |
| --- | --- |
| 1a. Level of consciousness. The investigator must choose a response if a full evaluation is prevented by such obstacles as an endotracheal tube, language barrier, orotracheal trauma/bandages. A 3 is scored only if the patient makes no movement (other than reflexive posturing) in response to noxious stimulation. | 0=Alert; keenly responsive.  1=Not alert; but arousable by minor stimulation to obey, answer, or respond.  2=Not alert; required repeated stimulation to attend, or is obtunded and requires strong or painful stimulation to make movements (not stereotyped).  3=Responds only with reflex motor or autonomic effects or totally unresponsive, flaccid and areflexic. |
| 1b. LOC Questions: The patient is asked the month and his/her age. The answer must be correct – there is not partial credit for being close. Phasic and stuporous patients who do not comprehend the questions will score 2. Patients unable to speak because of endotracheal intubation, orotracheal trauma, severe dysarthria from any cause, language barrier, or any other problem not secondary to aphasia are given a 1. It is important that only the initial answer be graded and that the examiners not “help” the patient with verbal or nonverbal clues. | 0=Answers both questions correctly.  1=Answers one question correctly.  2=Answers neither question correctly. |
| 1c. LOC Commands: The patient is asked to open and close the eyes and then to grip and release the non-paretic hand. Substitute another one step command if the hand cannot be used. Credit is given if an unequivocal attempt is made but not completed due to weakness. If the patient does not respond to command, the task should be demonstrated to him or her (pantomime), and the result scored (i.e. follows none, one or two commands). Patients with trauma, amputation, or other physical impediments should be given suitable one-step commands. Only the first attempt is scored. | 0=Performs both tasks correctly.  1=Performs one task correctly.  2=Performs neither task correctly. |
| 2. Best Gaze: Only horizontal eye movements will be tested. Voluntary or reflexive (oculocephalic) eye movements will be scored, but caloric testing is not done. If the patient has a conjugate deviation of the eyes that can be overcome by voluntary or reflexive activity, the score will be a 1. If a patient has an isolated peripheral nerve paresis (CN III, IV or VI), score a 1. Gaze is testable in all aphasic patients. Patients with ocular trauma, bandages, preexisting blindness, or other disorder of visual acuity or fields should be tested with reflexive movements, and a choice made by the investigator. Establishing eye contact and then moving about the patient from side to side will occasionally clarify the presence of a partial gaze palsy. | 0= Normal.  1= Partial gaze palsy; gaze is abnormal in one or both eyes, but forced deviation or total gaze paresis is not present.  2= Forced deviation; or total gaze paresis not overcome by the oculocephalic maneuver. |
| 3. Visual: Visual fields (upper and lower quadrants) are tested by confrontation, using finger counting or visual threat, as appropriate. Patients may be encouraged, but if they look at the side of the moving finger appropriately, this can be scored as normal. If there is unilateral blindness or enucleation, visual fields in the remaining eye are scored. Score 1 only if a clearcut asymmetry, including quadrantanopia, is found. If patient is blind from any cause, score 3. Double simultaneous stimulation is performed in this case. If there is extinction, the patient receives a 1, and the results are used to respond to item 11. | 0=No visual loss.  1= Partial hemianopia.  2= Complete hemianopia.  3= Bilateral hemianopia (blind including cortical blindness) |
| 4. Facial palsy: Ask or use pantomime to encourage the patient to show teeth or raise eyebrows and close eyes. Score symmetry of grimace in response to noxious stimuli in the poorly response or non-comprehending patient. If facial trauma/bandages, orotracheal tube, tape or other physical barriers obscure the face, these should be removed to the extent possible. | 0 = Normal symmetrical movements.  1= Minor paralysis (flattened nasolabial fold, asymmetry on smiling)  2= Partial paralysis (total or near-total paralysis of lower face)  3= Complete paralysis of one or both sides (absence of facial movement in the upper and lower face). |
| 5. Motor arm: The limb is placed in the appropriate position: extend the arms (palms down) 90 degrees (if sitting) or 45 degrees (if supine). Drift is scored if the arm falls before 10 seconds. The aphasic patient is encouraged using urgency in the voice and pantomime, but not noxious stimulation. Each limb is tested in turn, beginning with the non-paretic arm. Only in the case of amputation or joint fusion at the shoulder, the examiner should record the score as untestable (UN), and clearly write the explanation for this choice. | 0= No drift; limb holds 90 (or 45) degrees for full 10 seconds.  1= Drift; limb holds 90 (or 45) degrees, but drifts down before full 10 seconds; does not hit bed or other support.  2= Some effort against gravity; limb cannot get to or maintain (if cued) 90 (or 45) degrees, drifts down to bed, but has some effort against gravity.  3= No effort against gravity; limb falls.  4= No movement.  UN = Amputation or joint fusion: explain:  5a = Left Arm.  5b = Right arm. |
| 6. Motor leg: The limb is placed in the appropriate position: hold the leg at 30 degrees (always tested supine). Drift is scored if the leg falls before 5 seconds. The aphasic patient is encouraged using urgency in the voice and pantomime, but not noxious stimulation. Each limb is tested in turn, beginning with the non-paretic leg. Only in the case of amputation or joint fusion at the hip, the examiner should record the score as untestable (UN), and clearly write the explanation for this  choice. | 0= No drift; leg holds 30-degree position for full 5seconds.  1= Drift; leg falls by the end of the 5-second period but does not hit bed.  2= Some effort against gravity; leg falls to bed by 5 seconds, but has some effort against gravity.  3= No effort against gravity; leg falls to bed immediately.  4= No movement.  UN = Amputation or joint fusion: explain:  6a. Left Leg  6b. Right Leg. |
| 7. Limb ataxia: This item is aimed at finding evidence of a unilateral cerebellar lesion. Test with eyes open. In case of visual defect, ensure testing is done in intact visual field. The finger-nose-finger and heel-shin tests are performed on both sides, and ataxia is scored only if present out of proportion to weakness. Ataxia is absent in the patient who cannot understand or is paralyzed. Only in the case of amputation or joint fusion, the examiner should record the score as untestable (UN), and clearly write the explanation for this choice. In case of blindness, test by having the patient touch nose from extended arm position. | 0=Absent.  1= Present in one limb.  2= Present in two limbs.  UN = Amputation or joint fusion: explain: |
| 8. Sensory: Sensation or grimace to pinprick when tested, or withdrawal from noxious stimulus in the obtunded or aphasic patient. Only sensory loss attributed to stroke is scored as abnormal and the examiner should test as many body areas (arms [not hands], legs, trunk, face) as needed to accurately check for hemisensory loss. A score of 2, 'severe or total sensory loss', should only be given when a severe or total loss of sensation can be clearly demonstrated. Stuporous and aphasic patients will, therefore, probably score 1 or 0. The patient with brainstem stroke who has bilateral loss of sensation is scored 2. If the patient does not respond and is quadriplegic, score 2. Patients in a coma (item 1a=3) are automatically given a 2 on this item. | 0= Normal; no sensory loss.  1= Mild-to-moderate sensory loss; patients feels pinprick is less sharp or is dull on the affected side; or there is a loss of superficial pain with pinprick, but patient is aware of being touched.  2= Severe to total sensory loss; patient is not aware of being touched in the face, arm and leg. |
| 9. Best language: A great deal of information about comprehension will be obtained during the preceding sections of the examination. For this scale item, the patient is asked to describe what is happening in the attached picture, to name the items on the attached naming sheet and to read from the attached list of sentences. Comprehension is judged from responses here, as well as to all of the commands in the preceding general neurological exam. If visual loss interferes with the tests, ask the patient to identify objects placed in the hand, repeat, and produce speech. The intubated patient should be asked to write. The patient in a coma (item 1a=3) will automatically score 3 on this item. The examiner must choose a score for the patient with stupor or limited cooperation, but a score of 3 should be used only if the patient is mute and follows no one-step commands. | 0= No aphasia; normal  1= Mild-to-moderate aphasia; some obvious loss of fluency or facility of comprehension, without significant limitation on ideas expressed or form of expression. Reduction of speech and/or comprehension, however, makes conservation about provided materials difficult or impossible. For example, in conversation about provided materials, examiner can identify picture or naming card content from patient's response.  2= Severe aphasia; all communication is through fragmentary expression; great need for inference, questioning, and guessing by the listener. Range of information that can be exchanged is limited; listener carries burden of communication. Examiner cannot identify materials provided from patient response.  3= Mute, global aphasia: no usable speech or auditory comprehension. |
| 10. Dysarthria: If patient is thought to be normal, an adequate sample of speech must be obtained by asking patient to read or repeat words from the attached list. If the patient has severe aphasia, the clarity of articulation of spontaneous speech can be rated. Only if patient is intubated or has other physical barriers to producing speech, the examiner should record the score as untestable (UN), and clearly write an explanation for this choice. Do not tell the patient why he or she is being tested. | 0= Normal.  1= Mild-to-moderate dysarthria; patient slurs at least some words and, at worst, can be understood by some difficulty.  2= Severe dysarthria: patient's speech is so slurred as to be unintelligible in the absence of or out of proportion to any dysphasia, or is mute/anarthric.  UN = Intubated or other physical barrier. |
| 11. Extinction and Inattention (formerly Neglect): Sufficient information to identify neglect may be obtained during the prior testing. If the patient has a severe visual loss preventing visual double simultaneous stimulation, and the cutaneous stimuli are normal, the score is normal. If the patient has aphasia but does appear to attend to both sides, the score is normal. The presence of visual spatial neglect or anosagnosia may also be taken as evidence of abnormality. Since the abnormality is scored only if present, the item is never untestable. | 0= No abnormality.  1= Visual, tactile, auditory, spatial, or personal inattention or extinction to bilateral simultaneous stimulation in one of the sensory modalities.  2= Profound hemi-inattention or extinction to more than one modality; does not recognize own hand or orients to only one side of space. |

**4.3 Modified Rankin Scale (mRS)**

**0** No symptoms at all

**1** No significant disability despite symptoms: able to carry out all usual duties and activities

**2** Slight disability: unable to carry out all previous activities but able to look after own affairs without assistance

**3** Moderate disability: requiring some help, but able to walk without assistance

**4** Moderately severe disability: unable to walk without assistance, and unable to attend to own bodily needs without assistance

**5** Severe disability: bedridden, incontinent, and requiring constant nursing care and attention

**6** Death

**4.4 Modified Barthel Index (mBI)**

| **Items** | **Codes** | | | | |
| --- | --- | --- | --- | --- | --- |
| any part of midbrain | 1  Unable to perform task | 2  Attempts task but unsafe | 3  Moderate help required | 4  Minimal help required | 5  Fully independent |
| Personal hygiene | 0 | 1 | 3 | 4 | 5 |
| Bathing self | 0 | 1 | 3 | 4 | 5 |
| Feeding | 0 | 2 | 5 | 8 | 10 |
| Toilet | 0 | 2 | 5 | 8 | 10 |
| Dressing | 0 | 2 | 5 | 8 | 10 |
| Bowel control | 0 | 2 | 5 | 8 | 10 |
| Bladder control | 0 | 2 | 5 | 8 | 10 |
| Stair climbing | 0 | 2 | 5 | 8 | 10 |
| Ambulation | 0 | 3 | 8 | 12 | 15 |
| Wheelchair | 0 | 1 | 3 | 4 | 5 |
| Chair/bed transfers | 0 | 3 | 8 | 12 | 15 |
| Range | 0……………………………………………..100 | | | | |

“Wheelchair” will be scored only if “Ambulation” coded “1” and patient trained in wheelchair management.

**4.5 Posterior Circulation Alberta Stroke Program Early CT Score (PC-ASPECTS)**

| **Items** | **Scores** |
| --- | --- |
| any part of midbrain | 2 |
| any part of pons | 2 |
| left thalamus | 1 |
| right thalamus | 1 |
| left cerebellum | 1 |
| right cerebellum | 1 |
| left posterior cerebral artery territory | 1 |
| right posterior cerebral artery territory | 1 |
| **Total** |  |

From 10 points, 1 or 2 points each (as indicated) are subtracted for early ischemic changes in above items. PC-ASPECTS 10 indicates a normal scan. PC-ASPECTS 0 indicates extensive early ischemic changes.

**4.6 Pons-midbrain-index(PMI)**

| **Items** | **Scores** |
| --- | --- |
| left midbrain | 2 |
| right midbrain | 2 |
| left pons | 2 |
| right pons | 2 |
| **Total** |  |

A PMI of zero indicates no hypo-density in either the midbrain or the pons. Each side of both the midbrain and pons was classified as: 0 = no hypodensity;1 = 50% or less hypodensity; 2 = more than 50% hypodensity. A PMI of eight indicated bilateral hypodensity of more than 50% in both the midbrain and the pons.

**4.7 Modified Treatment in Cerebral Ischemia (mTICI) scale**

| **Grades** | **Definitions** |
| --- | --- |
| 0 | no perfusion |
| 1 | antegrade reperfusion past the initial occlusion, but limited distal branch filling with little or slow distal reperfusion |
| 2a | antegrade reperfusion of less than half of the occluded target artery previously ischemic territory |
| 2b | antegrade reperfusion of more than half of the previously occluded target artery ischemic territory |
| 3 | complete antegrade reperfusion of the previously occluded target artery ischemic territory, with absence of visualized occlusion in all distal branches |

# **5. References:**

[1].	Jovin, T.G., et al., Trial of Thrombectomy 6 to 24 Hours after Stroke Due to Basilar-Artery Occlusion. N Engl J Med, 2022. 387(15): p. 1373-1384.

[2].	Tao, C., et al., Trial of Endovascular Treatment of Acute Basilar-Artery Occlusion. N Engl J Med, 2022. 387(15): p. 1361-1372.

[3].	Adusumilli, G., et al., Endovascular thrombectomy after acute ischemic stroke of the basilar artery: a meta-analysis of four randomized controlled trials. J Neurointerv Surg, 2023. 15(e3): p. e446-e451.

[4].	Pop, R., et al., Poor clinical outcome despite successful basilar occlusion recanalization in the early time window: incidence and predictors. J Neurointerv Surg, 2023. 15(5): p. 415-421.

[5].	Hussein, H.M., et al., Occurrence and predictors of futile recanalization following endovascular treatment among patients with acute ischemic stroke: a multicenter study. AJNR Am J Neuroradiol, 2010. 31(3): p. 454-8.

[6].	Drieu, A., et al., Immune Responses and Anti-inflammatory Strategies in a Clinically Relevant Model of Thromboembolic Ischemic Stroke with Reperfusion. Transl Stroke Res, 2020. 11(3): p. 481-495.

[7].	Naderi, Y., et al., Neuroprotective effects of minocycline on focal cerebral ischemia injury: a systematic review. Neural Regen Res, 2020. 15(5): p. 773-782.

[8].	Lampl, Y., et al., Minocycline treatment in acute stroke: an open-label, evaluator-blinded study. Neurology, 2007. 69(14): p. 1404-10.

[9].	Switzer, J.A., et al., Matrix metalloproteinase-9 in an exploratory trial of intravenous minocycline for acute ischemic stroke. Stroke, 2011. 42(9): p. 2633-5.

[10].	Kohler, E., et al., Intravenous minocycline in acute stroke: a randomized, controlled pilot study and meta-analysis. Stroke, 2013. 44(9): p. 2493-9.

[11].	Baik, S.H., et al., Futile recanalization in patients with basilar artery occlusion: assessment of the underlying etiology and the role of perfusion imaging. J Neurointerv Surg, 2024.

[12].	Fu, Y., et al., Fingolimod for the treatment of intracerebral hemorrhage: a 2-arm proof-of-concept study. JAMA Neurol, 2014. 71(9): p. 1092-101.

[13].	Tian, D.C., et al., Fingolimod enhances the efficacy of delayed alteplase administration in acute ischemic stroke by promoting anterograde reperfusion and retrograde collateral flow. Ann Neurol, 2018. 84(5): p. 717-728.

[14].	Nighoghossian, N., et al., Cyclosporine in acute ischemic stroke. Neurology, 2015. 84(22): p. 2216-23.

[15].	Elkins, J., et al., Safety and efficacy of natalizumab in patients with acute ischaemic stroke (ACTION): a randomised, placebo-controlled, double-blind phase 2 trial. Lancet Neurol, 2017. 16(3): p. 217-226.

[16].	Sandercock, P.A. and T. Soane, Corticosteroids for acute ischaemic stroke. Cochrane Database Syst Rev, 2011. 2011(9): p. CD000064.

[17].	Strickland, B.A., et al., Neuroprotective effect of minocycline against acute brain injury in clinical practice: A systematic review. J Clin Neurosci, 2021. 86: p. 50-57.

[18].	Mulder, I.A., et al., Adjunctive cytoprotective therapies in acute ischemic stroke: a systematic review. Fluids Barriers CNS, 2021. 18(1): p. 46.

[19].	Zhang, M., et al., Ischemia-reperfusion injury: molecular mechanisms and therapeutic targets. Signal Transduct Target Ther, 2024. 9(1): p. 12.

**TRAIL PROTOCOL**

**Minocycline for acute basilar artery occlusion stroke undergoing endovascular treatment: a randomized, evaluator-blinded, open-label, pilot trial**

**12th February 2025**

**Version 3.0**

**TABLE OF CONTENTS**

[**1. Abbreviations and Definitions of Terms	1**](#_Toc207138866)

[**2. Trail Summary	2**](#_Toc207138867)

[**3. Background and Rationale	9**](#_Toc207138868)

[**4. Trail Methods	12**](#_Toc207138869)

[**5. Other information	24**](#_Toc207138870)

[**6. Appendix	27**](#_Toc207138871)

[**7. References:	39**](#_Toc207138872)

| **Abbreviations and Definitions of Terms** | |
| --- | --- |
| **EVT** | Endovascular treatment |
| **BAO** | Basilar artery occlusion |
| **e-NIHSS** | expanded National Institutes of Health Stroke Scale |
| **sICH** | symptomatic Intracranial Hemorrhage |
| **mRS** | modified Rankin Scale |
| **NIHSS** | National Institutes of Health Stroke Scale |
| **mBI** | modified Barthel index |
| **ICU** | Intensive Care Unit |
| **VA** | vertebral arteries |
| **CTA** | Computed tomography angiography |
| **MRA** | magnetic resonance angiography |
| **pc-ASPECTS** | posterior circulation Alberta Stroke Program Early CT score |
| **DWI** | diffusion-weighted imaging |
| **NCCT** | non-contrast cranial computed tomography |
| **PMI** | Pons-midbrain-index |
| **CIs** | confidence intervals |
| **RCT** | randomized controlled trial |
| **FR** | futile recanalization |
| **AIS** | acute ischemic stroke |
| **ITT** | Intention-to-Treat |
| **PP** | Per-Protocol |

**Trail Summary**

| **Protocol title** | Effects of Minocycline on Patients with Acute Ischemic Stroke Undergoing Endovascular Treatment due to Basilar Artery Occlusion (MIST-B): the study protocol for a multicenter, prospective, randomized, open-label clinical trial with blinded outcome assessment. |
| --- | --- |
| **Short Title** | MIST-B |
| **Protocol version** | Version 3.0, 12.02.2025 |
| **Trial registration** | Clinicaltrials.gov (NCT05512910) |
| **Background and Objective** | Successful recanalization after endovascular treatment (EVT) has been achieved in 85% of the patients with acute basilar artery occlusion (BAO), while less than half of them had good functional outcomes. Minocycline is a potential cytoprotectant with synergistic combination of antioxidant, anti-inflammatory, anti-apoptotic and protection of blood-brain barrier. Our trial aims to evaluate the safety and efficacy of adjunctive oral minocycline to EVT for patients with acute BAO. |
| **Study design** | Multi-center, evaluator-blinded, randomized (ratio 1:1), open-label, proof of concept trial. |
| **Outcomes** | The primary effectiveness outcome is expanded National Institutes of Health Stroke Scale (e-NIHSS) scores at 5 days or discharge.  The primary safety outcome is the incidence of symptomatic intracranial hemorrhage (sICH) at 24 hours.  Secondary efficacy outcomes include:  1.Ordinal Shift analysis of modified Rankin Scale (mRS) toward a better functional outcome at 90 days.  2.Dichotomized mRS scores (0-3 vs. 4-6; 0-2 vs. 3-6; 0-1 vs. 2-6) at 90(-14/+14) days from randomization.  3. National Institutes of Health Stroke Scale (NIHSS) score at baseline, 24 hours, 5 days or discharge, 30(-7/+7) days and 90(-14/+14) days from randomization.  4.e-NIHSS at baseline, 24 hours, 30(-7/+7) days and 90(-14/+14) days from randomization.  5. modified Barthel index (mBI) scores at baseline, 30(-7/+7) days and 90(-14/+14) days from randomization.  6.Incidence of sICH at 3 days from randomization.  7.Mortality at 90(-14/+14) days from randomization.  8.Changes in infarct volume from baseline to 5 days or discharge.  9.Intensive care outcomes are the following: (1) Length of Intensive Care Unit (ICU) stay and hospital stay; (2) Time of mechanical ventilation or non-invasive ventilation at 5 days from randomization or discharge; (3) Pneumonia at 5 days from randomization or discharge.  10.Lab hematology outcomes are the following: Change in percentage of the lymphocyte subpopulations, matrix metalloproteinase-9, IL-6, IL-10, TNF-α, leucocytes, percentage of neutrophilic granulocyte and absolute neutrophil value at 5 days or discharge as compared to baseline. |
| **Inclusion /**  **Exclusion criteria** | Inclusion criteria:  1) Age 18 years or older.  2) Patients had acute symptoms and signs compatible with acute ischemia stroke due to BAO or intracranial segments of vertebral arteries (VA) resulting in no flow to the basilar artery (eg, functional BAO).  3) Last known well to groin puncture between 0 to 24 hours, whether or not patients had thrombolysis with rt-PA.  4) Pre-stroke mRS score of 0-1.  5) Baseline e-NIHSS score ≥ 6.  6) Neuroimaging Inclusion Criteria: (1) Proven large vessel occlusion in BAO or VA-V4 occlusion (modified Thrombolysis In Cerebral Infarction [mTICI] score 0-1) determined by Computed tomography angiography (CTA) /magnetic resonance angiography (MRA); (2) posterior circulation Alberta Stroke Program Early CT score (pc-ASPECTS) ≥ 5 determined by diffusion-weighted imaging (DWI) or non-contrast cranial computed tomography (NCCT); (3) Pons-midbrain-index (PMI) of < 3.  7) Signed Informed Consent obtained.  Exclusion criteria:  1) Age < 18 years old.  2) Complete cerebellar infarct with significant mass effect or has the imaging features of acute hydrocephalus in NCCT.  3) Intracranial hemorrhage at baseline assessment.  4) Previous stroke in the past 90 days.  5) Cardiopulmonary resuscitation was performed within 10 days prior to onset.  6) Known hereditary or acquired hemorrhagic diathesis, coagulation factor deficiency, international normalized ratio > 3, or platelet < 40×10^9^/L.  7) Glucose < 2.2 mmol/L or > 22 mmol/L.  8) Systolic blood pressure persistently >185mmHg or diastolic blood pressure persistently >110mmHg despite antihypertensive intervention.  9) Acute or chronic renal failure of CKD grade 3-4.  10) Known allergy or hypersensitivity to contrast dye or tetracycline group of drugs.  11) Epileptic seizure at symptom onset.  12) Life expectancy (except for stroke) < 3 months.  13) Female who is pregnancy or breastfeeding, or whom do not use effective contraception at childbearing age.  14) Pre-existing mental illness that interferes with neurological evaluation.  15) Known current participation in another clinical investigation with experimental drug.  16) Unlikely to be available for 90 days follow-up. |
| **Measurements and procedures** | Patients presenting with posterior circulation acute ischemic stroke (AIS) due to occlusion in BAO or intracranial segments of VAs are enrolled. Randomization will be performed within 24 hours from symptom onset or last known well in patients meeting all in- and exclusion criteria. Ninety patients will be randomized (1:1) to either EVT only or oral minocycline adjunctively to EVT (45 per group).  All these participants will be followed up for 3 months. All patients undergo assessment of the e-NIHSS, NIHSS and mRS at baseline, 24 hours, 5 days or discharge, 30(-7/+7) days and 90(-14/+14) days from randomization. All patients undergo assessment of the mBI at baseline, 30(-7/+7) days and 90(-14/+14) days from randomization. At 5 days or discharge, MRI is repeated to determine changes in infarct volume. A treatment-blinded person will assess the outcomes dependency. |
| **Intervention** | Both groups will receive endovascular thrombectomy and standard medical.  Patients allocated to the adjunctive minocycline group will receive oral minocycline in addition to endovascular treatment and other standard medical. After randomization, oral minocycline should be given as soon as possible before the EVT treatment. The first dose of minocycline will be administered 200 mg orally, followed by 100 mg every 12 hours times for a total of 5 days. |
| **Trial Duration** | 30 months |
| **Trial Schedule** | Submission to Ethical Committee in China: 07/2022  03/2023: First-Patient-In (actual)  05/2025: Last-Patient-In (planned) |
| **Statistical Consideration** | The primary analysis will be performed in the intention-to-treat population. Differences in outcomes between the two groups will be analyzed using generalized linear models, modified poissson regression models or multivariable ordinal logistic regression as appropriate. The outcomes will be adjusted for age, sex, time from onset to random assignment, PMI index, and baseline e-NIHSS score. The risk ratio, the common odds ratio or mean differences and 95% confidence intervals (CIs) of coefficients in the model will be presented to describe differences between the two groups. |
| **Sample size** | This study will enroll 90 patients with acute ischemic stroke due to BAO who are eligible for EVT, providing 80% statistical power to reject the null hypothesis that, adjunctive with oral minocycline, the EVT has an at least 3 points lower in e-NIHSS scores at 5 days or discharge than receiving EVT alone. |
| **GCP Statement** | The study adheres to the latest principles of the Declaration of Helsinki and follows the Good Clinical Practice guidelines. |

# **1. Background and Rationale**

Two recent randomized controlled trials (RCTs) in posterior circulation demonstrated that endovascular treatment (EVT) confers significant benefits over medical therapy alone in patients with acute basilar artery occlusion (BAO)^[1, 2]^. However, a high proportion of patients (55%) continue to experience unfavorable functional outcomes (modified Rankin Scale [mRS] 4-5) or had died by the 90-day follow-up period) in a meta-analysis of the four recent posterior circulation trials^[3]^. The high incidence of futile recanalization (FR) in the posterior circulation might be highly associated with the poor prognosis after patients with acute BAO received EVT^[4]^.

The exact pathophysiological mechanisms underlying FR remain uncertain, but they may involve several factors, including the no-reflow phenomenon, early arterial re-occlusion, inadequate collateral circulation, hemorrhagic transformation, impaired cerebrovascular autoregulation, and a larger area of hypoperfusion^[5]^. Minocycline has been previously demonstrated individually to reduce infarction in animal models of stroke, and have potentially mechanisms of antioxidant, anti-inflammatory, anti-apoptotic and protection of blood-brain barrier^[6, 7]^. There is still no consensus whether minocycline have a clear advantage in neurologic outcomes because data have been limited to small randomized trials in acute ischemic stroke (AIS) without EVT^[8-10]^. Considering the effective in immediate tissue reperfusion of thrombectomy techniques, the adjunctive minocycline with EVT might be a viable pathway to improve neurological deficits early after stroke in patients with AIS due to BAO.

**1.1 Research question and research in context**

The purpose of current study is to evaluate the effects of adjunctive oral minocycline in patients with AIS due to BAO in the setting of receiving endovascular treatment.

**1.2 Clinical evidence to date**

*Limitations of EVT on large-vessel occlusions*

Futile recanalization is a crucial factor that impaired efficacy of EVT. A better understanding of the predictors of FR, such as disease severity at baseline, reperfusion delay, incomplete recanalization, may provide important insights into management of the disease^[11]^. The pathological mechanism of FR is still unclear, but reperfusion injury and its subsequent inflammatory are involved. It may be worth exploring the use of a cytoprotectant as a viable intervention to mitigate subsequent injury caused by ischaemia and reperfusion.

*Evidences of anti-inflammatory therapy on large-vessel occlusions with EVT*

Preclinical models suggested potential benefits of methylprednisolone, fingolimod, natalizumab, and cyclosporine in stroke treatment, yet none have been validated by clinical trials^[12-16]^. Recently randomized clinical trial, MARVEL, aimed to assess the efficacy and adverse events of adjunctive intravenous low-dose methylprednisolone to endovascular thrombectomy for acute ischemic stroke secondary to large-vessel occlusion^[17]^. However, the MARVEL trial did not show significant benefits of adjunctive intravenous low-dose methylprednisolone on overall disability. The secondary outcome finding that low-dose methylprednisolone was associated with lower mortality, lower rate of symptomatic intracranial hemorrhage and less pneumonia. The trail has provided evidence regarding the potential effect of anti-inflammatory therapy in the setting of endovascular stroke reperfusion.

*Evidences of minocycline on AIS*

Minocycline is a second-generation tetracycline capable of inhibiting bacterial protein synthesis with high blood-brain barrier permeability. It affects numerous molecular pathways that have been implicated in neurologic injury, including abilities to inhibit microglial activation, reduce T-cell migration, scavenge free radicals, decrease chemokine expression, and inhibit matrix metalloproteases, minimizing degradation of the basal lamina and stabilizing blood brain barrier integrity^[18]^. Animal model studies and several researches showed its effect on reducing infarction of stroke, while the evidence was limited due to sample size and trail design. MIST-B distinguishes itself from previously researches primarily through the application of minocycline on acute stroke patients receiving endovascular treatment. Considering the effective in immediate tissue reperfusion of thrombectomy techniques, the adjunctive minocycline with EVT might be a viable pathway to reduce FR.

*Interpretation of existing evidence*

Effective delivery to ischemic tissue is a key determinant of minocycline's potential utility following EVT, where successful recanalization may facilitate drug access^[19]^. Given the compelling biological rationale linking minocycline's mechanisms to ischemia/reperfusion injuries, rigorous evaluation as an adjunctive therapy to EVT is warranted^[20]^. In summary, there is clinical equipoise regarding whether minocycline in addition to EVT has a positive treatment effect in AIS patients with a BAO and we therefore propose a multi-center, evaluator-blinded, randomized, open-label, comparison of oral minocycline plus EVT to EVT alone with the expanded National Institutes of Health Stroke Scale (e-NIHSS) scores at 5 days or discharge as the main outcome parameter.

# **2. Trail Methods**

**2.1 Design**

Minocycline for acute Ischemic Stroke undergoing endovascular Treatment due to Basilar artery occlusion (MIST-B) is a multi-center, evaluator-blinded, randomized, open-label, proof of concept trial. The aim of the MIST-B is to assess the safety and efficacy of adjunctive oral minocycline to EVT for patients with acute BAO. The study has a planned duration of 2.5 years to recruit 90 patients across 12 comprehensive stroke centers in China. The study patient flow outline is shown in **Figure 1**. All enrolled patients will be randomized with 1:1 to EVT only or oral minocycline adjunctively to EVT within 24 hours of symptom onset and receive continuous application for 5 days. All these participants will receive standard medical care afterwards and be followed up for 3 months. The study adheres to the latest principles of the Declaration of Helsinki and follows the Good Clinical Practice guidelines. The MIST-B trail (www.clinicaltrials.gov. NCT05512910) has received approval from The Ethical Committee of Xijing Hospital (XJLL-KY2022184).

Figure 1. MIST-B flow chart.

**2.2 Patient population**

Patients presenting with posterior circulation AIS due to occlusion in BAO or intracranial segments of vertebral arteries (VAs) are enrolled. Patients will be randomized within 24 hours from symptom onset or last known well.

Inclusion criteria:

1) Age 18 years or older.

2) Patients had acute symptoms and signs compatible with acute ischemia stroke due to BAO or intracranial segments of VAs resulting in no flow to the basilar artery (eg, functional BAO).

3) Last known well to groin puncture between 0 to 24 hours, whether or not patients had thrombolysis with rt-PA.

4) Pre-stroke mRS score of 0-1.

5) Baseline e-NIHSS score ≥ 6.

6) Neuroimaging Inclusion Criteria: (1) Proven large vessel occlusion in BAO or VA-V4 occlusion (mTICI score 0-1) determined by Computed tomography angiography (CTA) /magnetic resonance angiography (MRA); (2) posterior circulation Alberta Stroke Program Early CT score (pc-ASPECTS) ≥ 5 determined by diffusion-weighted imaging (DWI) or non-contrast cranial computed tomography (NCCT); (3) Pons-midbrain-index (PMI) of < 3.

7) Signed Informed Consent obtained.

Exclusion criteria:

1) Age < 18 years old.

2) Complete cerebellar infarct with significant mass effect or has the imaging features of acute hydrocephalus in NCCT.

3) Intracranial hemorrhage at baseline assessment.

4) Previous stroke in the past 90 days.

5) Cardiopulmonary resuscitation was performed within 10 days prior to onset.

6) Known hereditary or acquired hemorrhagic diathesis, coagulation factor deficiency, international normalized ratio > 3, or platelet < 40×10^9^/L.

7) Glucose < 2.2 mmol/L or > 22 mmol/L.

8) Systolic blood pressure persistently >185mmHg or diastolic blood pressure persistently >110mmHg despite antihypertensive intervention.

9) Acute or chronic renal failure of CKD grade 3-4.

10) Known allergy or hypersensitivity to contrast dye or tetracycline group of drugs.

11) Epileptic seizure at symptom onset.

12) Life expectancy (except for stroke) < 3 months.

13) Female who is pregnancy or breastfeeding, or whom do not use effective contraception at childbearing age.

14) Pre-existing mental illness that interferes with neurological evaluation.

15) Known current participation in another clinical investigation with experimental drug.

16) Unlikely to be available for 90 days follow-up.

**2.3 Randomization**

Central randomization system will be used to randomize subjects. After occlusion of basilar artery has been confirmed and written informed consent has been obtained, patients will be immediately randomly assigned to adjunctive minocycline group or control group in a 1:1 ratio. Patients randomly assigned to Minocycline group were prescribed minocycline capsules as soon as possible after randomization, ideally before the EVT or no more than 1 hour after EVT. Randomization will not be stratified by participating centre or other parameters.

The randomization sequence will be generated using SAS V.9.4 software. The randomization procedure is web based and runs on mobile devices or web page platforms(http://iwrs.xarsk.com/). The participating sites will be continuously and carefully monitored for crossovers and other protocol deviations. Researches had the access to the randomization results except the independent assessor at each trial site. It will only be sent to the data coordination centre responsible for central randomization and data management and the centre responsible for blinding emergency patients.

**2.4 Intervention**

Both groups will receive endovascular thrombectomy and standard medical. Patients allocated to the adjunctive minocycline group will receive oral minocycline in addition to endovascular treatment and other standard medical. After randomization, oral minocycline should be given as soon as possible before the EVT treatment. The first dose of minocycline will be administered 200 mg orally, followed by 100 mg every 12 hours times for a total of 5 days. If the patient is considered at risk for aspiration or unable to swallow based on a swallowing evaluation, minocycline will be administered via a feeding tube. Considering the risk of difficulty in feeding tube before EVT, minocycline administered within one hours after EVT is acceptable.

Documentation of Deviations: Investigators must adhere strictly to the study protocol. Deviation from the treatment regimen is defined as the subject not receiving any assigned treatment, unless the subject met the criteria for treatment discontinuation. All protocol deviations will be documented in a protocol deviation log, which will be reviewed regularly by the trial steering committee to ensure consistency in applying the exclusion criteria. The rationale for excluding each participant will be documented in detail.

**2.5 Study assessment and treatment masking**

All scale assessments will be performed by independent certified assessors blinded to treatment at each center. An independent assessor in charge of assessment of baseline data, in-hospital data and standardized visits will be blinded to treatment assignment and actual treatment in each centre. These assessments are recorded, and a comprehensive follow-up report is compiled. Centralized interpretation will be performed for all study-related clinical outcomes and imaging data. The accuracy of outcome events is finally assessed by Data and Safety Management Board (DSMB). The DSMB will be blinded to treatment allocation, drug use, and participant identity.

During the hospital stay, all concomitant treatments, adverse events, and any serious adverse events will be recorded. For patients discharged prior to 5-day visit, clinical assessments at discharge were recorded. At 30 days and 90 days from randomization, a face-to-face or telephone follow-up will be conducted by independent certified assessors. If all attempts to reach the patients are exhausted without success, they will be classified as lost to follow-up upon finalization of the case report form. The day-30 outcome was allowed a time window of ±7 days, and the day-90 outcome a window of ±14 days from the target date.

All patients undergo assessment of the e-NIHSS, National Institutes of Health Stroke Scale (NIHSS) and mRS at baseline, 24 hours, 5 days, 30 days and 90 days from randomization. All patients undergo assessment of the modified Barthel Index (mBI) at baseline, 30 days and 90 days from randomization. At 5 days, MRI is repeated to determine changes in infarct volume.

Plasma samples for MMP-9 evaluation were obtained at baseline (pre-minocycline) and at 5 days. Blood samples were placed on ice and then centrifuged at 3000 RPM for 5 minutes within 1 hours of collection. Plasma was removed, aliquoted and stored at –80℃ until analysis. The quantitative of MMP-9 will be assessed using Human MMP-9 ELISA Kit (abcam) according to the manufacturer instructions. Other laboratory hematology outcomes, such as lymphocyte subpopulations, IL-6, IL-10, TNF-α, leukocytes, neutrophil percentage, and absolute neutrophil count, will be assessed based on the capabilities of the laboratory departments at each participating center.

**2.6 Contents of Investigational Product Kit**

Minocycline Hydrochloride Capsules are manufactured by Hisun Pharmaceutical Group Co., Ltd., Hangzhou, China. All study medication will be provided by Xijing Hospital.

**2.7 End of Study and Length of Study**

The study will conclude on the date of the last patient's last visit. The conclusion is anticipated to take place 90 days post-randomization of the final participant. The entire study, from the initial patient screening to the last patient's last visit, is estimated to complete within 2.5 years.

**2.8 Primary outcomes**

The primary effectiveness outcome is e-NIHSS scores at 5 days. The e-NIHSS is a modified NIHSS focused on posterior circulation strokes, with the advantage to improve sensitivity of outcome assessment ^[11]^. Given the variability in clinical practice across centers, e-NIHSS assessment scheduled for day 5 was permitted up to day 7 after randomization. For patients discharged before day 5, the e-NIHSS score was recorded at discharge.

The primary safety outcome is the incidence of symptomatic intracranial hemorrhage (sICH) at 24 hours from randomization. sICH is defined as local or remote parenchymal hemorrhage type 2, subarachnoid hemorrhage, and/or intraventricular hemorrhage on the post-treatment imaging scan, combined with a neurological deterioration of 4 points or more on the NIHSS from baseline, or leading to death.

**2.9 Secondary outcomes**

1.Ordinal Shift analysis of mRS toward a better functional outcome at 90 days.

2.Dichotomized mRS scores (0-3 vs. 4-6; 0-2 vs. 3-6; 0-1 vs. 2-6) at 90 days from randomization.

3.NIHSS at baseline, 24 hours, 5 days, 30 days and 90 days from randomization.

4.e-NIHSS at baseline, 24 hours, 30 days and 90 days from randomization.

5.mBI scores at baseline, 30 days and 90 days from randomization.

6.Incidence of sICH at 3 days from randomization.

7.Mortality at 90 days from randomization.

8.Changes in infarct volume from baseline to 5 days.

9.Intensive care outcomes are the following: (1) Length of Intensive Care Unit (ICU) stay and hospital stay; (2) Time of mechanical ventilation or non-invasive ventilation at 5 days from randomization or discharge; (3) Pneumonia at 5 days from randomization.

10.Lab hematology outcomes are the following: Change in percentage of the lymphocyte subpopulations, matrix metalloproteinase-9, IL-6, IL-10, TNF-α, leucocytes, percentage of neutrophilic granulocyte and absolute neutrophil value at 5 days.

**2.10 Adverse events and Safety**

Laboratory assessments will be performed to monitor adverse events at baseline, 24 hours, 5 days, 30 days and 90 days from randomization. CT is repeated to determine intracranial hemorrhages at 24 hours after randomization, or anytime a neurological deterioration on the NIHSS. The abnormal laboratory results or intracranial hemorrhages in CT will be evaluated by the safety committee, and be assessed their relation to the minocycline. Minocycline should be withdrawn if white blood cell counts below 3.0 * 10^9/L, neutrophil counts below 1.5 * 10^9/L, platelet counts below 100 * 10^9/L, hemoglobin levels below 30g/L, or marked increases in bilirubin, aspartate aminotransferase, alanine aminotransferase, creatinine, or urea. All adverse events will be meticulously recorded, and serious adverse events will be promptly reported to both the safety and ethics committees within 24 hours. Participants who experience serious harm due to their involvement in the study will receive appropriate financial compensation.

**2.11 Data Quality and Monitoring**

Data Monitoring Committee will be established to uniform data quality across participating centers. The committee will prepare periodic reports on data integrity, protocol adherence, and patient outcomes. The Principal Investigator at each center will oversee quality control to ensure that the trial is conducted in accordance with the protocol. A data management plan will be implemented, and all information required for monitoring and analysis of the study will be stored securely in a locked filing cabinet within a restricted-access office. The digital records will be safeguarded on a computer that is secured with a password and a firewall. Study data will be regularly monitored for protocol compliance, data accuracy and completeness onsite by the sponsor or their delegates, in accordance with the study’s monitoring plan. The data controller for the study will be the trial's sponsor.

**2.12 Sample size estimates**

The primary hypothesis is that patients receiving adjunctive minocycline in addition to EVT within 24 hours after stroke onset, will have better neurological outcome at 5 days or discharge. We based our estimations on the results of the ATTENTION trial^[1]^. The means (SD) of NIHSS scores in patients with thrombectomy at 5 days or discharge is calculated as 16±5.3 by the methods of Iztok Hozo^[12]^. Due to the lack of previous data on adjunctive minocycline in addition to EVT, it is considered a reasonable prediction that NIHSS scores in adjunctive minocycline group would be 3 points lower than those in control group at 5 days or discharge, based on the discussion by the Academic Committee and the Executive Committee. The e-NHISS scores, the main endpoint of efficacy set in this study, could improve the sensitivity of NIHSS scores in posterior circulation stroke and could have an impact on clinical trials. We use the NIHSS parameters in previous studies to estimate the sample size of this study. Therefore, we calculated that a sample size of 90 patients (45 patients in each group with a dropout rate of 10%) would detect a 3 points difference (16±5.3 for the control group and 13±5.3 for the intervention group) in e-NIHSS scores of patients at 5 days or discharge, with a power of 80% and two-sided significance levels of 0.05 for the analysis of the primary effectiveness outcome. Sample size was computed using PASS 11.0.7.

**2.13 Statistical analyses**

Intention-to-Treat (ITT) population, Per-Protocol (PP) population and Safety population are planned for the studies. The primary analysis will be performed in the intention-to-treat population. Continuous variables will be reported as means (SD), and categorical variables will be presented as percentages. The Kolmogorov-Smirnoff test is used to assess the normality of the continuous variables. Differences in baseline characteristics between the two groups will be compared by either Student’s t test or Mann-Whitney U test for continuous variables and with the chi-square test for categorical variables. Differences in primary outcomes between the two groups will be analyzed using linear models. The mean differences and 95% confidence intervals (CIs) of coefficients in the model will be presented to describe differences between the two groups.

Missing values for baseline characteristics will be reported. Missing baseline characteristics will be imputed using regression imputation. All hypothesis tests will adopt a two-sided alpha level of significant set at 0.05 (α=0.05).

**2.14 Patient Involvement and Informed consent**

We recruit participants through Green Channel of Acute Ischemic Stroke and conduct trials with sufficient information on the study design. All participants will be thoroughly informed about the study's goals, the potential side effects associated with the procedures and interventions, who will have access to their personal information, and any compensation available for individuals who may experience harm as a result of participating in the trial. It will be clearly emphasized that participation is entirely voluntary and that participants have the right to withdraw from the study at any time without any influence on their subsequent care. There is one informed consent document required for MIST-B. Patients or the public involves in the design, or conduct, or reporting, or dissemination plans of our research.

**2.15 Ethics and Dissemination**

Central ethics approval was obtained from The Ethics Committee of Xijing Hospital, Xi’an, China (XJLL-KY2022184). The trail is registered at Clinicaltrials.gov (NCT05512910). Any modifications to the protocol which may impact the conduct of the study, potential benefit of the participant, or may affect participant safety, will require a formal amendment to the protocol. Such amendments will be agreed on by the study steering committee and approved by the ethics committee prior to implementation. A report describing the results of the study will be submitted to international meetings and peer-reviewed journals.

# **3 Other information**

**3.1 Trial site and Investigators**

**Chief Investigator:**

Dr. Wen Jiang, Department of Neurology, Xijing Hospital, Fourth Military Medical University;

**Co-investigators:**

Dr. Rong Yin, Department of Neurology, Gansu Province Central Hospital;

Dr. Huiping Zhang, Department of Neurology, Baoji High-tech Hospital;

Dr. Li Yao, Department of Neurology, Xi'an XD Group Hospital;

Dr. Weiwang Li, Department of Neurology, Xi'an Daxing Hospital;

Dr. Liping Yu, Department of Neurology, First People's Hospital of Xianyang;

Dr. Xiangjun Yuan, Department of Neurology, Weinan Central Hospital;

Dr. Yi Jia, Department of Neurology, Xi'an Gaoxin Hospital;

Dr. Bo Song, Department of Neurosurgery, Xianyang Hospital of Yan'an University;

Dr. Baiya Fan, Department of Neurology, Yan'an People's Hospital;

Dr. Xingshun Ma, Department of Neurology, The First Hospital of Yulin;

Dr. Xue Li, Department of Neurology, The Second Hospital of Yulin.

**3.2 Statisticians**

Pro. Duolao Wang, Department of Clinical Sciences, Liverpool School of Tropical Medicine;

Pro. Lei Shang and Pro. Ling Wang, Department of Health Statistics, Fourth Military Medical University;

Pro. Bohui Zhang, Department of Cardiology, Fourth Military Medical University.

**3.3 Academic Committee**

Dr. Wen Jiang and Dr. Dong Wei, Department of Neurology, Xijing Hospital, Fourth Military Medical University;

Pro. Lei Shang and Pro. Ling Wang, Department of Health Statistics, Fourth Military Medical University;

Dr. Hongzeng Li, Department of Neurology, Tangdu Hospital, Fourth Military Medical University;

Dr. Jianbo He, Department of Neurology, Xi'an XD Group Hospital;

Dr. Mingze Chang, Department of Neurology, Xi'an Third Hospital;

Dr. Yi Jia, Department of Neurology, Xi'an Gaoxin Hospital;

Dr. Rong Yin, Department of Neurology, Gansu Provincial Maternity and Child-care Hospital (Gansu Provincial Central Hospital);

Dr. Liping Yu, Department of Neurology, First People's Hospital of Xianyang.

**3.4 Executive Committee**

Dr. Wen Jiang, Dr. Dong Wei, Dr. Rui Shi, Dr. Deshuai Li, Dr. Zhirong Fan, Dr. Yang Li, Dr. Luojun Wang and Dr. Jingjing Zhao, Department of Neurology, Xijing Hospital, Fourth Military Medical University;

Pro. Lei Shang and Pro. Li Wang, Department of Health Statistics, Fourth Military Medical University;

Dr. Jianbo He and Dr. Li Yao, Department of Neurology, Xi'an XD Group Hospital;

Dr. Mingze Chang, Department of Neurology, Xi'an Third Hospital;

Dr. Yi Jia, Department of Neurology, Xi'an Gaoxin Hospital;

Dr. Rong Yin, Department of Neurology, Gansu Provincial Maternity and Child-care Hospital (Gansu Provincial Central Hospital);

Dr. Liping Yu, Department of Neurology, First People's Hospital of Xianyang.

**3.5 Independent assessors in each centre**

Dr. Le Wang, Department of Neurology, Xijing Hospital, Fourth Military Medical University;

Dr. Yaxuan Wei, Department of Neurology, Gansu Provincial Maternity and Child-care Hospital (Gansu Provincial Central Hospital);

Dr. Yanan Yang, Department of Neurology, Baoji High-tech Hospital;

Dr. Juanli Zhang, Department of Neurology, Xi'an XD Group Hospital;

Dr. Haixia Pei, Department of Neurology, Xi'an Daxing Hospital;

Dr. Hao Zhu, Department of Neurology, First People's Hospital of Xianyang;

Dr. Xingyun Yuan, Department of Neurology, First People's Hospital of Xianyang;

Dr. Huijuan Shang, Department of Neurology, Weinan Central Hospital;

Dr. Bao Guo, Department of Neurology, Weinan Central Hospital;

Dr. Saibing Liu, Department of Neurology, Xi'an Gaoxin Hospital;

Dr. Jian Miao, Department of Neurosurgery, Yan'an University Xianyang Hospital;

Dr. Hairong Gao, Department of Neurology, Yan 'an People's Hospital;

Dr. Hong Zhang, Department of Neurology, The First Hospital of Yulin;

Dr. Hufei Chang, Department of Neurology, Yulin Hospital, The First Affiliated Hospital of Xi'an Jiaotong University.

**3.6 Data and Safety Management Board (DSMB)**

Dr. Zhirong Liu, Department of Neurology, Xijing Hospital, Fourth Military Medical University;

Dr. Hongzeng Li, Department of Neurology, Tangdu Hospital, Fourth Military Medical University;

Pro. Lei Shang, Department of Health Statistics, Fourth Military Medical University.

# **4. Appendix**

**4.1 Expanded National Institutes of Health Stroke Scale (e-NIHSS)**

The e-NIHSS is a modified NIHSS focused on posterior circulation strokes, with the advantage to improve sensitivity of outcome assessment. The e-NIHSS consists of 13 items with scores from 0 to 42, with 5 categories added on the existing items 4, 6, and 9(showed as the bolded words in below scale).

| Instructions | Scale definition |
| --- | --- |
| 1a. Level of consciousness. The investigator must choose a response if a full evaluation is prevented by such obstacles as an endotracheal tube, language barrier, orotracheal trauma/bandages. A 3 is scored only if the patient makes no movement (other than reflexive posturing) in response to noxious stimulation. | 0=Alert; keenly responsive.  1=Not alert; but arousable by minor stimulation to obey, answer, or respond.  2=Not alert; required repeated stimulation to attend, or is obtunded and requires strong or painful stimulation to make movements (not stereotyped).  3=Responds only with reflex motor or autonomic effects or totally unresponsive, flaccid and areflexic. |
| 1b. LOC Questions: The patient is asked the month and his/her age. The answer must be correct – there is not partial credit for being close. Phasic and stuporous patients who do not comprehend the questions will score 2. Patients unable to speak because of endotracheal intubation, orotracheal trauma, severe dysarthria from any cause, language barrier, or any other problem not secondary to aphasia are given a 1. It is important that only the initial answer be graded and that the examiners not “help” the patient with verbal or nonverbal clues. | 0=Answers both questions correctly.  1=Answers one question correctly.  2=Answers neither question correctly. |
| 1c. LOC Commands: The patient is asked to open and close the eyes and then to grip and release the non-paretic hand. Substitute another one step command if the hand cannot be used. Credit is given if an unequivocal attempt is made but not completed due to weakness. If the patient does not respond to command, the task should be demonstrated to him or her (pantomime), and the result scored (i.e. follows none, one or two commands). Patients with trauma, amputation, or other physical impediments should be given suitable one-step commands. Only the first attempt is scored. | 0=Performs both tasks correctly.  1=Performs one task correctly.  2=Performs neither task correctly. |
| 2. Best Gaze: Only horizontal eye movements will be tested. Voluntary or reflexive (oculocephalic) eye movements will be scored, but caloric testing is not done. If the patient has a conjugate deviation of the eyes that can be overcome by voluntary or reflexive activity, the score will be a 1. If a patient has an isolated peripheral nerve paresis (CN III, IV or VI), score a 1. Gaze is testable in all aphasic patients. Patients with ocular trauma, bandages, preexisting blindness, or other disorder of visual acuity or fields should be tested with reflexive movements, and a choice made by the investigator. Establishing eye contact and then moving about the patient from side to side will occasionally clarify the presence of a partial gaze palsy. | 0= Normal.  1= Partial gaze palsy; gaze is abnormal in one or both eyes, but forced deviation or total gaze paresis is not present.  **1 = nystagmus and/or Horner’s syndrome**  2= Forced deviation; or total gaze paresis not overcome by the oculocephalic maneuver. |
| 3. Visual: Visual fields (upper and lower quadrants) are tested by confrontation, using finger counting or visual threat, as appropriate. Patients may be encouraged, but if they look at the side of the moving finger appropriately, this can be scored as normal. If there is unilateral blindness or enucleation, visual fields in the remaining eye are scored. Score 1 only if a clearcut asymmetry, including quadrantanopia, is found. If patient is blind from any cause, score 3. Double simultaneous stimulation is performed in this case. If there is extinction, the patient receives a 1, and the results are used to respond to item 11. | 0=No visual loss.  1= Partial hemianopia.  2= Complete hemianopia.  3= Bilateral hemianopia (blind including cortical blindness) |
| 4. Facial palsy: Ask or use pantomime to encourage the patient to show teeth or raise eyebrows and close eyes. Score symmetry of grimace in response to noxious stimuli in the poorly response or non-comprehending patient. If facial trauma/bandages, orotracheal tube, tape or other physical barriers obscure the face, these should be removed to the extent possible. | 0 = Normal symmetrical movements.  1= Minor paralysis (flattened nasolabial fold, asymmetry on smiling)  2= Partial paralysis (total or near-total paralysis of lower face)  3= Complete paralysis of one or both sides (absence of facial movement in the upper and lower face).  **3 = deficit of IX nerve (soft palate paralysis)**  **3 = deficit of XII nerve** |
| 5. Motor arm: The limb is placed in the appropriate position: extend the arms (palms down) 90 degrees (if sitting) or 45 degrees (if supine). Drift is scored if the arm falls before 10 seconds. The aphasic patient is encouraged using urgency in the voice and pantomime, but not noxious stimulation. Each limb is tested in turn, beginning with the non-paretic arm. Only in the case of amputation or joint fusion at the shoulder, the examiner should record the score as untestable (UN), and clearly write the explanation for this choice. | 0= No drift; limb holds 90 (or 45) degrees for full 10 seconds.  1= Drift; limb holds 90 (or 45) degrees, but drifts down before full 10 seconds; does not hit bed or other support.  2= Some effort against gravity; limb cannot get to or maintain (if cued) 90 (or 45) degrees, drifts down to bed, but has some effort against gravity.  3= No effort against gravity; limb falls.  4= No movement.  UN = Amputation or joint fusion: explain:  5a = Left Arm.  5b = Right arm. |
| 6. Motor leg: The limb is placed in the appropriate position: hold the leg at 30 degrees (always tested supine). Drift is scored if the leg falls before 5 seconds. The aphasic patient is encouraged using urgency in the voice and pantomime, but not noxious stimulation. Each limb is tested in turn, beginning with the non-paretic leg. Only in the case of amputation or joint fusion at the hip, the examiner should record the score as untestable (UN), and clearly write the explanation for this  choice. | 0= No drift; leg holds 30-degree position for full 5seconds.  1= Drift; leg falls by the end of the 5-second period but does not hit bed.  2= Some effort against gravity; leg falls to bed by 5 seconds, but has some effort against gravity.  3= No effort against gravity; leg falls to bed immediately.  4= No movement.  UN = Amputation or joint fusion: explain:  6a. Left Leg  6b. Right Leg. |
| 7. Limb ataxia: This item is aimed at finding evidence of a unilateral cerebellar lesion. Test with eyes open. In case of visual defect, ensure testing is done in intact visual field. The finger-nose-finger and heel-shin tests are performed on both sides, and ataxia is scored only if present out of proportion to weakness. Ataxia is absent in the patient who cannot understand or is paralyzed. Only in the case of amputation or joint fusion, the examiner should record the score as untestable (UN), and clearly write the explanation for this choice. In case of blindness, test by having the patient touch nose from extended arm position. | 0=Absent.  1= Present in one limb.  **1 = imbalance in Romberg position.**  2= Present in two limbs.  **2 = trunk ataxia or retro- or lateropulsion.**  UN = Amputation or joint fusion: explain: |
| 8. Sensory: Sensation or grimace to pinprick when tested, or withdrawal from noxious stimulus in the obtunded or aphasic patient. Only sensory loss attributed to stroke is scored as abnormal and the examiner should test as many body areas (arms [not hands], legs, trunk, face) as needed to accurately check for hemisensory loss. A score of 2, 'severe or total sensory loss', should only be given when a severe or total loss of sensation can be clearly demonstrated. Stuporous and aphasic patients will, therefore, probably score 1 or 0. The patient with brainstem stroke who has bilateral loss of sensation is scored 2. If the patient does not respond and is quadriplegic, score 2. Patients in a coma (item 1a=3) are automatically given a 2 on this item. | 0= Normal; no sensory loss.  1= Mild-to-moderate sensory loss; patients feels pinprick is less sharp or is dull on the affected side; or there is a loss of superficial pain with pinprick, but patient is aware of being touched.  2= Severe to total sensory loss; patient is not aware of being touched in the face, arm and leg. |
| 9. Best language: A great deal of information about comprehension will be obtained during the preceding sections of the examination. For this scale item, the patient is asked to describe what is happening in the attached picture, to name the items on the attached naming sheet and to read from the attached list of sentences. Comprehension is judged from responses here, as well as to all of the commands in the preceding general neurological exam. If visual loss interferes with the tests, ask the patient to identify objects placed in the hand, repeat, and produce speech. The intubated patient should be asked to write. The patient in a coma (item 1a=3) will automatically score 3 on this item. The examiner must choose a score for the patient with stupor or limited cooperation, but a score of 3 should be used only if the patient is mute and follows no one-step commands. | 0= No aphasia; normal  1= Mild-to-moderate aphasia; some obvious loss of fluency or facility of comprehension, without significant limitation on ideas expressed or form of expression. Reduction of speech and/or comprehension, however, makes conservation about provided materials difficult or impossible. For example, in conversation about provided materials, examiner can identify picture or naming card content from patient's response.  2= Severe aphasia; all communication is through fragmentary expression; great need for inference, questioning, and guessing by the listener. Range of information that can be exchanged is limited; listener carries burden of communication. Examiner cannot identify materials provided from patient response.  3= Mute, global aphasia: no usable speech or auditory comprehension. |
| 10. Dysarthria: If patient is thought to be normal, an adequate sample of speech must be obtained by asking patient to read or repeat words from the attached list. If the patient has severe aphasia, the clarity of articulation of spontaneous speech can be rated. Only if patient is intubated or has other physical barriers to producing speech, the examiner should record the score as untestable (UN), and clearly write an explanation for this choice. Do not tell the patient why he or she is being tested. | 0= Normal.  1= Mild-to-moderate dysarthria; patient slurs at least some words and, at worst, can be understood by some difficulty.  2= Severe dysarthria: patient's speech is so slurred as to be unintelligible in the absence of or out of proportion to any dysphasia, or is mute/anarthric.  UN = Intubated or other physical barrier. |
| 11. Extinction and Inattention (formerly Neglect): Sufficient information to identify neglect may be obtained during the prior testing. If the patient has a severe visual loss preventing visual double simultaneous stimulation, and the cutaneous stimuli are normal, the score is normal. If the patient has aphasia but does appear to attend to both sides, the score is normal. The presence of visual spatial neglect or anosagnosia may also be taken as evidence of abnormality. Since the abnormality is scored only if present, the item is never untestable. | 0= No abnormality.  1= Visual, tactile, auditory, spatial, or personal inattention or extinction to bilateral simultaneous stimulation in one of the sensory modalities.  2= Profound hemi-inattention or extinction to more than one modality; does not recognize own hand or orients to only one side of space. |

**4.2 National Institutes of Health Stroke Scale (NIHSS)**

| **Instructions** | **Scale definition** |
| --- | --- |
| 1a. Level of consciousness. The investigator must choose a response if a full evaluation is prevented by such obstacles as an endotracheal tube, language barrier, orotracheal trauma/bandages. A 3 is scored only if the patient makes no movement (other than reflexive posturing) in response to noxious stimulation. | 0=Alert; keenly responsive.  1=Not alert; but arousable by minor stimulation to obey, answer, or respond.  2=Not alert; required repeated stimulation to attend, or is obtunded and requires strong or painful stimulation to make movements (not stereotyped).  3=Responds only with reflex motor or autonomic effects or totally unresponsive, flaccid and areflexic. |
| 1b. LOC Questions: The patient is asked the month and his/her age. The answer must be correct – there is not partial credit for being close. Phasic and stuporous patients who do not comprehend the questions will score 2. Patients unable to speak because of endotracheal intubation, orotracheal trauma, severe dysarthria from any cause, language barrier, or any other problem not secondary to aphasia are given a 1. It is important that only the initial answer be graded and that the examiners not “help” the patient with verbal or nonverbal clues. | 0=Answers both questions correctly.  1=Answers one question correctly.  2=Answers neither question correctly. |
| 1c. LOC Commands: The patient is asked to open and close the eyes and then to grip and release the non-paretic hand. Substitute another one step command if the hand cannot be used. Credit is given if an unequivocal attempt is made but not completed due to weakness. If the patient does not respond to command, the task should be demonstrated to him or her (pantomime), and the result scored (i.e. follows none, one or two commands). Patients with trauma, amputation, or other physical impediments should be given suitable one-step commands. Only the first attempt is scored. | 0=Performs both tasks correctly.  1=Performs one task correctly.  2=Performs neither task correctly. |
| 2. Best Gaze: Only horizontal eye movements will be tested. Voluntary or reflexive (oculocephalic) eye movements will be scored, but caloric testing is not done. If the patient has a conjugate deviation of the eyes that can be overcome by voluntary or reflexive activity, the score will be a 1. If a patient has an isolated peripheral nerve paresis (CN III, IV or VI), score a 1. Gaze is testable in all aphasic patients. Patients with ocular trauma, bandages, preexisting blindness, or other disorder of visual acuity or fields should be tested with reflexive movements, and a choice made by the investigator. Establishing eye contact and then moving about the patient from side to side will occasionally clarify the presence of a partial gaze palsy. | 0= Normal.  1= Partial gaze palsy; gaze is abnormal in one or both eyes, but forced deviation or total gaze paresis is not present.  2= Forced deviation; or total gaze paresis not overcome by the oculocephalic maneuver. |
| 3. Visual: Visual fields (upper and lower quadrants) are tested by confrontation, using finger counting or visual threat, as appropriate. Patients may be encouraged, but if they look at the side of the moving finger appropriately, this can be scored as normal. If there is unilateral blindness or enucleation, visual fields in the remaining eye are scored. Score 1 only if a clearcut asymmetry, including quadrantanopia, is found. If patient is blind from any cause, score 3. Double simultaneous stimulation is performed in this case. If there is extinction, the patient receives a 1, and the results are used to respond to item 11. | 0=No visual loss.  1= Partial hemianopia.  2= Complete hemianopia.  3= Bilateral hemianopia (blind including cortical blindness) |
| 4. Facial palsy: Ask or use pantomime to encourage the patient to show teeth or raise eyebrows and close eyes. Score symmetry of grimace in response to noxious stimuli in the poorly response or non-comprehending patient. If facial trauma/bandages, orotracheal tube, tape or other physical barriers obscure the face, these should be removed to the extent possible. | 0 = Normal symmetrical movements.  1= Minor paralysis (flattened nasolabial fold, asymmetry on smiling)  2= Partial paralysis (total or near-total paralysis of lower face)  3= Complete paralysis of one or both sides (absence of facial movement in the upper and lower face). |
| 5. Motor arm: The limb is placed in the appropriate position: extend the arms (palms down) 90 degrees (if sitting) or 45 degrees (if supine). Drift is scored if the arm falls before 10 seconds. The aphasic patient is encouraged using urgency in the voice and pantomime, but not noxious stimulation. Each limb is tested in turn, beginning with the non-paretic arm. Only in the case of amputation or joint fusion at the shoulder, the examiner should record the score as untestable (UN), and clearly write the explanation for this choice. | 0= No drift; limb holds 90 (or 45) degrees for full 10 seconds.  1= Drift; limb holds 90 (or 45) degrees, but drifts down before full 10 seconds; does not hit bed or other support.  2= Some effort against gravity; limb cannot get to or maintain (if cued) 90 (or 45) degrees, drifts down to bed, but has some effort against gravity.  3= No effort against gravity; limb falls.  4= No movement.  UN = Amputation or joint fusion: explain:  5a = Left Arm.  5b = Right arm. |
| 6. Motor leg: The limb is placed in the appropriate position: hold the leg at 30 degrees (always tested supine). Drift is scored if the leg falls before 5 seconds. The aphasic patient is encouraged using urgency in the voice and pantomime, but not noxious stimulation. Each limb is tested in turn, beginning with the non-paretic leg. Only in the case of amputation or joint fusion at the hip, the examiner should record the score as untestable (UN), and clearly write the explanation for this  choice. | 0= No drift; leg holds 30-degree position for full 5seconds.  1= Drift; leg falls by the end of the 5-second period but does not hit bed.  2= Some effort against gravity; leg falls to bed by 5 seconds, but has some effort against gravity.  3= No effort against gravity; leg falls to bed immediately.  4= No movement.  UN = Amputation or joint fusion: explain:  6a. Left Leg  6b. Right Leg. |
| 7. Limb ataxia: This item is aimed at finding evidence of a unilateral cerebellar lesion. Test with eyes open. In case of visual defect, ensure testing is done in intact visual field. The finger-nose-finger and heel-shin tests are performed on both sides, and ataxia is scored only if present out of proportion to weakness. Ataxia is absent in the patient who cannot understand or is paralyzed. Only in the case of amputation or joint fusion, the examiner should record the score as untestable (UN), and clearly write the explanation for this choice. In case of blindness, test by having the patient touch nose from extended arm position. | 0=Absent.  1= Present in one limb.  2= Present in two limbs.  UN = Amputation or joint fusion: explain: |
| 8. Sensory: Sensation or grimace to pinprick when tested, or withdrawal from noxious stimulus in the obtunded or aphasic patient. Only sensory loss attributed to stroke is scored as abnormal and the examiner should test as many body areas (arms [not hands], legs, trunk, face) as needed to accurately check for hemisensory loss. A score of 2, 'severe or total sensory loss', should only be given when a severe or total loss of sensation can be clearly demonstrated. Stuporous and aphasic patients will, therefore, probably score 1 or 0. The patient with brainstem stroke who has bilateral loss of sensation is scored 2. If the patient does not respond and is quadriplegic, score 2. Patients in a coma (item 1a=3) are automatically given a 2 on this item. | 0= Normal; no sensory loss.  1= Mild-to-moderate sensory loss; patients feels pinprick is less sharp or is dull on the affected side; or there is a loss of superficial pain with pinprick, but patient is aware of being touched.  2= Severe to total sensory loss; patient is not aware of being touched in the face, arm and leg. |
| 9. Best language: A great deal of information about comprehension will be obtained during the preceding sections of the examination. For this scale item, the patient is asked to describe what is happening in the attached picture, to name the items on the attached naming sheet and to read from the attached list of sentences. Comprehension is judged from responses here, as well as to all of the commands in the preceding general neurological exam. If visual loss interferes with the tests, ask the patient to identify objects placed in the hand, repeat, and produce speech. The intubated patient should be asked to write. The patient in a coma (item 1a=3) will automatically score 3 on this item. The examiner must choose a score for the patient with stupor or limited cooperation, but a score of 3 should be used only if the patient is mute and follows no one-step commands. | 0= No aphasia; normal  1= Mild-to-moderate aphasia; some obvious loss of fluency or facility of comprehension, without significant limitation on ideas expressed or form of expression. Reduction of speech and/or comprehension, however, makes conservation about provided materials difficult or impossible. For example, in conversation about provided materials, examiner can identify picture or naming card content from patient's response.  2= Severe aphasia; all communication is through fragmentary expression; great need for inference, questioning, and guessing by the listener. Range of information that can be exchanged is limited; listener carries burden of communication. Examiner cannot identify materials provided from patient response.  3= Mute, global aphasia: no usable speech or auditory comprehension. |
| 10. Dysarthria: If patient is thought to be normal, an adequate sample of speech must be obtained by asking patient to read or repeat words from the attached list. If the patient has severe aphasia, the clarity of articulation of spontaneous speech can be rated. Only if patient is intubated or has other physical barriers to producing speech, the examiner should record the score as untestable (UN), and clearly write an explanation for this choice. Do not tell the patient why he or she is being tested. | 0= Normal.  1= Mild-to-moderate dysarthria; patient slurs at least some words and, at worst, can be understood by some difficulty.  2= Severe dysarthria: patient's speech is so slurred as to be unintelligible in the absence of or out of proportion to any dysphasia, or is mute/anarthric.  UN = Intubated or other physical barrier. |
| 11. Extinction and Inattention (formerly Neglect): Sufficient information to identify neglect may be obtained during the prior testing. If the patient has a severe visual loss preventing visual double simultaneous stimulation, and the cutaneous stimuli are normal, the score is normal. If the patient has aphasia but does appear to attend to both sides, the score is normal. The presence of visual spatial neglect or anosagnosia may also be taken as evidence of abnormality. Since the abnormality is scored only if present, the item is never untestable. | 0= No abnormality.  1= Visual, tactile, auditory, spatial, or personal inattention or extinction to bilateral simultaneous stimulation in one of the sensory modalities.  2= Profound hemi-inattention or extinction to more than one modality; does not recognize own hand or orients to only one side of space. |

**4.3 Modified Rankin Scale (mRS)**

**0** No symptoms at all

**1** No significant disability despite symptoms: able to carry out all usual duties and activities

**2** Slight disability: unable to carry out all previous activities but able to look after own affairs without assistance

**3** Moderate disability: requiring some help, but able to walk without assistance

**4** Moderately severe disability: unable to walk without assistance, and unable to attend to own bodily needs without assistance

**5** Severe disability: bedridden, incontinent, and requiring constant nursing care and attention

**6** Death

**4.4 Modified Barthel Index (mBI)**

| **Items** | **Codes** | | | | |
| --- | --- | --- | --- | --- | --- |
| any part of midbrain | 1  Unable to perform task | 2  Attempts task but unsafe | 3  Moderate help required | 4  Minimal help required | 5  Fully independent |
| Personal hygiene | 0 | 1 | 3 | 4 | 5 |
| Bathing self | 0 | 1 | 3 | 4 | 5 |
| Feeding | 0 | 2 | 5 | 8 | 10 |
| Toilet | 0 | 2 | 5 | 8 | 10 |
| Dressing | 0 | 2 | 5 | 8 | 10 |
| Bowel control | 0 | 2 | 5 | 8 | 10 |
| Bladder control | 0 | 2 | 5 | 8 | 10 |
| Stair climbing | 0 | 2 | 5 | 8 | 10 |
| Ambulation | 0 | 3 | 8 | 12 | 15 |
| Wheelchair | 0 | 1 | 3 | 4 | 5 |
| Chair/bed transfers | 0 | 3 | 8 | 12 | 15 |
| Range | 0……………………………………………..100 | | | | |

“Wheelchair” will be scored only if “Ambulation” coded “1” and patient trained in wheelchair management.

**4.5 Posterior Circulation Alberta Stroke Program Early CT Score (PC-ASPECTS)**

| **Items** | **Scores** |
| --- | --- |
| any part of midbrain | 2 |
| any part of pons | 2 |
| left thalamus | 1 |
| right thalamus | 1 |
| left cerebellum | 1 |
| right cerebellum | 1 |
| left posterior cerebral artery territory | 1 |
| right posterior cerebral artery territory | 1 |
| **Total** |  |

From 10 points, 1 or 2 points each (as indicated) are subtracted for early ischemic changes in above items. PC-ASPECTS 10 indicates a normal scan. PC-ASPECTS 0 indicates extensive early ischemic changes.

**4.6 Pons-midbrain-index(PMI)**

| **Items** | **Scores** |
| --- | --- |
| left midbrain | 2 |
| right midbrain | 2 |
| left pons | 2 |
| right pons | 2 |
| **Total** |  |

A PMI of zero indicates no hypo-density in either the midbrain or the pons. Each side of both the midbrain and pons was classified as: 0 = no hypodensity;1 = 50% or less hypodensity; 2 = more than 50% hypodensity. A PMI of eight indicated bilateral hypodensity of more than 50% in both the midbrain and the pons.

**4.7 Modified Treatment in Cerebral Ischemia (mTICI) scale**

| **Grades** | **Definitions** |
| --- | --- |
| 0 | no perfusion |
| 1 | antegrade reperfusion past the initial occlusion, but limited distal branch filling with little or slow distal reperfusion |
| 2a | antegrade reperfusion of less than half of the occluded target artery previously ischemic territory |
| 2b | antegrade reperfusion of more than half of the previously occluded target artery ischemic territory |
| 3 | complete antegrade reperfusion of the previously occluded target artery ischemic territory, with absence of visualized occlusion in all distal branches |

# **5. References:**

[1].	Jovin, T.G., et al., Trial of Thrombectomy 6 to 24 Hours after Stroke Due to Basilar-Artery Occlusion. N Engl J Med, 2022. 387(15): p. 1373-1384.

[2].	Tao, C., et al., Trial of Endovascular Treatment of Acute Basilar-Artery Occlusion. N Engl J Med, 2022. 387(15): p. 1361-1372.

[3].	Adusumilli, G., et al., Endovascular thrombectomy after acute ischemic stroke of the basilar artery: a meta-analysis of four randomized controlled trials. J Neurointerv Surg, 2023. 15(e3): p. e446-e451.

[4].	Pop, R., et al., Poor clinical outcome despite successful basilar occlusion recanalization in the early time window: incidence and predictors. J Neurointerv Surg, 2023. 15(5): p. 415-421.

[5].	Hussein, H.M., et al., Occurrence and predictors of futile recanalization following endovascular treatment among patients with acute ischemic stroke: a multicenter study. AJNR Am J Neuroradiol, 2010. 31(3): p. 454-8.

[6].	Drieu, A., et al., Immune Responses and Anti-inflammatory Strategies in a Clinically Relevant Model of Thromboembolic Ischemic Stroke with Reperfusion. Transl Stroke Res, 2020. 11(3): p. 481-495.

[7].	Naderi, Y., et al., Neuroprotective effects of minocycline on focal cerebral ischemia injury: a systematic review. Neural Regen Res, 2020. 15(5): p. 773-782.

[8].	Lampl, Y., et al., Minocycline treatment in acute stroke: an open-label, evaluator-blinded study. Neurology, 2007. 69(14): p. 1404-10.

[9].	Switzer, J.A., et al., Matrix metalloproteinase-9 in an exploratory trial of intravenous minocycline for acute ischemic stroke. Stroke, 2011. 42(9): p. 2633-5.

[10].	Kohler, E., et al., Intravenous minocycline in acute stroke: a randomized, controlled pilot study and meta-analysis. Stroke, 2013. 44(9): p. 2493-9.

[11].	Baik, S.H., et al., Futile recanalization in patients with basilar artery occlusion: assessment of the underlying etiology and the role of perfusion imaging. J Neurointerv Surg, 2024.

[12].	Fu, Y., et al., Fingolimod for the treatment of intracerebral hemorrhage: a 2-arm proof-of-concept study. JAMA Neurol, 2014. 71(9): p. 1092-101.

[13].	Tian, D.C., et al., Fingolimod enhances the efficacy of delayed alteplase administration in acute ischemic stroke by promoting anterograde reperfusion and retrograde collateral flow. Ann Neurol, 2018. 84(5): p. 717-728.

[14].	Nighoghossian, N., et al., Cyclosporine in acute ischemic stroke. Neurology, 2015. 84(22): p. 2216-23.

[15].	Elkins, J., et al., Safety and efficacy of natalizumab in patients with acute ischaemic stroke (ACTION): a randomised, placebo-controlled, double-blind phase 2 trial. Lancet Neurol, 2017. 16(3): p. 217-226.

[16].	Sandercock, P.A. and T. Soane, Corticosteroids for acute ischaemic stroke. Cochrane Database Syst Rev, 2011. 2011(9): p. CD000064.

[17].	Yang, Q., et al., Methylprednisolone as Adjunct to Endovascular Thrombectomy for Large-Vessel Occlusion Stroke: The MARVEL Randomized Clinical Trial. JAMA, 2024. 331(10): p. 840-849.

[18].	Strickland, B.A., et al., Neuroprotective effect of minocycline against acute brain injury in clinical practice: A systematic review. J Clin Neurosci, 2021. 86: p. 50-57.

[19].	Mulder, I.A., et al., Adjunctive cytoprotective therapies in acute ischemic stroke: a systematic review. Fluids Barriers CNS, 2021. 18(1): p. 46.

[20].	Zhang, M., et al., Ischemia-reperfusion injury: molecular mechanisms and therapeutic targets. Signal Transduct Target Ther, 2024. 9(1): p. 12.

**MIST-B Protocol summary of changes**

**Protocol Version 1.0 date:** 27th June 2022

**Protocol Version 2.0 date:** 22th November 2022

**Protocol Version 3.0 date:** 12th February 2025

| **Sections** | **Protocol Version 1.0** | **Protocol Version 2.0** | **Protocol Version 3.0** | **Rationale** |
| --- | --- | --- | --- | --- |
| 2.1 Design | Minocycline for acute Ischemic Stroke undergoing endovascular Treatment due to Basilar artery occlusion (MIST-B) is an evaluator-blinded, randomized, open-label, proof of concept trial.  The study has a planned duration of 15 months to recruit 40 patients in China. | ***Changed:***  Minocycline for acute Ischemic Stroke undergoing endovascular Treatment due to Basilar artery occlusion (MIST-B) is a multi-center, evaluator-blinded, randomized, open-label, proof of concept trial.  The study has a planned duration of 2.0 years to recruit 90 patients across 12 comprehensive stroke centers in China. | ***Changed:***  The study has a planned duration of 2.5 years to recruit 90 patients across 12 comprehensive stroke centers in China. | On November 22, 2022, before enrollment, the steering committee, in agreement with the DSMB, revised the study from single- to multicenter and increased the sample size to enhance external validity and clinical applicability. |
| 2.2 Patient population | Inclusion Criteria  7) Final mTICI score of 2b or 3 in BAO based on last digital subtraction angiography (DSA) run | ~~Final mTICI score of 2b or 3 in BAO based on last digital subtraction angiography (DSA) run~~ |  | On November 22, 2022, before enrollment, the steering committee, in agreement with the DSMB, removed this inclusion criterion to improve clinical applicability. |
| 2.3 Randomization | Block randomization will be used to randomize subjects. After occlusion of basilar artery has been confirmed and written informed consent has been obtained, patients will be immediately randomly assigned to adjunctive minocycline group or control group in a 1:1 ratio. | ***Added:***  Central randomization system will be used to randomize subjects. After occlusion of basilar artery has been confirmed and written informed consent has been obtained, patients will be immediately randomly assigned to adjunctive minocycline group or control group in a 1:1 ratio. Patients randomly assigned to Minocycline group were prescribed minocycline capsules as soon as possible after randomization, ideally before the EVT. The randomization sequence will be generated using SAS V.9.4 software. The randomization procedure is web based and runs on mobile devices or web page platforms(http://iwrs.xarsk.com/). The participating sites will be continuously and carefully monitored for crossovers and other protocol deviations. | ***Changed:***  Patients randomly assigned to Minocycline group were prescribed minocycline capsules as soon as possible after randomization, ideally before the EVT or no more than 1 hour after EVT. | To be consistent with the Change of Study Design and Plan. |
| 2.6 Primary outcomes | proportion of modified Rankin Scale (mRS) 0 to 3 at 90 days from randomization | ***Changed:***  The primary effectiveness outcome is expanded National Institutes of Health Stroke Scale (e-NIHSS) scores at 5 days. Given the variability in clinical practice across centers, e-NIHSS assessment scheduled for day 5 was permitted up to day 7 after randomization. For patients discharged before day 5, the e-NIHSS score was recorded at discharge. |  | On November 22, 2022, before enrollment, in agreement with the DSMB, the trial steering committee changed the primary outcome from 90-day mRS 0-3 to 5-day e-NIHSS. This change reflected the exploratory nature of the trial and the potential utility of e-NIHSS as a surrogate marker of early neurological deficits in acute basilar-artery stroke. |
| 2.7 Secondary outcomes | proportion of modified Thrombolysis In Cerebral Infarction(mTICI) 2b-3 after endovascular treatment. | ~~proportion of modified Thrombolysis In Cerebral Infarction(mTICI) 2b-3 after endovascular treatment.~~  ***Added:***  proportion of modified Rankin Scale (mRS) 0 to 3 at 90 days from randomization |  | To be consistent with the Change of Inclusion Criteria and Primary Efficacy Endpoints |
| 2.10 Sample size estimates | Considering the nature of exploring study in our trail, we plan to enroll 40 patients (20 patients in each group). | ***Changed:***  We use the NIHSS parameters in previous studies to estimate the sample size of this study. Therefore, we calculated that a sample size of 90 patients (45 patients in each group with a dropout rate of 10%) would detect a 3 points difference (16±5.3 for the control group and 13±5.3 for the intervention group) in e-NIHSS scores of patients at 5 days, with a power of 80% and two-sided significance levels of 0.05 for the analysis of the primary effectiveness outcome. Sample size was computed using PASS 11.0.7. |  | To improve the rigor of the trial: We based our estimations on the results of the ATTENTION trial. The means (SD) of NIHSS scores in patients with thrombectomy at 5 days is calculated as 16±5.3 by the methods of Iztok Hozo. Due to the lack of previous data on adjunctive minocycline in addition to EVT, it is considered a reasonable prediction that NIHSS scores in adjunctive minocycline group would be 3 points lower than those in control group at 5 days, based on the discussion by the Academic Committee and the Executive Committee. |
| 3.1 Trial site and Investigators | Chief Investigator:  Dr. Wen Jiang, Department of Neurology, Xijing Hospital, Fourth Military Medical University; | ***Added:***  14.1 Co-investigators:  Dr. Rong Yin, Department of Neurology, Gansu Provincial Maternity and Child-care Hospital (Gansu Provincial Central Hospital);  Dr. Jianbo He, Department of Neurology, Xi'an XD Group Hospital;  Dr. Liping Yu, Department of Neurology, First People's Hospital of Xianyang;  Dr. Yi Jia, Department of Neurology, Xi'an Gaoxin Hospital;  Dr. Mingze Chang, Department of Neurology, Xi'an Third Hospital; | ***Added or Changed:***  14.1 Co-investigators:  Dr. Rong Yin, Department of Neurology, Gansu Provincial Maternity and Child-care Hospital (Gansu Provincial Central Hospital);  Dr. Huiping Zhang, Department of Neurology, Baoji High-tech Hospital;  Dr. Li Yao, Department of Neurology, Xi'an XD Group Hospital;  Dr. Weiwang Li, Department of Neurology, Xi'an Daxing Hospital;  Dr. Liping Yu, Department of Neurology, First People's Hospital of Xianyang;  Dr. Xiangjun Yuan, Department of Neurology, Weinan Central Hospital;  Dr. Yi Jia, Department of Neurology, Xi'an Gaoxin Hospital;  ~~Dr. Mingze Chang, Department of Neurology, Xi'an Third Hospital;~~  Dr. Bo Song, Department of Neurosurgery, Xianyang Hospital of Yan'an University;  Dr. Baiya Fan, Department of Neurology, Yan'an People's Hospital;  Dr. Xingshun Ma, Department of Neurology, The First Hospital of Yulin;  Dr. Xue Li, Department of Neurology, Yulin Hospital, The First Affiliated Hospital of Xi'an Jiaotong University. | To be consistent with the Change of overall study design, and to improve the rigor of the trial. |
| 3.2 Statisticians | Pro. Lei Shang and Pro. Ling Wang, Department of Health Statistics, Fourth Military Medical University; | ***Added:***  Pro. Duolao Wang, Department of Clinical Sciences, Liverpool School of Tropical Medicine;  Pro. Bohui Zhang, Department of Cardiology, Fourth Military Medical University. |  | To be consistent with the Change of overall study design, and to improve the rigor of the trial. |
| 3.3 Academic Committee |  | ***Added:***  Dr. Wen Jiang and Dr. Dong Wei, Department of Neurology, Xijing Hospital, Fourth Military Medical University;  Pro. Lei Shang and Pro. Ling Wang, Department of Health Statistics, Fourth Military Medical University;  Dr. Hongzeng Li, Department of Neurology, Tangdu Hospital, Fourth Military Medical University;  Dr. Jianbo He, Department of Neurology, Xi'an XD Group Hospital;  Dr. Mingze Chang, Department of Neurology, Xi'an Third Hospital;  Dr. Yi Jia, Department of Neurology, Xi'an Gaoxin Hospital;  Dr. Rong Yin, Gansu Provincial Maternity and Child-care Hospital (Gansu Provincial Central Hospital);  Dr. Liping Yu, Department of Neurology, First People's Hospital of Xianyang. |  | To be consistent with the Change of overall study design, and to improve the rigor of the trial. |
| 3.4 Executive Committee |  | ***Added:***  Dr. Wen Jiang, Dr. Dong Wei, Dr. Rui Shi, Dr. Deshuai Li, Dr. Zhirong Fan, Department of Neurology, Xijing Hospital, Fourth Military Medical University;  Dr. Jianbo He and Dr. Li Yao, Department of Neurology, Xi'an XD Group Hospital;  Dr. Mingze Chang, Department of Neurology, Xi'an Third Hospital;  Dr. Yi Jia, Department of Neurology, Xi'an Gaoxin Hospital;  Dr. Rong Yin, Department of Neurology, Gansu Provincial Maternity and Child-care Hospital (Gansu Provincial Central Hospital);  Dr. Liping Yu, Department of Neurology, First People's Hospital of Xianyang. | ***Added:***  Dr. Yang Li, Dr. Luojun Wang and Dr. Jingjing Zhao, Department of Neurology, Xijing Hospital, Fourth Military Medical University;  Pro. Lei Shang and Pro. Li Wang, Department of Health Statistics, Fourth Military Medical University; | To be consistent with the Change of overall study design, and to improve the rigor of the trial. |
| 3.5 Independent assessors in each centre | Dr. Le Wang, Department of Neurology, Xijing Hospital, Fourth Military Medical University; | ***Added:***  Dr. Yang Li, Department of Neurology, Xijing Hospital, Fourth Military Medical University;  Dr. Yaxuan Wei, Department of Neurology, Gansu Provincial Maternity and Child-care Hospital (Gansu Provincial Central Hospital);  Dr. Juanli Zhang, Department of Neurology, Xi'an XD Group Hospital;  Dr. Hao Zhu, Department of Neurology, Xiangjun Yuan, First People's Hospital of Xianyang;  Dr. Saibing Liu, Department of Neurology, Xi'an Gaoxin Hospital;  Dr. Shilin Zhang, Department of Neurology, Department of Neurology, Xi'an Third Hospital. | ***Added or Changed:***  Dr. Le Wang, Department of Neurology, Xijing Hospital, Fourth Military Medical University;  ~~Dr. Yang Li, Department of Neurology, Xijing Hospital, Air Force Medical University;~~  Dr. Yaxuan Wei, Department of Neurology, Gansu Provincial Maternity and Child-care Hospital (Gansu Provincial Central Hospital);  Dr. Yanan Yang, Department of Neurology, Baoji High-tech Hospital;  Dr. Juanli Zhang, Department of Neurology, Xi'an XD Group Hospital;  Dr. Haixia Pei, Department of Neurology, Xi'an Daxing Hospital;  Dr. Hao Zhu, Department of Neurology, First People's Hospital of Xianyang;  Dr. Xingyun Yuan, Department of Neurology, First People's Hospital of Xianyang;  Dr. Huijuan Shang, Department of Neurology, Weinan Central Hospital;  Dr. Bao Guo, Department of Neurology, Weinan Central Hospital;  Dr. Saibing Liu, Department of Neurology, Xi'an Gaoxin Hospital;  ~~Dr. Shilin Zhang, Department of Neurology, Xi'an Third Hospital.~~  Dr. Jian Miao, Department of Neurosurgery, Xianyang Hospital of Yan'an University;  Dr. Hairong Gao, Department of Neurology, Yan 'an People's Hospital;  Dr. Hong Zhang, Department of Neurology, The First Hospital of Yulin;  Dr. Hufei Chang, Department of Neurology, Yulin Hospital, The First Affiliated Hospital of Xi'an Jiaotong University. | To be consistent with the Change of overall study design, and to improve the rigor of the trial. |
