# Supplementary material for: Minocycline for acute basilar artery occlusion stroke undergoing endovascular treatment: a randomized, evaluator-blinded, open-label, pilot trial
Source: BMC Med. 2026 Jul 27;24:580. doi: 10.1186/s12916-026-05092-4 (PMC13688725; doi:10.1186/s12916-026-05092-4)
Supplement: Supplementary file 2 [file 12916_2026_5092_MOESM2_ESM.docx]

**This supplement contains the following items**

**1. Statistical analysis plan**

**a. Original statistical analysis plan, version 1.0 (p 2 - 16)**

**b. Final statistical analysis plan, version 3.0 (p 17 - 38)**

**c. Summary of changes between versions 1 and 3 (p 39 - 50)**

**STATISTICAL ANALYSIS PLAN**

**Minocycline for acute basilar artery occlusion stroke undergoing endovascular treatment: a randomized, evaluator-blinded, open-label, pilot trial**

**27th June 2022**

**Version 1.0**

**TABLE OF CONTENTS**

[**1. Abbreviations and Definitions of Terms	1**](#_Toc206974931)

[**2. Preface	2**](#_Toc206974932)

[**3. Purpose of SAP	2**](#_Toc206974933)

[**4. Validation of SAP and amendments	3**](#_Toc206974934)

[**5. Study endpoints	3**](#_Toc206974935)

[**6. Study Methods	5**](#_Toc206974936)

[**7. Formal Analyses and Reporting	6**](#_Toc206974937)

[**8. Sample Size Determination	6**](#_Toc206974938)

[**9. Analysis Populations	7**](#_Toc206974939)

[**10. General Issues for Statistical Analysis	8**](#_Toc206974940)

[**11. Disposition of Subjects and Withdrawals	9**](#_Toc206974941)

[**12. Demographics and Baseline Characteristics	10**](#_Toc206974942)

[**13. Efficacy Analyses	11**](#_Toc206974943)

[**14. Safety Analyses	11**](#_Toc206974944)

[**15. References	13**](#_Toc206974945)

| **Abbreviations and Definitions of Terms** | |
| --- | --- |
| **SAP** | Statistical Analysis Plan |
| **EVT** | Endovascular treatment |
| **ICH-E9** | International Conference on Harmonization of Technical Requirements for Registration of Pharmaceuticals for Human Use |
| **CRF** | Case report form |
| **e-NIHSS** | expanded National Institutes of Health Stroke Scale |
| **NIHSS** | National Institutes of Health Stroke Scale |
| **mRS** | modified Rankin Scale |
| **sICH** | symptomatic Intracranial Hemorrhage |
| **mBI** | modified Barthel index |
| **ICU** | Intensive Care Unit |
| **pc-ASPECTS** | posterior circulation Alberta Stroke Program Early CT score |
| **PMI** | Pons-midbrain-index |
| **DSMB** | Data and Safety Management Board |
| **ITT** | Intention-to-Treat |
| **mITT** | modified intention-to-treat |
| **PP** | Per-Protocol |
| **CIs** | confidence intervals |
| **AE** | Adverse Event |
| **SAE** | Serious Adverse Event |

# **1.Preface**

This Statistical Analysis Plan (SAP) describes the planned analysis and reporting for protocol Clinicaltrials.gov (NCT05512910) entitled “Minocycline for acute Ischemic Stroke undergoing endovascular Treatment due to Basilar artery occlusion: a randomized, open-label, proof of concept study” (Version 1.0, 2022/06/27).

The primary objective of this study is being completed to assess the efficacy and safety of adjunctive oral minocycline in patients with acute ischemic stroke due to acute basilar artery occlusion in the setting of receiving endovascular treatment (EVT).

This SAP is structured and detailed to comply with the regulatory requirements set forth by the National Medical Products Administration and the International Conference on Harmonization of Technical Requirements for Registration of Pharmaceuticals for Human Use (ICH-E9) guidelines on statistical principles in clinical trials^[1]^. All planned and reported analyses within this SAP adhere to internationally recognized standards for statistical practice, as published by the American Statistical Association ^[2]^.

Readers of this SAP are encouraged to also read the clinical trial protocol to understand the implementation details of this study, the operational aspects of clinical evaluation, and the schedule of patients completing this study.

# **2.Purpose of SAP**

The objective of this SAP is to provide a comprehensive framework for the analyses that will be conducted to support the finalization of the clinical study report. The analyses detailed in this SAP are intended to be incorporated into regulatory submissions and/or future publications. Additionally, exploratory analyses that are not explicitly outlined in this SAP may also be performed to further inform the clinical development plan. Any analyses that are conducted post hoc or are not planned within the scope of this SAP will be distinctly labeled and described in the relevant clinical study report.

# **3. Validation of SAP and amendments**

The SAP should be reviewed by Principal investigator(s), Chair of Steering Committee, Chair of Data Management Committee and Statistician.

After approval, the SAP may be amended, and amendments are only permitted prior to the last follow-up finished and unblinding of the treatment code. All changes to the SAP will be meticulously tracked and documented.

# **4. Study endpoints**

**4.1 Primary Efficacy and Safety Endpoints**

**4.1.1 Primary Efficacy Endpoint**

The primary efficacy outcome measure for this study is: proportion of modified Rankin Scale (mRS) 0 to 3 at 90 days from randomization (Binary data).

**4.1.2 Primary Safety Endpoints**

The primary safety outcome measure for this study is: the proportion of patients with symptomatic intracranial hemorrhage (sICH) at 24 hours from randomization (Binary data).

**4.2 Secondary Efficacy and Safety Endpoints**

**4.2.1 Secondary Efficacy Endpoints**

1) the measure of the global disability defined as a mRS score at 90 days from randomization (Ordinal data).

2) proportion of mRS score 0 to 2 at 90 days from randomization (Binary data).

3) proportion of mRS score 0 to 1 at 90 days from randomization (Binary data).

4) National Institutes of Health Stroke Scale (NIHSS) at 24 hours from randomization (Continuous data).

5) NIHSS at 5 days from randomization (Continuous data).

6) NIHSS at 30 days from randomization (Continuous data).

7) NIHSS at 90 days from randomization (Continuous data).

8) expanded National Institutes of Health Stroke Scale (e-NIHSS) at 24 hours from randomization (Continuous data).

9) e-NIHSS at 5 days from randomization (Continuous data).

10) e-NIHSS at 30 days from randomization (Continuous data).

11) e-NIHSS at 90 days from randomization (Continuous data).

12) modified Barthel index (mBI) scores at 30 days from randomization (Continuous data).

13) mBI scores at 90 days from randomization (Continuous data).

14) proportion of modified Thrombolysis In Cerebral Infarction(mTICI) 2b-3 after endovascular treatment (Binary data).

15) changes in infarct volume from baseline to 5 days (Continuous data).

16) length of Intensive Care Unit (ICU) stay and hospital stay (Continuous data).

17) time of mechanical ventilation or non-invasive ventilation at 5 days (Continuous data).

18) proportion of pneumonia at 5 days (Binary data).

19) changes in percentage of the lymphocyte subpopulations, matrix metalloproteinase-9 (MMP-9) value, interleukin-6 (IL-6) value, interleukin (IL-10) value, tumor necrosis factor-α (TNF-α) value, leucocyte counts, percentage of neutrophilic granulocyte and absolute neutrophil value at 5 days (Continuous data).

**4.2.2 Secondary Safety Endpoints**

1) proportion of sICH at 3 days from randomization (Binary data).

2) proportion of mortality due to any cause at 90 days from randomization (Binary data).

3) proportion of patients with any adverse events (Binary data).

4) proportion of patients with any serious adverse events (Binary data).

# **5. Study Methods**

**5.1 Overall Study Design and Plan**

Minocycline for acute Ischemic Stroke undergoing endovascular Treatment due to Basilar artery occlusion (MIST-B) is a prospective, randomized, open-label clinical trial with blinded outcome assessment. All enrolled patients will be randomized with 1:1 to EVT only or oral minocycline adjunctively to EVT within 24 hours of symptom onset and receive continuous application for 5 days. The study has a planned duration of 15 months to recruit 40 patients in China. The follow-up period covers the period from the acute stage to the final follow-up after randomization (24 hours, 5 days, 30 days, 90 days) which defines the primary endpoints. Eligibility for study participation will be determined by evaluating the inclusion and exclusion criteria using the data collected during the screening period.

# **6. Formal Analyses and Reporting**

All formal planned analyses identified in the protocol and in this SAP will be performed only after records locked and the study finished. Before records locked and completion of the final analyses, a blinded data will be checked and reviewed. Also, the SAP should be approved and signed before data locked, random code unblinded and analyses completed.

All post-hoc and exploratory analyses conducted to supplement the planned study analyses, but not specified in this SAP, will be meticulously documented and presented in the appendices. Additionally, the results of these unplanned analyses will be distinctly labeled and highlighted within the appendices.

# **7. Sample Size Determination**

The primary hypothesis is that patients receiving adjunctive minocycline in addition to EVT within 24 hours after stroke onset, will have a higher proportion of functional independence (mRS 0-3) at 90 days. Considering the nature of exploring study in our trail, we plan to enroll 40 patients (20 patients in each group).

# **8. Analysis Populations**

Intention-to-Treat (ITT) population, Per-Protocol (PP) population and Safety population are planned for the studies.

The primary analyses of the efficacy outcomes will be based on the ITT population. The PP population will be used in the efficacy and sensitivity analysis. The Safety population will be used in the safety analysis.

1) ITT population includes all randomized patients regardless of the care they received or any amount of study drug, even if the subject does not receive the assigned treatment. Participants who withdrew consent or were judged ineligible by the Data and Safety Management Board (DSMB) were excluded. All remaining randomized patients comprised the modified intention-to-treat (mITT) population.

2) PP population includes patients who received the assigned treatment and do not have major protocol violations or deviations. The patient in the intervention group is determined to have received the intended intervention if the first 200mg of oral minocycline were feed before the recanalization during EVT, followed by 100 mg every 12 hours times for a total of 5 days. Major deviation from the treatment regimen is defined as the subject not receiving any assigned treatment, unless the subject met the criteria for treatment discontinuation. If a patient is unable to complete the 5-days course of oral minocycline treatment due to adverse events or death within the first 5 days after randomization, the patient will still be considered as part of the assigned treatment group for analysis purposes.

3) Safety population includes all patients who were randomized and received EVT treatment, and for whom at least one follow-up safety assessment was recorded. Patients will be classified according to the actually treatment they received.

Note: Patients who were earlier randomized and treated, but lost to follow up, will be included to the time of dropout.

# **9. General Issues for Statistical Analysis**

**9.1 Analysis Software**

All statistical analyses will be performed using Python version 3.12 and R version 4.3.1.

**9.2 Methods for Withdrawals and Missing Data**

**9.2.1 Withdrawals**

If any subject withdraws from the study prematurely (before the visit 5 day for evaluation), they will be excluded from any analysis and are required to complete the withdrawal visit in the Case Report Form (CRF). The reasons for withdrawal will be listed in a summary table.

**9.2.2 Missing Data in primary analysis**

Clinical assessment and imaging examination will be conducted on time to minimize the amount of missing primary outcomes (less than 5%). During the follow-up stage outside the hospital, the patients or their families and doctors will keep phone numbers for each other, to remind regularly face-to-face visits and avoid loss to follow-up.

Patients with missing data of primary outcomes will be censored in primary analyses. The analyses of secondary efficacy and safety outcomes will be conducted in the complete case. If the proportion of subjects with missing values for outcomes is higher than 20%, the analysis of such variable will be omitted from the study.

For patients who died within the study period, the worst scores will be assigned for all not-assessed clinical outcome measures in their analyses, as follows:

1) e-NIHSS 42

2) NIHSS 42

3) mRS 6

4) mBI 0

**9.3 Presentation of Results**

The continuous variables will be described as mean, standard deviation, median, first and third quartiles, and range. For categorical variables, the description will consist of the frequency and percentage. For ordered variables, the description will cover the frequency, percentage, and cumulative percentage.

# **10. Disposition of Subjects and Withdrawals**

The number of patients for each of the following categories will be summarized.

 Total number of assessed patients

 Number of randomized patients

Number of patients completing the study and not completing the study (grouped by treatment and main reason)

 Number of patients included in the ITT population

 Number of patients included in the PP population

Number of patients included in the Safety population

# **11. Demographics and Baseline Characteristics**

Descriptive statistics will be used to summarize clinically important demographic and disease characteristics at screening for each treatment group. These summaries will be generated for both the ITT population and the PP population. If the proportion of missing values exceeds 5%, the denominator used in the calculations will be specified in a footnote of the corresponding summary table.

The variables to be summarized include but are not restricted to the following: Age; Sex; Ethnicity; Body-mass index; Medical History (hypertension, diabetes mellitus, atrial fibrillation, cerebral stroke, alcohol use and smoking); Pre stroke onset mRS score; Baseline NIHSS score; Baseline e-NIHSS score; Baseline posterior circulation Alberta Stroke Program Early CT score (pc-ASPECTS) Score; Baseline Pons-midbrain-index (PMI); Systolic and Diastolic Blood Pressure at hospital arrival; Glucose level at hospital arrival; TOAST etiology; Occlusion Sites; Occlusion site based on DSA; First-line thrombectomy strategy; mTICI score based on DSA run; Intravenous Thrombolysis; Administration of minocycline; Time from stroke onset (or last known well) to randomization, puncture, and recanalization; Time from hospital admission to puncture; Time from groin puncture to recanalization.

# **12. Efficacy Analyses**

The analyses of outcomes will be based on the ITT population. Patients with missing data will be censored in the primary analysis. Continuous outcomes will be analyzed using linear regression or generalized linear models as appropriate. Categorical outcomes will be analyzed using modified Poisson regression models. Ordinal outcomes will be analyzed using multivariable ordinal logistic regression. The effect parameter will be reported as the risk ratio, the common odds ratio or mean differences as appropriate, along with its 95% confidence intervals (CIs).

# **13. Safety Analyses**

Safety analyses will be performed exclusively on the Safety population. Categorical outcomes will be analyzed using modified Poisson regression models. The effect parameter of the secondary outcomes will be reported as the RR, along with its 95% CIs.

**13.1 Adverse Events**

Any unfavorable change in the health status of a trial participant, or the emergence or worsening of any undesirable sign, symptom, or medical condition following enrollment, will be classified as an Adverse Event (AE). All AEs must be documented, regardless of their perceived relationship to the study interventions. Pre-existing medical conditions are excluded from AE reporting.

Considering the temporal association, consistency, intensity, and specificity between AEs and the administration of EVT and/or minocycline treatment, the causality assessment of AEs will be classified into five categories: definitely related; probably related; possibly related; probably unrelated; definitely unrelated. The incidence rate of adverse reactions will be calculated based on the sum of events categorized as definitely related, probably related and possibly related.

The safety of the intervention will be monitored by recording acute adverse events at 24 hours, 5 days, 30 days and 90 days from randomization as a trial outcome. These events will be monitored periodically by the DSMB and will be captured separately as AEs in the CRF.

**13.2 Serious Adverse Events**

A Serious Adverse Event (SAE) is defined as an untoward occurrence that meets any of the following criteria: results in death, is life-threatening, requires or prolongs hospitalization, or results in persistent or significant disability or incapacity.

Any SAE experienced by a study participant must be reported to the sponsor if the Chief Investigator determines that the event is related to the study procedures or is unexpected. A related SAE is one that arises from the administration of any study intervention. An unexpected SAE is an event that is not listed in the protocol as a foreseeable occurrence.

All deaths occurring during the follow-up period up to Day 90 will be reported as SAEs. When reporting a death, the underlying event or condition that caused or contributed to the fatal outcome should be documented as a single medical concept.

# **14. References**

[1].	STATISTICAL PRINCIPLES FOR CLINICAL TRIALS.

[2].	Ethical Guidelines for Statistical Practice.

**STATISTICAL ANALYSIS PLAN**

**Minocycline for acute basilar artery occlusion stroke undergoing endovascular treatment: a randomized, evaluator-blinded, open-label, pilot trial**

**12th February 2025**

**Version 3.0**

**TABLE OF CONTENTS**

**[1. Abbreviations and Definitions of Terms	1](#_Toc206975382)**

[**2. Preface	2**](#_Toc206975383)

[**3. Purpose of SAP	3**](#_Toc206975384)

[**4. Validation of SAP and amendments	3**](#_Toc206975385)

[**5. Study endpoints	3**](#_Toc206975386)

[**6. Study Methods	6**](#_Toc206975387)

[**7. Formal Analyses and Reporting	6**](#_Toc206975388)

[**8. Sample Size Determination	7**](#_Toc206975389)

[**9. Analysis Populations	8**](#_Toc206975390)

[**10. General Issues for Statistical Analysis	9**](#_Toc206975391)

[**11. Disposition of Subjects and Withdrawals	13**](#_Toc206975392)

[**12. Demographics and Baseline Characteristics	13**](#_Toc206975393)

[**13. Efficacy Analyses	14**](#_Toc206975394)

[**14. Safety Analyses	17**](#_Toc206975395)

[**15. References	19**](#_Toc206975396)

| **Abbreviations and Definitions of Terms** | |
| --- | --- |
| **SAP** | Statistical Analysis Plan |
| **EVT** | Endovascular treatment |
| **ICH-E9** | International Conference on Harmonization of Technical Requirements for Registration of Pharmaceuticals for Human Use |
| **CRF** | Case report form |
| **e-NIHSS** | expanded National Institutes of Health Stroke Scale |
| **NIHSS** | National Institutes of Health Stroke Scale |
| **mRS** | modified Rankin Scale |
| **sICH** | symptomatic Intracranial Hemorrhage |
| **mBI** | modified Barthel index |
| **pc-ASPECTS** | posterior circulation Alberta Stroke Program Early CT score |
| **PMI** | Pons-midbrain-index |
| **DSMB** | Data and Safety Management Board |
| **ITT** | Intention-to-Treat |
| **mITT** | modified intention-to-treat |
| **PP** | Per-Protocol |
| **CIs** | confidence intervals |
| **AE** | Adverse Event |
| **SAE** | Serious Adverse Event |

# **1.Preface**

This Statistical Analysis Plan (SAP) describes the planned analysis and reporting for protocol Clinicaltrials.gov (NCT05512910) entitled “Minocycline for acute Ischemic Stroke undergoing endovascular Treatment due to Basilar artery occlusion: a randomized, open-label, proof of concept study” (Version 3.0, 2025/01/12).

The primary objective of this study is being completed to assess the efficacy and safety of adjunctive oral minocycline in patients with acute ischemic stroke due to acute basilar artery occlusion in the setting of receiving endovascular treatment (EVT).

This SAP is structured and detailed to comply with the regulatory requirements set forth by the National Medical Products Administration and the International Conference on Harmonization of Technical Requirements for Registration of Pharmaceuticals for Human Use (ICH-E9) guidelines on statistical principles in clinical trials^[1]^ . All planned and reported analyses within this SAP adhere to internationally recognized standards for statistical practice, as published by the American Statistical Association ^[2]^ .

Readers of this SAP are encouraged to also read the clinical trial protocol to understand the implementation details of this study, the operational aspects of clinical evaluation, and the schedule of patients completing this study.

# **2.Purpose of SAP**

The objective of this SAP is to provide a comprehensive framework for the analyses that will be conducted to support the finalization of the clinical study report. The analyses detailed in this SAP are intended to be incorporated into regulatory submissions and/or future publications. Additionally, exploratory analyses that are not explicitly outlined in this SAP may also be performed to further inform the clinical development plan. Any analyses that are conducted post hoc or are not planned within the scope of this SAP will be distinctly labeled and described in the relevant clinical study report.

# **3. Validation of SAP and amendments**

The SAP should be reviewed by Principal investigator(s), Chair of Steering Committee, Chair of Data Management Committee and Statistician.

After approval, the SAP may be amended, and amendments are only permitted prior to the last follow-up finished and unblinding of the treatment code. All changes to the SAP will be meticulously tracked and documented.

# **4. Study endpoints**

**4.1 Primary Efficacy and Safety Endpoints**

**4.1.1 Primary Efficacy Endpoint**

The primary efficacy outcome measure for this study is: expanded National Institutes of Health Stroke Scale (e-NIHSS) scores at 5 days (Continuous data). As an outcome in acute ischemic stroke RCTs, National Institutes of Health Stroke Scale (NIHSS) scores within the first 5-7 days have an intermediate-to-good agreement with modified Rankin Scale (mRS) score at 90 days^[3]^ . The e-NIHSS score is a modified NIHSS score focused on posterior circulation strokes, with the advantage to improve sensitivity of outcome assessment^[4]^ . Adopting the e-NIHSS score at 5 days as the primary efficacy endpoint in this trial offers several key advantages. Firstly, it significantly shortens the required follow-up duration, thereby reducing the operational costs associated with investigator-initiated trials. Additionally, the use of the early e-NIHSS assessment also minimizes interference from confounding factors, and thus allows for a better estimation of the direct treatment effect of the adjunctive oral minocycline on outcome. Moreover, using early e-NIHSS score as a primary outcome may allow for a smaller sample size. Lastly, utilizing e-NIHSS instead of NIHSS can enhance the sensitivity of outcome assessment, particularly in cases of posterior circulation strokes.

**4.1.2 Primary Safety Endpoints**

The primary safety outcome measure for this study is: the proportion of patients with symptomatic intracranial hemorrhage (sICH) at 24 hours from randomization (Binary data). sICH is defined as local or remote parenchymal hemorrhage type 2, subarachnoid hemorrhage, and/or intraventricular hemorrhage on the post-treatment imaging scan, combined with a neurological deterioration of 4 points or more on the NIHSS from baseline, or leading to death.

**4.2 Secondary Efficacy and Safety Endpoints**

**4.2.1 Secondary Efficacy Endpoints**

1) the measure of the global disability defined as a mRS score at 90 days from randomization (Ordinal data).

2) proportion of mRS score 0 to 3 at 90 days from randomization (Binary data).

3) proportion of mRS score 0 to 2 at 90 days from randomization (Binary data).

4) proportion of mRS score 0 to 1 at 90 days from randomization (Binary data).

5) NIHSS at 24 hours from randomization (Continuous data).

6) NIHSS at 5 days (Continuous data).

7) NIHSS at 30 days from randomization (Continuous data).

8) NIHSS at 90 days from randomization (Continuous data).

9) e-NIHSS at 24 hours from randomization (Continuous data).

10) e-NIHSS at 30 days from randomization (Continuous data).

11) e-NIHSS at 90 days from randomization (Continuous data).

12) modified Barthel index (mBI) scores at 30 days from randomization (Continuous data).

13) mBI scores at 90 days from randomization (Continuous data).

14) changes in infarct volume from baseline to 5 days (Continuous data).

15) length of Intensive Care Unit stay and hospital stay (Continuous data).

16) rate of mechanical ventilation at 5 days (Binary data).

17) proportion of pneumonia at 5 days (Binary data).

18) percentage of the lymphocyte subpopulations, matrix metalloproteinase-9 (MMP-9) value, interleukin-6 (IL-6) value, interleukin-10 (IL-10) value, tumor necrosis factor-α (TNF-α) value, leucocyte counts, percentage of neutrophilic granulocyte and absolute neutrophil value at 5 days (Continuous data).

**4.2.2 Secondary Safety Endpoints**

1) proportion of sICH at 3 days from randomization (Binary data).

2) proportion of mortality due to any cause at 90 days from randomization (Binary data).

3) proportion of patients with any adverse events (Binary data).

4) proportion of patients with any serious adverse events (Binary data).

# **5. Overall Study Design and Plan**

Minocycline for acute Ischemic Stroke undergoing endovascular Treatment due to Basilar artery occlusion (MIST-B) is a multicenter, prospective, randomized, open-label clinical trial with blinded outcome assessment. All enrolled patients will be randomized with 1:1 to EVT only or oral minocycline adjunctively to EVT within 24 hours of symptom onset and receive continuous application for 5 days. The study has a planned duration of 2.5 years to recruit 90 patients across 12 comprehensive stroke centers in China. The follow-up period covers the period from the acute stage to the final follow-up after randomization (24 hours, 5 days, 30 days, 90 days) which defines the primary endpoints.

# **6. Formal Analyses and Reporting**

All formal planned analyses identified in the protocol and in this SAP will be performed only after records locked and the study finished. Before records locked and completion of the final analyses, a blinded data will be checked and reviewed. Also, the SAP should be approved and signed before data locked, random code unblinded and analyses completed.

All post-hoc and exploratory analyses conducted to supplement the planned study analyses, but not specified in this SAP, will be meticulously documented and presented in the appendices. Additionally, the results of these unplanned analyses will be distinctly labeled and highlighted within the appendices.

# **7. Sample Size Determination**

The primary hypothesis is that patients receiving adjunctive minocycline in addition to EVT within 24 hours after stroke onset, will have better neurological outcome at 5 days. We based our estimations on the results of the ATTENTION trial^[5]^ . The means (SD) of NIHSS scores in patients with thrombectomy at 5 days is calculated as 16±5.3 by the methods of Iztok Hozo^[6]^ . Due to the lack of previous data on adjunctive minocycline in addition to EVT, it is considered a reasonable prediction that NIHSS scores in adjunctive minocycline group would be 3 points lower than those in control group at 5 days, based on the discussion by the Academic Committee and the Executive Committee. The e-NHISS scores, the main endpoint of efficacy set in this study, could improve the sensitivity of NIHSS scores in posterior circulation stroke and could have an impact on clinical trials. We use the NIHSS parameters in previous studies to estimate the sample size of this study. Therefore, we calculated that a sample size of 90 patients (45 patients in each group with a dropout rate of 10%) would detect a 3 points difference (16±5.3 for the control group and 13±5.3 for the intervention group) in e-NIHSS scores of patients at 5 days, with a power of 80% and two-sided significance levels of 0.05 for the analysis of the primary effectiveness outcome. Sample size was computed using PASS 11.0.7.

# **8. Analysis Populations**

Intention-to-Treat (ITT) population, Per-Protocol (PP) population and Safety population are planned for the studies^[7]^ .

The primary analyses of the efficacy outcomes will be based on the ITT population. The PP population will be used in the efficacy and sensitivity analysis. The Safety population will be used in the safety analysis.

1) ITT population includes all randomized patients regardless of the care they received or any amount of study drug, even if the subject does not receive the assigned treatment. Participants who withdrew consent or were judged ineligible by the Data and Safety Management Board (DSMB) were excluded. All remaining randomized patients comprised the modified intention-to-treat (mITT) population.

2) PP population includes patients who received the assigned treatment and do not have major protocol violations or deviations. The patient in the intervention group is determined to have received the intended intervention if the first 200mg of oral minocycline were feed before the EVT or within one hour after EVT, followed by 100 mg every 12 hours times for a total of 5 days. Major deviation from the treatment regimen is defined as the subject not receiving any assigned treatment, unless the subject met the criteria for treatment discontinuation. If a patient is unable to complete the 5-days course of oral minocycline treatment due to adverse events or death within the first 5 days after randomization, the patient will still be considered as part of the assigned treatment group for analysis purposes.

3) Safety population includes all patients who were randomized and received EVT treatment, and for whom at least one follow-up safety assessment was recorded. Patients will be classified according to the actually treatment they received.

Note: Patients who were earlier randomized and treated, but lost to follow up, will be included to the time of dropout.

# **9. General Issues for Statistical Analysis**

**9.1 Analysis Software**

All statistical analyses will be performed using Python version 3.12 and R version 4.3.1.

**9.2 Methods for Withdrawals and Missing Data**

**9.2.1 Withdrawals**

If any subject withdraws from the study prematurely (before the visit 5 day for evaluation), they will be excluded from any analysis and are required to complete the withdrawal visit in the Case Report Form (CRF). The reasons for withdrawal will be listed in a summary table.

**9.2.2 Missing Data**

**9.2.2.1 Outcomes in primary analysis**

The primary efficacy and safety outcomes in this trail are based on in-hospital data. Clinical assessment and imaging examination will be conducted on time to minimize the amount of missing primary outcomes (less than 5%). The day-5 endpoint was required to be assessed within 48 hours of the scheduled visit. For patients discharged prior to 5-day visit, clinical assessments at discharge were recorded. During the follow-up stage outside the hospital, the patients or their families and doctors will keep phone numbers for each other, to remind regularly face-to-face visits and avoid loss to follow-up. The day-30 outcome was allowed a time window of ±7 days, and the day-90 outcome a window of ±14 days from the target date.

Patients with missing data of primary outcomes will be censored in primary analyses. The analyses of secondary efficacy and safety outcomes will be conducted in the complete case. If the proportion of subjects with missing values for outcomes is higher than 20%, the analysis of such variable will be omitted from the study.

For patients who died within the study period, the worst scores will be assigned for all not-assessed clinical outcome measures in their analyses, as follows:

1) e-NIHSS 42

2) NIHSS 42

3) mRS 6

4) mBI 0

**9.2.2.2 Variables in covariate-adjusted analysis**

In the covariate-adjusted analysis of primary outcomes, if the proportion of subjects with missing values for any covariate is less than 5%, the missing baseline covariates will be imputed using simple imputation methods based on the mean value. If the proportion of subjects with missing values for a covariate is 5%~20%, the missing values will be imputed using Markov chain Monte Carlo (MCMC) methods for multiple imputation approach with 10 imputed copies.

**9.3 Multicenter Studies**

No adjustment on centre will be performed for the primary analysis.

**9.4 Covariates**

In the covariates-adjusted analysis, the following variables will be adjusted:

1) age;

2) sex;

3) time from onset to random assignment;

4) PMI index;

5) baseline e-NIHSS score.

**9.5 Planned Subgroups**

Subgroup analyses could be conducted regardless of whether a significant treatment effect is observed on the primary outcome. The aim of these analyses is to provide additional insights and complement the findings from the primary analysis. These analyses are exploratory in nature and will not lead to formal inferential conclusions about the treatment effect in any particular subgroup. A forest plot will be utilized to depict the treatment effect of adjunctive oral minocycline across each individual subgroup. This plot will illustrate the mean differences, along with its corresponding two-sided 95% confidence intervals (CIs) for each level of the subgroups. Additionally, the adjusted mean differences for each patient characteristic defining a subgroup will be separately presented in the covariates-adjusted analysis. The pre-planned subgroup analyses will investigate the effects of:

1) age (≥ 65 years old vs. < 65 years old)

2) sex (Male vs. Female)

3) time from onset to recanalization. (mins) (≥ median vs. < median)

4) PMI index (0 vs. 1 vs. 2)

5) baseline posterior circulation Alberta Stroke Program Early CT score (pc-ASPECTS) score (≥ 8 vs. <8)

6) baseline e-NIHSS score (≥ median vs. < median)

7) occlusion location (basilar artery vs. V4 segments of vertebral arteries)

8) TOAST etiology (cardioembolic vs. large artery atherosclerosis)

9) intravenous thrombolysis (yes vs. no)

10) patients with symptomatic haemorrhages (yes vs. no).

**9.6 Presentation of Results**

The continuous variables will be described as mean, standard deviation, median, first and third quartiles, and range. For categorical variables, the description will consist of the frequency and percentage. For ordered variables, the description will cover the frequency, percentage, and cumulative percentage.

# **10. Disposition of Subjects and Withdrawals**

The number of patients for each of the following categories will be summarized.

 Total number of assessed patients

 Number of randomized patients

Number of patients completing the study and not completing the study (grouped by treatment and main reason)

 Number of patients included in the ITT population

 Number of patients included in the PP population

Number of patients included in the Safety population

# **11. Demographics and Baseline Characteristics**

Descriptive statistics will be used to summarize clinically important demographic and disease characteristics at screening for each treatment group. These summaries will be generated for both the ITT population and the PP population. If the proportion of missing values exceeds 5%, the denominator used in the calculations will be specified in a footnote of the corresponding summary table.

The variables to be summarized include but are not restricted to the following: Age; Sex; Ethnicity; Body-mass index; Medical History (hypertension, diabetes mellitus, atrial fibrillation, cerebral stroke, alcohol use and smoking); Pre stroke onset mRS score; Baseline NIHSS score; Baseline e-NIHSS score; Baseline pc-ASPECTS Score; Baseline PMI index; Systolic and Diastolic Blood Pressure at hospital arrival; Glucose level at hospital arrival; TOAST etiology; Occlusion Sites; Occlusion site based on DSA; First-line thrombectomy strategy; mTICI score based on DSA run; Intravenous Thrombolysis; Administration of minocycline; Time from stroke onset (or last known well) to randomization, puncture, and recanalization; Time from hospital admission to puncture; Time from groin puncture to recanalization.

# **12. Efficacy Analyses**

**12.1 Primary Efficacy Endpoint (Continuous data)**

The e-NIHSS is a modified NIHSS focused on posterior circulation strokes, with the advantage to improve sensitivity of outcome assessment. The scores of e-NIHSS range from 0 to 42, with higher scores indicating increasing severity. Subjects with missing e-NIHSS score at 5 days will be excluded from the primary analysis.

**12.1.1 Crude and Covariates Adjusted Analysis**

The mean difference and 95% CI of e-NIHSS score at 5 days between two groups will be estimated using a linear mixed model. The mixed model included treatment (intervention vs. control), time (24 hours, 5 days), interaction between treatment and time as fixed effects, baseline measurement as a covariate, and subject as random effect. Normality and variance homogeneity of residuals will be accessed graphically. The win ratio method will be used if normality assumption is seriously violated.

The covariates adjusted mean difference will be calculated by adding the covariates listed in Section 9.4 into the linear mixed model. If the covariate adjusted mixed model does not converge, inverse probability of treatment weighting (IPTW) method will be employed. Both unadjusted and adjusted mean differences at each time point with 95% CIs will be reported.

**12.1.2 Subgroup Analysis**

Subgroup analysis of the e-NIHSS score at 5 days will be performed on the subgroup variables as described in **Section 9.5**. The mean difference and 95% CIs will be estimated for each category of a subgroup variable and displayed in a forest plot to assess exploratorily homogeneity of treatment effect by a subgroup variable. Subgroup analysis will be performed on PP populations.

**12.2 Secondary Efficacy Analysis**

The analyses of the secondary outcomes will be based on the ITT population. Patients with missing data of secondary outcomes will be censored in the primary analysis. Continuous outcomes will be analyzed using linear regression. The win ratio method will be used if normality assumption is seriously violated. Binary outcomes will be analyzed using modified Poisson regression models. Ordinal outcomes will be analyzed using generalized odds ratio (win ratio). The effect parameter of the secondary outcomes will be reported as the risk ratio, win ratio or mean differences as appropriate, along with its 95% CIs. Normality and variance homogeneity of residuals will be accessed graphically.

All analyses will be adjusted by covariates described in **Section 9.4** and reported with 95% CIs. In the covariate-adjusted analysis of secondary outcomes, the missing baseline covariates will be imputed as described in **Section 9.2.2.2.**

For the analysis of the following outcomes, details will outline the definitions and statistical methods that will be used.

**1) Rate of mechanical ventilation**

The rate of mechanical ventilation is defined as the cumulative rate of both invasive and non-invasive ventilation from randomization up to Day 5, regardless of whether the ventilation is due to intraoperative anesthesia or respiratory failure from any cause. The outcomes will be analyzed using modified Poisson regression models. The effect parameter will be reported as the RR and 95% CIs. The analysis will be adjusted by covariates described in **Section 9.4**, and reported with 95% CIs.

**2) Proportion of pneumonia at 5 days**

The diagnosis of pneumonia is made by the treating physician based on the patient’s symptoms, such as a temperature higher than 38 °C or lower than 36 °C, cough, dyspnea, purulent tracheobronchial secretions, leukocytosis > 12,000/mm^3^ or leukopenia <4000/mm^3^, the presence of a new or progressive radiologic pulmonary infiltrate on the chest X-ray or CT, and positive culture from respiratory secretions^[8]^ . The proportion of pneumonia at 5 days will be analyzed using modified Poisson regression models. The effect parameter will be reported as the RR and 95% CIs. The analysis will be adjusted by covariates described in **Section 9.4**, and reported with 95% CIs.

**3)** **Changes in** **lab hematology outcomes at 5 days**

If the proportion of missing values exceeds a predetermined threshold, analysis of lab hematology outcomes will be excluded from the study.

# **13. Safety Analyses**

Safety analyses will be performed exclusively on the Safety population.

**13.1 Primary Safety Analysis (Binary data)**

Proportion of patients with sICH within 24 hours from randomization is considered as the primary safety outcome. sICH is defined as local or remote parenchymal hemorrhage type 2, subarachnoid hemorrhage, and/or intraventricular hemorrhage on the post-treatment imaging scan, combined with a neurological deterioration of 4 points or more on the NIHSS from baseline, or leading to death. Subjects with missing 24 hours CT or MRI scan will be excluded from the primary safety analysis.

The modified Poisson regression models with a treatment group as an independent variable and the rate of sICH within 24 hours from randomization as the dependent variable. The treatment effect will be presented as RR with the corresponding 95% CIs.

**13.2 Secondary Safety Analysis**

Patients with missing data of secondary outcomes will be censored in secondary analysis. Binary outcomes will be analyzed using modified Poisson regression models. The effect parameter of the secondary outcomes will be reported as the RR, along with its 95% CIs.

**13.3 Covariate-Adjusted Analysis**

The covariate-analysis will be adjusted by covariates described in **Section 9.4**, and reported with 95% CI. The missing baseline covariates will be imputed as described in **Section 9.2.2.2.**

**13.4 Adverse Events**

Any unfavorable change in the health status of a trial participant, or the emergence or worsening of any undesirable sign, symptom, or medical condition following enrollment, will be classified as an Adverse Event (AE). All AEs must be documented, regardless of their perceived relationship to the study interventions. Pre-existing medical conditions are excluded from AE reporting.

Considering the temporal association, consistency, intensity, and specificity between AEs and the administration of EVT and/or minocycline treatment, the causality assessment of AEs will be classified into five categories: definitely related; probably related; possibly related; probably unrelated; definitely unrelated. The incidence rate of adverse reactions will be calculated based on the sum of events categorized as definitely related, probably related and possibly related.

The safety of the intervention will be monitored by recording acute adverse events at 24 hours, 5 days, 30 days from randomization and 90 days from randomization as a trial outcome. These events will be monitored periodically by the DSMB and will be captured separately as AEs in the CRF.

**13.5 Serious Adverse Events**

A Serious Adverse Event (SAE) is defined as an untoward occurrence that meets any of the following criteria: results in death, is life-threatening, requires or prolongs hospitalization, or results in persistent or significant disability or incapacity.

Any SAE experienced by a study participant must be reported to the sponsor if the Chief Investigator determines that the event is related to the study procedures or is unexpected. A related SAE is one that arises from the administration of any study intervention. An unexpected SAE is an event that is not listed in the protocol as a foreseeable occurrence.

All deaths occurring during the follow-up period up to Day 90 will be reported as SAEs. When reporting a death, the underlying event or condition that caused or contributed to the fatal outcome should be documented as a single medical concept.

# **14. References**

[1].	STATISTICAL PRINCIPLES FOR CLINICAL TRIALS.

[2].	Ethical Guidelines for Statistical Practice.

[3].	Rinkel, L.A., et al., Comparing Early National Institutes of Health Stroke Scale Versus 90-Day Modified Rankin Scale Outcomes in Acute Ischemic Stroke Trials: A Systematic Review and Analysis. J Am Heart Assoc, 2025. 14(9): p. e040304.

[4].	Olivato, S., et al., e-NIHSS: an Expanded National Institutes of Health Stroke Scale Weighted for Anterior and Posterior Circulation Strokes. J Stroke Cerebrovasc Dis, 2016. 25(12): p. 2953-2957.

[5].	Tao, C., et al., Trial of Endovascular Treatment of Acute Basilar-Artery Occlusion. N Engl J Med, 2022. 387(15): p. 1361-1372.

[6].	Hozo, S.P., B. Djulbegovic and I. Hozo, Estimating the mean and variance from the median, range, and the size of a sample. BMC Med Res Methodol, 2005. 5: p. 13.

[7].	Molero-Calafell, J., et al., Intention to treat and per protocol analyses: differences and similarities. J Clin Epidemiol, 2024. 173: p. 111457.

[8].	Badve, M.S., et al., Frequency of post-stroke pneumonia: Systematic review and meta-analysis of observational studies. Int J Stroke, 2019. 14(2): p. 125-136.

**MIST-B SAP summary of changes**

**SAP Version 1.0 date:** 27th June 2022

**SAP Version 2.0 date:** 22th November 2022

**SAP Version 3.0 date:** 12th February 2025

| **Sections** | **SAP Version 1.0** | **SAP Version 2.0** | **SAP Version 3.0** | **Rationale** |
| --- | --- | --- | --- | --- |
| 4.1 Primary Efficacy and Safety Endpoints | proportion of modified Rankin Scale (mRS) 0 to 3 at 90 days from randomization | ***Changed:***  expanded National Institutes of Health Stroke Scale (e-NIHSS) scores at 5 days |  | To shorten the required follow-up duration, minimize interference from confounding factors, allow for a smaller sample size, and enhance the sensitivity of outcome assessment posterior circulation strokes. |
| 4.2 Secondary Efficacy and Safety Endpoints | proportion of modified Thrombolysis In Cerebral Infarction(mTICI) 2b-3 after endovascular treatment. | ~~proportion of modified Thrombolysis In Cerebral Infarction(mTICI) 2b-3 after endovascular treatment.~~  ***Added:***  proportion of modified Rankin Scale (mRS) 0 to 3 at 90 days from randomization |  | To be consistent with the Change of Inclusion Criteria and Primary Efficacy Endpoints |
| 5. Overall Study Design and Plan | MIST-B is an evaluator-blinded, randomized, open-label, proof of concept trial.  The study has a planned duration of 15 months to recruit 40 patients in China. | ***Changed:***  MIST-B is a multicenter, prospective, randomized, open-label clinical trial with blinded outcome assessment. The study has a planned duration of 2 years to recruit 90 patients across 12 comprehensive stroke centers in China. | ***Changed:***  The study has a planned duration of 2.5 years to recruit 90 patients across 12 comprehensive stroke centers in China. | To increase the reliability of the research results |
| 7. Sample Size Determination | Considering the nature of exploring study in our trail, we plan to enroll 40 patients (20 patients in each group). | ***Changed:***  We use the NIHSS parameters in previous studies to estimate the sample size of this study. Therefore, we calculated that a sample size of **90 patients (45 patients in each group with a dropout rate of 10%)** would detect a 3 points difference (16±5.3 for the control group and 13±5.3 for the intervention group) in e-NIHSS scores of patients at 5 days, with a power of 80% and two-sided significance levels of 0.05 for the analysis of the primary effectiveness outcome. Sample size was computed using PASS 11.0.7. |  | To improve the rigor of the trial: We based our estimations on the results of the ATTENTION trial. The means (SD) of NIHSS scores in patients with thrombectomy at 5 days is calculated as 16±5.3 by the methods of Iztok Hozo. Due to the lack of previous data on adjunctive minocycline in addition to EVT, it is considered a reasonable prediction that NIHSS scores in adjunctive minocycline group would be 3 points lower than those in control group at 5 days, based on the discussion by the Academic Committee and the Executive Committee. |
| 9.2.2.2 Variables in covariate-adjusted analysis |  | ***Added:***  In the covariate-adjusted analysis of primary outcomes, if the proportion of subjects with missing values for any covariate is less than 5%, the missing baseline covariates will be imputed using simple imputation methods based on the mean value. If the proportion of subjects with missing values for a covariate is 5%~20%, the missing values will be imputed using Markov chain Monte Carlo (MCMC) methods for multiple imputation approach with 10 imputed copies. |  | To amend the analysis method of the primary outcomes. |
| 9.3 Multicenter Studies |  | ***Added:***  No adjustment on centre will be performed for the primary analysis. |  | To be consistent with the Change of analysis method of the primary analysis, and the Change of overall study design. |
| 9.4 Covariates |  | ***Added:***  The following variables will be adjusted: 1) age; 2) sex; 3) time from onset to random assignment; 4) PMI index; 5) baseline e-NIHSS score. |  | To amend the analysis method of the primary outcomes. |
| 9.5 Planned Subgroups |  | ***Added:***  Subgroup analyses could be conducted regardless of whether a significant treatment effect is observed on the primary outcome. The aim of these analyses is to provide additional insights and complement the findings from the primary analysis. These analyses are exploratory in nature and will not lead to formal inferential conclusions about the treatment effect in any particular subgroup. A forest plot will be utilized to depict the treatment effect of adjunctive oral minocycline across each individual subgroup. This plot will illustrate the mean differences, along with its corresponding two-sided 95% confidence intervals (CIs) for each level of the subgroups. Additionally, the adjusted mean differences for each patient characteristic defining a subgroup will be separately presented in the covariates-adjusted analysis. The pre-planned subgroup analyses will investigate the effects of: 1) age (≥ 65 years old vs. < 65 years old); 2) sex (Male vs. Female); 3) time from onset to recanalization. (mins) (≥ median vs. < median); 4) PMI index (0 vs. 1 vs. 2); 5) baseline pc-ASPECTS score (≥ 8 vs. <8); 6) baseline e-NIHSS score (≥ median vs. < median); 7) occlusion location (basilar artery vs. V4 segments of vertebral arteries); 8) TOAST etiology (cardioembolic vs. large artery atherosclerosis); 9) intravenous thrombolysis (yes vs. no); 10) patients with symptomatic haemorrhages (yes vs. no). |  | To amend the analysis method of the efficacy and safety outcomes. |
| 12. Efficacy Analyses | Continuous outcomes will be analyzed using linear regression or generalized linear models as appropriate. Categorical outcomes will be analyzed using modified poissson regression models. Ordinal outcomes will be analyzed using multivariable ordinal logistic regression. | ***Changed:***  12.1 Primary Efficacy Endpoint  The e-NIHSS is a modified NIHSS focused on posterior circulation strokes, with the advantage to improve sensitivity of outcome assessment. The scores of e-NIHSS range from 0 to 42, with higher scores indicating increasing severity. Subjects with missing e-NIHSS score at 5 days will be excluded from the primary analysis.  ***Added:***  12.1.1 Crude and Covariates Adjusted Analysis  The mean difference and 95% CI of e-NIHSS score at 5 days between two groups will be estimated using a linear mixed model. The mixed model included treatment (intervention vs. control), time (24 hours, 5 days), interaction between treatment and time as fixed effects, baseline measurement as a covariate, and subject as random effect. Normality and variance homogeneity of residuals will be accessed graphically. The win ratio method will be used if normality assumption is seriously violated.  The covariates adjusted mean difference will be calculated by adding the covariates listed in Section 9.4 into the linear mixed model. If the covariate adjusted mixed model does not converge, inverse probability of treatment weighting (IPTW) method will be employed. Both unadjusted and adjusted mean differences at each time point with 95% CIs will be reported.  12.1.2 Subgroup Analysis  Subgroup analysis of the e-NIHSS score at 5 days will be performed on the subgroup variables as described in Section 9.5. The mean difference and 95% CIs will be estimated for each category of a subgroup variable and displayed in a forest plot to assess exploratorily homogeneity of treatment effect by a subgroup variable. Subgroup analysis will be performed on PP populations. |  | To amend the analysis method of the efficacy and safety outcomes. |
| 12.2 Secondary Efficacy Analysis | Continuous outcomes will be analyzed using linear regression or generalized linear models as appropriate. Categorical outcomes will be analyzed using modified Poisson regression models. Ordinal outcomes will be analyzed using multivariable ordinal logistic regression. | ***Added:***  12.2 Secondary Efficacy Analysis  The analyses of the secondary outcomes will be based on the ITT population. Patients with missing data of secondary outcomes will be censored in the primary analysis. Continuous outcomes will be analyzed using linear regression. The win ratio method will be used if normality assumption is seriously violated. Binary outcomes will be analyzed using modified Poisson regression models. Ordinal outcomes will be analyzed using generalized odds ratio (win ratio). The effect parameter of the secondary outcomes will be reported as the risk ratio, wins ratio or mean differences as appropriate, along with its 95% CIs. Normality and variance homogeneity of residuals will be accessed graphically.  All analyses will be adjusted by covariates described in Section 9.4 and reported with 95% CIs. In the covariate-adjusted analysis of secondary outcomes, the missing baseline covariates will be imputed as described in Section 9.2.2.2.  For the analysis of the following outcomes, details will outline the definitions and statistical methods that will be used.  1) Rate of mechanical ventilation; 2) Proportion of pneumonia at 5 days; 3) Changes in lab hematology outcomes at 5 days. |  | To amend the analysis method of the efficacy and safety outcomes. |
| 13. Safety Analyses | Safety analyses will be performed exclusively on the Safety population. Categorical outcomes will be analyzed using modified Poisson regression models. The effect parameter of the secondary outcomes will be reported as the RR, along with its 95% CIs. | ***Added***  13.1 Primary Safety Analysis (Binary data)  Proportion of patients with sICH within 24 hours from randomization is considered as the primary safety outcome. sICH is defined as local or remote parenchymal hemorrhage type 2, subarachnoid hemorrhage, and/or intraventricular hemorrhage on the post-treatment imaging scan, combined with a neurological deterioration of 4 points or more on the NIHSS from baseline, or leading to death. Subjects with missing 24 hours CT or MRI scan will be excluded from the primary safety analysis.  The modified Poisson regression models with a treatment group as an independent variable and the rate of sICH within 24 hours from randomization as the dependent variable. The treatment effect will be presented as RR with the corresponding 95% CIs.  13.2 Secondary Safety Analysis  Patients with missing data of secondary outcomes will be censored in secondary analysis. Binary outcomes will be analyzed using modified Poisson regression models. The effect parameter of the secondary outcomes will be reported as the RR, along with its 95% CIs.  13.3 Covariate-Adjusted Analysis  The covariate-analysis will be adjusted by covariates described in Section 9.4, and reported with 95% CI. The missing baseline covariates will be imputed as described in Section 9.2.2.2. |  | To amend the analysis method of the efficacy and safety outcomes. |
