# Supplementary material for: Minocycline for acute basilar artery occlusion stroke undergoing endovascular treatment: a randomized, evaluator-blinded, open-label, pilot trial
Source: BMC Med. 2026 Jul 27;24:580. doi: 10.1186/s12916-026-05092-4 (PMC13688725; doi:10.1186/s12916-026-05092-4)
Supplement: Supplementary file 3 [file 12916_2026_5092_MOESM3_ESM.docx]

**Additional Tables and Figures**

**Minocycline for acute basilar artery occlusion stroke undergoing endovascular treatment:** **a randomized, evaluator-blinded, open-label, pilot trial**

**CONTENTS**

**Table S1.** Inclusion and Exclusion Criteria.

**Table S2.** Trial Schedule.

**Table S3.** Enrollment Progress at Each Center.

**Table S4.** Further Baseline Characteristics (modified Intention-to-Treat Population).

**Table S5.** Baseline Characteristics of Study Participants (per-protocol Population)

**Table S6.** Primary and secondary outcomes by treatment (per-protocol Population)

**Table S7.** Further Secondary Outcomes by Treatment (modified Intention-to-Treat Population).

**Table S8.** Adverse Events of The Participants (Safety Population).

**Table S9**. Details of Adverse Events Occurring Within the Safety Population.

**Table S10.** Details of Severe Adverse Events Occurring Within the Safety Population.

**Fig. S1** Enrollment Progress of MIST-B Trial over Trial Period.

**Fig. S2** Distribution of mRS score at day 90 by treatment (modified Intention-to-Treat Population).

**Table S1.** Inclusion and Exclusion Criteria

Inclusion criteria

- Age ≥ 18 years old.
- Patients had acute symptoms and signs compatible with acute ischemia stroke due to basilar artery occlusion (BAO) or intracranial segments of vertebral arteries (VA) resulting in no flow to the basilar artery (eg, functional basilar artery occlusion);
- Last known well to groin puncture between 0 to 24 hours, whether or not patients had thrombolysis with rt-PA;
- Pre-stroke mRS score of 0-1;
- Baseline expanded NIHSS (e-NIHSS) score ≥ 6;
- Signed Informed Consent obtained;
- Neuroimaging Inclusion Criteria: (1) Proven large vessel occlusion in BAO or VA-V4 occlusion (mTICI score 0-1) determined by CTA or MRA; (2) pc-ASPECTS score ≥ 5 (Non-Contrast CT or DWI); (3) Pons-midbrain-index of <3.

Exclusion criteria

- Age <18 years old;
- Complete cerebellar infarct with significant mass effect or has the imaging features of acute hydrocephalus in NCCT;
- Intracranial hemorrhage at baseline assessment;
- Previous stroke in the past 90 days;
- Cardiopulmonary resuscitation was performed within 10 days prior to onset;
- Known hereditary or acquired hemorrhagic diathesis, coagulation factor deficiency, international normalized ratio >3, or platelet <40×109/L;
- Glucose <2.2 mmol/L or >22 mmol/L;
- Systolic blood pressure persistently >185mmHg or diastolic blood pressure persistently >110mmHg despite antihypertensive intervention;
- Acute or chronic renal failure of CKD grade 3-4;
- Known allergy or hypersensitivity to contrast dye or tetracycline group of drugs;
- Epileptic seizure at symptom onset;
- Life expectancy (except for stroke) < 3 months;
- Female who is pregnancy or breastfeeding, or whom do not use effective contraception at childbearing age;
- Pre-existing mental illness that interferes with neurological evaluation;
- Known current participation in another clinical investigation with experimental drug;
- Unlikely to be available for 90 days follow-up.

**Table S2.** Trial Schedule

| Visit | Study Period | | | | | |
| --- | --- | --- | --- | --- | --- | --- |
|  | 0 | 1 | 2 | 3 | 4 | 5 |
|  | Screening | Allocation and Intervention | In-hospital follow-up | | Close-out | |
| Timepoints | 0 | 0 | 1 d | 5 d | 30 d  (+/-7 d) | 90 d  (+/-14 d) |
| **Enrollment:** |  |  |  |  |  |  |
| In/Exclusion criteria | X |  |  |  |  |  |
| Informed consent | X |  |  |  |  |  |
| Randomization | X |  |  |  |  |  |
| **Assessments:** |  |  |  |  |  |  |
| Demographic characteristics | X |  | X | X | X | X |
| Medical history | X |  |  | X |  | X |
| Vital signs | X |  | X | X | X | X |
| Physical examinations | X |  | X | X | X | X |
| Clinical scores (NIHSS, e-NIHSS, mBI, and mRS) ^a^ | X |  | X | X | X | X |
| Laboratory tests | X |  | X | X | X | X |
| MRI head ^b^ | X |  |  | X |  | X |
| NCCT head ^c^ | X |  | X |  |  |  |
| MRA or CTA head | X |  | X |  | X | X |
| AE and SAE |  | X | X | X | X | X |
| **Treatment:** |  |  |  |  |  |  |
| DSA and endovascular treatment |  | X |  |  |  |  |
| Minocycline treatment |  | X | X | X |  |  |
| Other Medical treatment | X | X | X | X | X | X |

Abbreviations: NIHSS, National Institutes of Health Stroke Scale; e-NIHSS, expanded National Institutes of Health Stroke Scale; mBI, modified Barthel Index score; mRS, modified Rankin Scale; MRI, magnetic resonance imaging; DWI, diffusion-weighted imaging; NCCT, non-contrast cranial computed tomography; MRA, magnetic resonance angiography; CTA, computed tomographic angiography; DSA, digital subtraction angiography; AE, adverse event; SAE, severe adverse event.

^a^ At 30 days and 90 days, a face-to-face visit is preferred. If a face-to-face visit is not possible, they can be contacted by telephone for assessment of mBI and mRS. Assessment needs to be performed by an independent evaluator blinded to treatment assignment.

^b^ DWI of MRI head is preferable at baseline assessment but can be substituted by NCCT of the head if DWI is unavailable or contraindicated. FLAIR of MRI should be done to determine changes in infarct volume if patients were in stable condition at 5 days.

^b^ NCCT is repeated to determine intracranial hemorrhages at 24 hours after randomization, or anytime a neurological deterioration on the NIHSS.

^c^ At 30 days and 90 days, a face-to-face visit is preferred. If a face-to-face visit is not possible, they can be contacted by telephone for assessment of mBI and mRS. Assessment needs to be performed by an independent evaluator blinded to treatment assignment.

**Table S3.** Enrollment Progress at Each Center

| Center | Total screened | Total enrolled | Date of first patient-in | Date of last patient-in |
| --- | --- | --- | --- | --- |
| All centers | 242 | 90 | 4.3.2023 | 4.3.2025 |
| Xijing Hospital, Fourth Military Medical University | 62 | 20 | 4.3.2023 | 4.3.2025 |
| Xi'an XD Group Hospital | 27 | 13 | 10.4.2023 | 2.3.2025 |
| Baoji High-tech Hospital | 20 | 12 | 17.10.2023 | 9.9.2024 |
| Gansu Provincial Maternity and Child-care Hospital (Gansu Provincial Central Hospital) | 17 | 12 | 16.6.2023 | 5.1.2025 |
| Xi'an Daxing Hospital | 44 | 11 | 3.4.2023 | 24.10.2024 |
| The First People's Hospital of Xianyang | 42 | 10 | 28.9.2023 | 4.3.2025 |
| Weinan Central Hospital | 11 | 6 | 8.3.2024 | 29.12.2024 |
| Xi'an Gaoxin Hospital | 7 | 2 | 3.4.2024 | 3.4.2024 |
| Xianyang Hospital of Yan'an University | 3 | 1 | 20.7.2023 | 20.7.2023 |
| Yan'an People's Hospital | 4 | 1 | 19.5.2024 | 19.5.2024 |
| The First Hospital of Yulin | 3 | 1 | 23.1.2024 | 23.1.2024 |
| Yulin Hospital, The First Affiliated Hospital of Xi'an Jiaotong University | 2 | 1 | 24.12.2023 | 24.12.2023 |

**Table S4.** Further Baseline Characteristics (modified Intention-to-Treat Population)

| Characteristics | Minocycline group  (n=45) | Control group  (n=44) |
| --- | --- | --- |
| Ethnicity, Han Chinese population, n/N (%) | 44/45 (97.8) | 44/44 (100) |
| Occlusion site based on first DSA run, n/N (%) |  |  |
| V1-V3 segments of vertebral arteries | 3/45 (6.7) | 1/44 (2.3) |
| V4 segments of vertebral arteries | 10/45 (22.2) | 12/44 (27.3) |
| Proximal basilar artery | 14/45 (31.1) | 14/44 (31.8) |
| Middle basilar artery | 10/45 (22.2) | 9/44 (20.5) |
| Distal basilar artery | 7/45 (15.6) | 8/44 (18.2) |
| First-line thrombectomy strategy ^a^, n/N (%) |  |  |
| Stent retriever only | 1/45 (2.2) | 7/44 (15.9) |
| Aspiration only | 8/45 (17.8) | 8/44 (18.2) |
| Intra-arterial thrombolysis only | 1/45 (2.2) | 0/44 (0.0) |
| Rescue with stent or PTA | 3/45 (6.7) | 3/44 (6.8) |
| Primary combined approach | 32/45 (71.1) | 25/44 (56.8) |
| Original mTICI score based on first DSA run, n/N (%) |  |  |
| mTICI 0 | 39/45 (86.7) | 40/44 (90.9) |
| mTICI 1 | 5/45 (11.1) | 4/44 (9.1) |
| mTICI 2-3 | 1/45 (2.2) | 0/44 (0.0) |
| Final mTICI score based on last DSA run, n/N (%) |  |  |
| mTICI 0-2a | 1/45 (2.2) | 4/44 (9.1) |
| mTICI 2b-3 | 44/45 (97.7) | 40/44 (90.9) |
| Workflow times |  |  |
| From hospital admission to puncture | 110 (69, 181) | 101 (70, 142) |
| From groin puncture to recanalization | 97 (60, 120) | 72 (38, 104) |
| Administration of minocycline |  |  |
| before arterial puncture | 38 | NA |
| within 1 hour after EVT | 7 | NA |
| Tirofiban during perioperative period | 34/45 (75.6) | 32/44 (72.7) |
| Baseline pc-ASPECTS score determined by ^b^, n/N (%) |  |  |
| DWI | 37/45 (82.2) | 35/44 (79.5) |
| NCCT | 8/45 (17.8) | 9/44 (20.5) |

Abbreviations: PTA, percutaneous transluminal angioplasty; DSA, digital subtraction angiography; mTICI, modified Thrombolysis In Cerebral Infarction.

^a^ The choice of EVT technique was left to the discretion of the treating physician. On the basis of assessment, 1 patient in the minocycline group were excluded from the analyses due to only underwent intraarterial lysis.

^b^ pc-ASPECTS is a screening tool for endovascular treatment in patients with acute BAO. Scores range from 0 to 10, with higher scores indicating a smaller infarct core. The scores were determined by diffusion-weighted imaging or non-contrast cranial computed tomography.

**Table S5.** Baseline Characteristics of Study Participants (per-protocol Population)

| Characteristics | Minocycline group  (n=42) | Control group  (n=44) |  |
| --- | --- | --- | --- |
| Age, mean (SD), yr | 62.1 (11.6) | 61.4 (12.1) |  |
| Female, n/N (%) | 13/42 (31.0) | 14/44 (31.8) |  |
| Body-mass index, kg/m^2^ | 24.5 (22.9, 25.4) | 23.5 (22.2, 25.7) |  |
| Medical history ^a^, n/N (%) |  |  |  |
| Hypertension | 30/42 (71.4) | 30/44 (68.2) |  |
| Atrial fibrillation | 4/42 (9.5) | 4/44 (9.1) |  |
| Diabetes | 11/42 (26.2) | 11/44 (25) |  |
| Cerebral stroke | 3/42 (7.1) | 7/44 (15.9) |  |
| Smoking | 15/42 (35.7) | 16/44 (36.4) |  |
| Alcohol use | 8/42 (19.0) | 8/44 (18.2) |  |
| Pre-stroke mRS score, median (IQR) | 0 (0, 0) | 0 (0, 0) |  |
| Disease severity |  |  |  |
| NIHSS score, median (IQR) | 18.0 (10.0, 35.0) | 19.5 (10.5, 35.0) |  |
| e-NIHSS score, median (IQR) | 19.5 (12.0, 35.0) | 20.5 (11.0, 35.0) |  |
| pc-ASPECTS ^b^, median (IQR) | 7.0 (6.0, 8.0) | 8.0 (6.0, 8.0) |  |
| PMI index ^b^, median (IQR) | 1.0 (0.0, 2.0) | 1.0 (0.0, 2.0) |  |
| TOAST etiology, n/N (%) |  |  |  |
| Cardioembolic | 7/42 (16.7) | 7/44 (15.9) |  |
| Large artery atherosclerosis | 33/42 (78.6) | 35/44 (79.5) |  |
| Others | 0/42 (0.0) | 1/44 (2.3) |  |
| Unknown | 2/42 (4.8) | 1/44 (2.3) |  |
| Occlusion site based on baseline image ^c^, n/N (%) |  |  |  |
| V4 segments of vertebral arteries | 16 (38.1) | 17 (38.6) |  |
| Basilar artery | 26 (61.9) | 27 (61.4) |  |
| Intravenous thrombolysis, n/N (%) | 10/42 (23.8) | 15/44 (34.1) |  |
| Time from stroke onset, median (IQR), mins |  |  |  |
| To randomization | 430 (299, 867) | 344 (163, 684) |  |
| To puncture | 465 (362, 908) | 386 (185, 727) |  |
| To recanalization ^d^ | 588 (439, 1060) | 476 (275, 827) |  |
| Blood glucose, median (IQR), mmol/L | 8.0 (6.8, 10.1) | 8.2 (6.5, 10.3) |  |
| SBP, median (IQR), mm Hg | 148 (134, 167) | 140 (131, 152) |  |
| DBP, median (IQR), mm Hg | 85 (77, 96) | 82 (76, 90) |  |
| Ethnicity, Han Chinese population, n/N (%) | 41/42 (97.6) | 44/44 (100) |  |
| Occlusion site based on first DSA run, n/N (%) |  |  |  |
| V1-V3 segments of vertebral arteries | 3/42 (7.1) | 1/44 (2.3) |  |
| V4 segments of vertebral arteries | 10/42 (23.8) | 12/44 (27.3) |  |
| Proximal basilar artery | 13/42 (31.0) | 14/44 (31.8) |  |
| Middle basilar artery | 10/42 (23.8) | 9/44 (20.5) |  |
| Distal basilar artery | 6/42 (14.3) | 8/44 (18.2) |  |
| First-line thrombectomy strategy ^e^, n/N (%) |  |  |  |
| Stent retriever only | 1/42 (2.4) | 7/44 (15.9) |  |
| Aspiration only | 7/42 (16.7) | 8/44 (18.2) |  |
| Rescue with stent or PTA | 3/42 (7.1) | 3/44 (6.8) |  |
| Primary combined approach | 31/42 (73.8) | 25/44 (56.8) |  |
| Original mTICI score based on first DSA run, n/N (%) |  |  |  |
| mTICI 0 | 38/42 (90.5) | 40/44 (90.9) |  |
| mTICI 1 | 4/42 (9.5) | 4/44 (9.1) |  |
| Final mTICI score based on last DSA run, n/N (%) |  |  |  |
| mTICI 0-2a | 1/42 (2.4) | 4/44 (9.1) |  |
| mTICI 2b-3 | 41/42 (97.6) | 40/44 (90.1) |  |
| Workflow times |  |  |  |
| From hospital admission to puncture | 110 (72, 186) | 101 (70, 142) |  |
| From groin puncture to recanalization | 95 (63, 120) | 72 (38, 104) |  |
| Administration of minocycline |  |  |  |
| before arterial puncture | 35 | NA |  |
| within 1 hour after EVT | 7 | NA |  |
| Tirofiban during perioperative period | 33/42 (78.6) | 32/44 (72.7) |  |
| Baseline pc-ASPECTS score determined by ^f^, n/N (%) |  |  |  |
| DWI | 34/42 (81.0) | 35/44 (79.5) |  |
| NCCT | 8/42 (19.0) | 9/44 (20.5) |  |

Abbreviations: CTA, computed tomography angiography; DBP, diastolic blood pressure; e-NIHSS, expanded National Institutes of Health Stroke Scale; mRS, modified Rankin Scale; MRA, magnetic resonance angiography; NIHSS, National Institutes of Health Stroke Scale; pc-ASPECTS, posterior circulation Alberta Stroke Program Early Computed Tomography Score; PMI, Pons-midbrain-index; SBP, systolic blood pressure; TOAST, Trial of Org 10172 in Acute Stroke Treatment; PTA, percutaneous transluminal angioplasty; DSA, digital subtraction angiography; mTICI, modified Thrombolysis In Cerebral Infarction.

^a^ Comorbidities based on family or patient report.

^b^ Values of pc-ASPECTS and PMI were determined by diffusion-weighted imaging or non-contrast cranial computed tomography at baseline assessment. On the basis of assessment, 1 patient in the minocycline group were excluded from the analyses due to only 1 point of pc-ASPECTS.

^c^ Values of occlusion location were determined by CTA or MRA at baseline assessment. Images were lost or unable to complete CTA/MRA examination for 5 patients in the minocycline group and for 2 in the control group. For these 7 patients, the occlusion of V4 segments of vertebral arteries or basilar artery were confirmed by digital subtraction angiography. On the basis of assessment, 1 patient in the minocycline group were excluded from the analyses due to no occlusion of basilar artery or V4 segments of vertebral arteries.

^d^ Recanalization was defined as the first visualization of successful reperfusion, as indicated by a modified Thrombolysis in Cerebral Infarction (mTICI) grade of 2b or 3. Data on the time from stroke onset to recanalization were missing for 1 patient in the minocycline group and 4 in the control group, due to unsuccessful recanalization.

^e^ The choice of EVT technique was left to the discretion of the treating physician. On the basis of assessment, 1 patient in the minocycline group were excluded from the analyses due to only underwent intraarterial lysis.

^f^ pc-ASPECTS is a screening tool for endovascular treatment in patients with acute BAO. Scores range from 0 to 10, with higher scores indicating a smaller infarct core. The scores were determined by diffusion-weighted imaging or non-contrast cranial computed tomography.

**Table S6.** Primary and secondary outcomes by treatment (per-protocol Population)

| Outcome | Minocycline group  (n=42) | Control group  (n=44) | Measure of effect | Unadjusted value  (95% CI) ^a^ | Adjusted value  (95% CI) ^b^ |
| --- | --- | --- | --- | --- | --- |
| e-NIHSS score at 5 d (**primary outcome**), no, Mean (SD) ^c^ | 42, 15.0 (13.5) | 44, 17.2 (16.9) | Mean difference | -1.82 (-7.23, 3.58) | -2.71 (-7.85, 2.43) |
| e-NIHSS score at 24 h, no, Mean (SD) ^c^ | 42, 18.2 (13.9) | 44, 20.8 (15.2) | Mean difference | -2.15 (-7.56, 3.25) | -3.04 (-8.18, 2.10) |
| mRS score at 90 d, No. of wins/total No. of pairs ^d^ | 810/1806 (44.9%) | 637/1806 (35.3%) | Win ratio | 1.27 (0.70, 2.30) | 1.32 (0.71, 2.51) |
| mRS score of 0-1 at 90 d, n/N (%) ^e^ | 18/42 (42.9) | 16/43 (36.4) | Risk ratio | 1.15 (0.68, 1.95) | 1.24 (0.75, 2.05) |
| mRS score of 0-2 at 90 d, n/N (%) ^e^ | 22/42 (52.4) | 20/43 (45.5) | Risk ratio | 1.13 (0.73, 1.74) | 1.23 (0.83, 1.83) |
| mRS score of 0-3 at 90 d, n/N (%) ^e^ | 24/42 (57.1) | 23/43 (52.7) | Risk ratio | 1.07 (0.73, 1.57) | 1.18 (0.83, 1.66) |
| NIHSS score at 24 h, no, Mean (SD) ^c^ | 42, 17.5 (14.2) | 44, 20.4 (15.4) | Mean difference | -2.41 (-7.89, 3.07) | -3.34 (-8.56, 1.89) |
| NIHSS score at 5 d, no, Mean (SD) ^c^ | 42, 14.3 (13.6) | 44, 16.9 (16.9) | Mean difference | -2.12 (-7.61, 3.36) | -3.05 (-8.28, 2.17) |
| mBI scores at 90 d, No. of wins/total No. of pairs ^d^ | 701/1806 (38.8%) | 554/1806 (30.7%) | Win ratio | 1.27 (0.66, 2.44) | 1.29 (0.65, 2.65) |
| Length of ICU stay, day, no, Median (IQR) ^c^ | 42, 5.0 (2.0, 7.0) | 44, 5.0 (2.8, 7.0) | GMR | 1.02 (0.74, 1.40) | 0.65 (0.19, 2.22) |
| Length of hospital stay, day, no, Median (IQR) ^c^ | 42, 9.5 (6.0, 13.8) | 44, 8.5 (5.0, 12.0) | GMR | 1.13 (0.81, 1.57) | 1.13 (0.83, 1.55) |
| Mechanical ventilation within 5 days, n/N (%) ^e^ | 14/42 (33.3) | 21/44 (47.7) | Risk ratio | 0.70 (0.41, 1.19) | 0.70 (0.44, 1.13) |
| Pneumonia within 5 days, n/N (%) ^e^ | 25/42 (59.5) | 31/44 (70.5) | Risk ratio | 0.84 (0.61, 1.16) | 0.83 (0.61, 1.12) |
| Leucocyte counts at 5 days, ×10^9^/L, no, Median (IQR) ^c^ | 35, 8.4 (6.8, 9.9) | 32, 9.0 (6.9, 10.6) | GMR | 0.98 (0.82, 1.17) | 0.97 (0.80, 1.18) |
| Percentage of neutrophilic granulocyte at 5 days, %, No. of wins/total No. of pairs ^d^ | 35, 602/1120 (53.8%) | 32, 517/1120 (46.2%) | Win ratio | 1.16 (0.67, 2.03) | 1.02 (0.55, 1.93) |
| Absolute neutrophil value at 5 days, 10^9^/L, No. of wins/total No. of pairs ^d^ | 35, 555/1120 (49.6%) | 32, 558/1120 (49.8%) | Win ratio | 0.99 (0.58, 1.71) | 1.17 (0.67, 2.19) |

^a^ Analyses of efficacy outcomes and safety outcomes were conducted in per-protocol population.

^b^ Covariates adjusted included age, sex, time from onset to random assignment, PMI index, baseline e-NIHSS score, and intravenous thrombolysis. If a model with covariates added did not converge, IPTW approach was used.

^c^ The linear mixed model was used, which included treatment, time, interaction between treatment and time as fixed effects, baseline measurement as a covariate, and subject as random effect, from which treatment difference with 95%CI at each time was calculated. Normality was assessed using Q-Q plot.

^d^ No. of wins/total No. of pairs (%), win ratio (generalized odds ratio) were calculated using WINS package.

^e^ Unadjusted and adjusted risk ratio were calculated using modified Poisson regression model.

Abbreviations: e-NIHSS, expanded National Institutes of Health Stroke Scale; mRS, modified Rankin Scale; mBI, modified Barthel Index score; NIHSS, National Institutes of Health Stroke Scale.

**Table S7.** Further Secondary Outcomes by Treatment (modified Intention-to-Treat Population)

| Outcomes | Minocycline group  (n=45) | Control group  (n=44) | Measure of effect | Unadjusted value  (95% CI) ^a^ | Adjusted value  (95% CI) ^b^ |
| --- | --- | --- | --- | --- | --- |
| Length of ICU stay, day, no, Median (IQR) ^c^ | 45, 5.0 (2.0, 7.0) | 44, 5.0 (2.8, 7.0) | GMR | 0.74 (0.23, 2.41) | 0.73 (0.22, 2.42) |
| Length of hospital stay, day, no, Median (IQR) ^c^ | 45, 10.0 (6.0, 14.0) | 44, 8.5 (5.0, 12.0) | GMR | 1.20 (0.86, 1.68) | 1.21 (0.88, 1.68) |
| Mechanical ventilation within 5 days, n/N (%) ^d^ | 16/45 (35.6) | 21/44 (47.7) | Risk ratio | 0.74 (0.45, 1.23) | 0.78 (0.49, 1.22) |
| Pneumonia within 5 days, n/N (%) ^d^ | 26/45 (57.8) | 31/44 (70.5) | Risk ratio | 0.82 (0.60, 1.12) | 0.81 (0.59, 1.11) |
| Leucocyte counts at 5 days, ×10^9^/L, no, Median (IQR) ^c^ | 36, 8.4 (6.9, 9.6) | 32, 9.0 (6.9, 10.6) | GMR | 0.99 (0.82, 1.19) | 0.97 (0.80, 1.17) |
| Percentage of neutrophilic granulocyte at 5 days, %, No. of wins/total No. of pairs ^e^ | 36, 578/1152 (50.2%) | 32, 567/1152 (46.2%) | Win ratio | 1.02 (0.57, 1.74) | 1.05 (0.58, 1.81) |
| Absolute neutrophil value at 5 days, 10^9^/L, No. of wins/total No. of pairs ^e^ | 36, 622/1152 (54.0%) | 32, 529/1152 (49.8%) | Win ratio | 1.18 (0.67, 2.20) | 1.18 (0.67, 2.19) |

^a^ Analyses of efficacy outcomes were conducted in modified intention-to-treat population.

^b^ Covariates adjusted included age, sex, time from onset to random assignment, PMI index, baseline e-NIHSS score, and intravenous thrombolysis. If a model with covariates added did not converge, IPTW approach was used.

^c^ The linear regression model with treatment the study variable and baseline measurement as covariate was employed. Normality was assessed using Q-Q plot.

^d^ Unadjusted and adjusted risk ratio were calculated using modified Poisson regression model.

^e^ No. of wins/total No. of pairs (%), win ratio (generalized odds ratio) were calculated using WINS package.

Abbreviations: ICU, intensive care unit; GMR, geometric mean ratio.

**Table S8.** Adverse Events of The Participants (Safety Population)

| Characteristics | Minocycline group  (n=43) | Control group  (n=43) |
| --- | --- | --- |
| Patients with AEs at 90 d, No. (%) | 28 (65) | 35 (81) |
| Patients with SAEs at 90 d, No. (%) | 16 (37) | 18 (42) |
| Any AEs, No. (%) |  |  |
| BUN/sCr elevation | 2 (4.7) | 3 (7.0) |
| Liver enzyme elevation | 2 (4.7) | 7 (16) |
| Thrombocytopenia | 1 (2.3) | 1 (2.3) |
| Anemia | 3 (7.0) | 5 (12) |
| Abnormal coagulation | 0 (0.0) | 3 (7.0) |
| Pneumonia | 26 (60) | 31 (72) |
| Gut infection | 1 (2.3) | 0 (0.0) |
| Puncture site hematoma | 1 (2.3) | 1 (2.3) |
| Pseudoaneurysm | 1 (2.3) | 1 (2.3) |
| Any SAEs, No. (%) |  |  |
| sICH | 3 (7.3) | 6 (15) |
| Mortality | 10 (23) | 14 (33) |
| Circulation failure | 2 (4.7) | 2 (4.7) |
| Ischemic heart disease | 0 (0.0) | 1 (2.3) |
| Pneumonia with respiratory failure | 12 (28) | 16 (37) |
| Pneumonia with septic shock | 1 (2.3) | 0 (0.0) |
| Gastrointestinal hemorrhage | 3 (7.0) | 4 (9.3) |
| Pulmonary embolism | 1 (2.3) | 0 (0.0) |

Abbreviations: BUN, blood urea nitrogen; sCr, serum creatinine; sICH, symptomatic Intracranial Hemorrhage; AE, adverse event; SAE, severe adverse event. Circulation failure refers to hemodynamic instability resulting in shock and was used when the underlying etiology of shock could not be clearly determined or when multiple contributing factors were suspected.

**Table S9**. Details of Adverse Events Occurring Within the Safety Population

| No. | Treatment arm | Adverse events | Grade | Relationship to stroke | Relationship to minocycline | Relationship to EVT |
| --- | --- | --- | --- | --- | --- | --- |
| 1 | Minocycline group | Pneumonia | Mild | Definite | Unrelated | Unrelated |
| 2 | Minocycline group | Pneumonia | Mild | Definite | Unrelated | Unrelated |
| 3 | Minocycline group | Puncture site hematoma | Severe | Unlikely | Unrelated | Definite |
| 4 | Minocycline group | Liver enzyme elevation | Severe | Unlikely | Possible | Unrelated |
| 5 | Control group | Thrombocytopenia | Mild | Possible | Unrelated | Possible |
| 6 | Minocycline group | Anemia | Mild | Possible | Unrelated | Unrelated |
| 7 | Control group | Pneumonia | Mild | Definite | Unrelated | Unrelated |
| 8 | Minocycline group | Pneumonia | Severe | Definite | Unrelated | Unrelated |
| 9 | Minocycline group | Anemia | Mild | Possible | Unrelated | Unrelated |
| 10 | Minocycline group | Pneumonia | Severe | Definite | Unrelated | Unrelated |
| 11 | Control group | Pneumonia | Severe | Definite | Unrelated | Unrelated |
| 12 | Control group | Liver enzyme elevation | Severe | Unlikely | Unrelated | Unrelated |
| 13 | Control group | Pneumonia | Mild | Definite | Unrelated | Unrelated |
| 14 | Control group | Pneumonia | Mild | Definite | Unrelated | Unrelated |
| 15 | Control group | Respiratory failure | Severe | Definite | Unrelated | Unrelated |
| 16 | Minocycline group | Pneumonia | Severe | Definite | Unrelated | Unrelated |
| 17 | Control group | Pneumonia | Mild | Definite | Unrelated | Unrelated |
| 18 | Control group | Liver enzyme elevation | Severe | Unrelated | Unrelated | Unrelated |
| 19 | Minocycline group | Pneumonia | Mild | Definite | Unrelated | Unrelated |
| 20 | Minocycline group | Pneumonia | Severe | Definite | Unrelated | Unrelated |
| 21 | Minocycline group | BUN/sCr elevation | Mild | Unlikely | Possible | Unrelated |
| 22 | Minocycline group | Thrombocytopenia | Mild | Possible | Possible | Possible |
| 23 | Control group | Anemia | Mild | Possible | Unrelated | Unrelated |
| 24 | Control group | Pneumonia | Severe | Definite | Unrelated | Unrelated |
| 25 | Control group | Pneumonia | Severe | Definite | Unrelated | Unrelated |
| 26 | Control group | BUN/sCr elevation | Mild | Possible | Unrelated | Unrelated |
| 27 | Minocycline group | Pneumonia | Mild | Definite | Unrelated | Unrelated |
| 28 | Control group | Pneumonia | Mild | Definite | Unrelated | Unrelated |
| 29 | Control group | Pneumonia | Mild | Definite | Unrelated | Unrelated |
| 30 | Control group | Anemia | Mild | Possible | Unrelated | Unrelated |
| 31 | Control group | Liver enzyme elevation | Mild | Unlikely | Unrelated | Unrelated |
| 32 | Control group | Abnormal coagulation | Mild | Unlikely | Unrelated | Unrelated |
| 33 | Control group | Pneumonia | Severe | Definite | Unrelated | Unrelated |
| 34 | Control group | Anemia | Mild | Possible | Unrelated | Unrelated |
| 35 | Control group | Pneumonia | Mild | Definite | Unrelated | Unrelated |
| 36 | Minocycline group | Pneumonia | Mild | Definite | Unrelated | Unrelated |
| 37 | Minocycline group | Pneumonia | Severe | Definite | Unrelated | Unrelated |
| 38 | Minocycline group | Pneumonia | Severe | Definite | Unrelated | Unrelated |
| 39 | Control group | Pneumonia | Severe | Definite | Unrelated | Unrelated |
| 40 | Control group | Anemia | Mild | Possible | Unrelated | Unrelated |
| 41 | Control group | Liver enzyme elevation | Mild | Unlikely | Unrelated | Unrelated |
| 42 | Control group | Pneumonia | Severe | Definite | Unrelated | Unrelated |
| 43 | Control group | Liver enzyme elevation | Mild | Unlikely | Unrelated | Unrelated |
| 44 | Control group | BUN/sCr elevation | Mild | Possible | Unrelated | Unrelated |
| 45 | Minocycline group | Pneumonia | Mild | Definite | Unrelated | Unrelated |
| 46 | Control group | Pneumonia | Severe | Definite | Unrelated | Unrelated |
| 47 | Minocycline group | Pneumonia | Mild | Definite | Unrelated | Unrelated |
| 48 | Control group | Pneumonia | Mild | Definite | Unrelated | Unrelated |
| 49 | Minocycline group | Anemia | Mild | Possible | Unrelated | Unrelated |
| 50 | Minocycline group | Pneumonia | Severe | Definite | Unrelated | Unrelated |
| 51 | Minocycline group | BUN/sCr elevation | Mild | Possible | Possible | Unrelated |
| 52 | Control group | Pneumonia | Mild | Definite | Unrelated | Unrelated |
| 53 | Minocycline group | Pneumonia | Severe | Definite | Unrelated | Unrelated |
| 54 | Control group | Pneumonia | Mild | Definite | Unrelated | Unrelated |
| 55 | Control group | Pseudoaneurysm | Mild | Unrelated | Unrelated | Definite |
| 56 | Minocycline group | Pneumonia | Severe | Definite | Unrelated | Unrelated |
| 57 | Control group | Pneumonia | Mild | Definite | Unrelated | Unrelated |
| 58 | Control group | Pneumonia | Mild | Definite | Unrelated | Unrelated |
| 59 | Control group | Pneumonia | Severe | Definite | Unrelated | Unrelated |
| 60 | Control group | Abnormal coagulation | Mild | Unlikely | Unrelated | Unrelated |
| 61 | Control group | Liver enzyme elevation | Mild | Unlikely | Unrelated | Unrelated |
| 62 | Control group | BUN/sCr elevation | Mild | Possible | Unrelated | Unrelated |
| 63 | Control group | Pneumonia | Severe | Definite | Unrelated | Unrelated |
| 64 | Minocycline group | Pneumonia | Mild | Definite | Unrelated | Unrelated |
| 65 | Minocycline group | Pneumonia | Severe | Definite | Unrelated | Unrelated |
| 66 | Minocycline group | Pneumonia | Mild | Definite | Unrelated | Unrelated |
| 67 | Minocycline group | Gut infection | Mild | Definite | Unrelated | Unrelated |
| 68 | Control group | Puncture site hematoma | Severe | Unrelated | Unrelated | Definite |
| 69 | Control group | Pneumonia | Mild | Definite | Unrelated | Unrelated |
| 70 | Control group | Pneumonia | Mild | Definite | Unrelated | Unrelated |
| 71 | Control group | Liver enzyme elevation | Mild | Unlikely | Unrelated | Unrelated |
| 72 | Control group | Pneumonia | Mild | Definite | Unrelated | Unrelated |
| 73 | Control group | Abnormal coagulation | Mild | Unlikely | Unrelated | Unrelated |
| 74 | Minocycline group | Pneumonia | Mild | Definite | Unrelated | Unrelated |
| 75 | Control group | Pneumonia | Severe | Definite | Unrelated | Unrelated |
| 76 | Control group | Anemia | Mild | Possible | Unrelated | Unrelated |
| 77 | Minocycline group | Pneumonia | Severe | Definite | Unrelated | Unrelated |
| 78 | Minocycline group | Liver enzyme elevation | Mild | Unlikely | Possible | Unrelated |
| 79 | Control group | Pneumonia | Severe | Definite | Unrelated | Unrelated |
| 80 | Minocycline group | Pneumonia | Mild | Definite | Unrelated | Unrelated |
| 81 | Minocycline group | Pseudoaneurysm | Mild | Unrelated | Unrelated | Definite |
| 82 | Minocycline group | Pneumonia | Mild | Definite | Unrelated | Unrelated |
| 83 | Control group | Pneumonia | Mild | Definite | Unrelated | Unrelated |
| 84 | Control group | Pneumonia | Mild | Definite | Unrelated | Unrelated |
| 85 | Minocycline group | Pneumonia | Mild | Definite | Unrelated | Unrelated |
| 86 | Minocycline group | Pneumonia | Mild | Definite | Unrelated | Unrelated |

**Table S10.** Details of Severe Adverse Events Occurring Within the Safety Population

| No. | Treatment arm | Severe Adverse events | Grade | Relationship to stroke | Relationship to minocycline | Relationship to EVT |
| --- | --- | --- | --- | --- | --- | --- |
| 1 | Minocycline group | Pneumonia with septic shock | Severe | Definite | Unrelated | Unrelated |
| 2 | Minocycline group | Death | Severe | Definite | Unrelated | Unlikely |
| 3 | Control group | Pneumonia with respiratory failure | Severe | Definite | Unrelated | Unrelated |
| 4 | Control group | Symptomatic intracranial hemorrhage | Severe | Definite | Unrelated | Probable |
| 5 | Control group | Death | Severe | Definite | Unrelated | Unlikely |
| 6 | Minocycline group | Pneumonia with respiratory failure | Severe | Definite | Unrelated | Unrelated |
| 7 | Minocycline group | Symptomatic intracranial hemorrhage | Severe | Definite | Unlikely | Probable |
| 8 | Minocycline group | Death | Severe | Definite | Unrelated | Unlikely |
| 9 | Minocycline group | Pneumonia with respiratory failure | Severe | Definite | Unrelated | Unrelated |
| 10 | Minocycline group | Death | Severe | Definite | Unrelated | Unrelated |
| 11 | Minocycline group | Pneumonia with respiratory failure | Severe | Definite | Unrelated | Unrelated |
| 12 | Control group | Pneumonia with respiratory failure | Severe | Definite | Unrelated | Unrelated |
| 13 | Control group | Pneumonia with respiratory failure | Severe | Definite | Unrelated | Unrelated |
| 14 | Control group | Circulation failure | Severe | Definite | Unrelated | Unrelated |
| 15 | Control group | Death | Severe | Definite | Unrelated | Unlikely |
| 16 | Control group | Pneumonia with respiratory failure | Severe | Definite | Unrelated | Unrelated |
| 17 | Control group | Symptomatic intracranial hemorrhage | Severe | Definite | Unlikely | Probable |
| 18 | Control group | Gastrointestinal hemorrhage | Severe | Definite | Unrelated | Unrelated |
| 19 | Control group | Death | Severe | Definite | Unrelated | Unlikely |
| 20 | Control group | Pneumonia with respiratory failure | Severe | Definite | Unrelated | Unrelated |
| 21 | Minocycline group | Pneumonia with respiratory failure | Severe | Definite | Unrelated | Unrelated |
| 22 | Minocycline group | Symptomatic intracranial hemorrhage | Severe | Definite | Unlikely | Probable |
| 23 | Minocycline group | Death | Severe | Definite | Unlikely | Unlikely |
| 24 | Minocycline group | Pneumonia with respiratory failure | Severe | Definite | Unrelated | Unrelated |
| 25 | Minocycline group | Gastrointestinal hemorrhage | Severe | Definite | Unlikely | Unrelated |
| 26 | Minocycline group | Death | Severe | Definite | Unrelated | Unlikely |
| 27 | Control group | Pneumonia with respiratory failure | Severe | Definite | Unrelated | Unrelated |
| 28 | Control group | Death | Severe | Definite | Unrelated | Unlikely |
| 29 | Control group | Pneumonia with respiratory failure | Severe | Definite | Unrelated | Unrelated |
| 30 | Control group | Death | Severe | Definite | Unrelated | Unlikely |
| 31 | Control group | Pneumonia with respiratory failure | Severe | Definite | Unrelated | Unrelated |
| 32 | Control group | Gastrointestinal hemorrhage | Severe | Definite | Unrelated | Unrelated |
| 33 | Control group | Death | Severe | Definite | Unrelated | Unlikely |
| 34 | Minocycline group | Pneumonia with respiratory failure | Severe | Definite | Unrelated | Unrelated |
| 35 | Minocycline group | Circulation failure | Severe | Definite | Unrelated | Unrelated |
| 36 | Minocycline group | Pulmonary embolism | Severe | Definite | Unlikely | Unrelated |
| 37 | Minocycline group | Death | Severe | Definite | Unlikely | Unlikely |
| 38 | Minocycline group | Pneumonia with respiratory failure | Severe | Definite | Unrelated | Unrelated |
| 39 | Control group | Pneumonia with respiratory failure | Severe | Definite | Unrelated | Unrelated |
| 40 | Control group | Pneumonia with respiratory failure | Severe | Definite | Unrelated | Unrelated |
| 41 | Control group | Gastrointestinal hemorrhage | Severe | Definite | Unrelated | Unrelated |
| 42 | Control group | Death | Severe | Definite | Unrelated | Unlikely |
| 43 | Control group | Pneumonia with respiratory failure | Severe | Definite | Unrelated | Unrelated |
| 44 | Control group | Symptomatic intracranial hemorrhage | Severe | Definite | Unrelated | Probable |
| 45 | Control group | Death | Severe | Definite | Unrelated | Unlikely |
| 46 | Minocycline group | Pneumonia with respiratory failure | Severe | Definite | Unrelated | Unrelated |
| 47 | Minocycline group | Gastrointestinal hemorrhage | Severe | Definite | Unlikely | Unrelated |
| 48 | Minocycline group | Death | Severe | Definite | Unlikely | Unlikely |
| 49 | Minocycline group | Pneumonia with respiratory failure | Severe | Definite | Unrelated | Unrelated |
| 50 | Minocycline group | Death | Severe | Definite | Unlikely | Unlikely |
| 51 | Minocycline group | Pneumonia with respiratory failure | Severe | Definite | Unrelated | Unrelated |
| 52 | Control group | Symptomatic intracranial hemorrhage | Severe | Definite | Unrelated | Probable |
| 53 | Control group | Death | Severe | Definite | Unrelated | Unlikely |
| 54 | Control group | Gastrointestinal hemorrhage | Severe | Definite | Unrelated | Unrelated |
| 55 | Control group | Pneumonia with respiratory failure | Severe | Definite | Unrelated | Unrelated |
| 56 | Control group | Circulation failure | Severe | Definite | Unrelated | Unrelated |
| 57 | Control group | Death | Severe | Definite | Unrelated | Unlikely |
| 58 | Control group | Pneumonia with respiratory failure | Severe | Definite | Unrelated | Unrelated |
| 59 | Control group | Death | Severe | Definite | Unrelated | Unlikely |
| 60 | Control group | Symptomatic intracranial hemorrhage | Severe | Definite | Unrelated | Probable |
| 61 | Minocycline group | Pneumonia with respiratory failure | Severe | Definite | Unrelated | Unrelated |
| 62 | Minocycline group | Circulation failure | Severe | Definite | Unrelated | Unrelated |
| 63 | Minocycline group | Death | Severe | Definite | Unlikely | Unlikely |
| 64 | Control group | Ischemic heart disease | Severe | Definite | Unrelated | Unlikely |
| 65 | Control group | Death | Severe | Definite | Unrelated | Unlikely |
| 66 | Minocycline group | Symptomatic intracranial hemorrhage | Severe | Definite | Unlikely | Probable |
| 67 | Minocycline group | Gastrointestinal hemorrhage | Severe | Definite | Unlikely | Unrelated |
| 68 | Minocycline group | Death | Severe | Definite | Unlikely | Unlikely |
| 69 | Control group | Pneumonia with respiratory failure | Severe | Definite | Unrelated | Unrelated |
| 70 | Minocycline group | Pneumonia with respiratory failure | Severe | Definite | Unrelated | Unrelated |
| 71 | Control group | Pneumonia with respiratory failure | Severe | Definite | Unrelated | Unrelated |
| 72 | Control group | Death | Severe | Definite | Unrelated | Unlikely |
| 73 | Control group | Pneumonia with respiratory failure | Severe | Definite | Unrelated | Unrelated |
| 74 | Control group | Symptomatic intracranial hemorrhage | Severe | Definite | Unrelated | Probable |
| 75 | Control group | Death | Severe | Definite | Unrelated | Unlikely |

**Fig. S1** Enrollment Progress of MIST-B Trial over Trial Period

**Fig. S2** Distribution of mRS score at day 90 by treatment (modified Intention-to-Treat Population)

89 patients in the mITT analysis, including 45 patients in the minocycline group and 44 patients in the control group. Data of 1 patient in the minocycline group with missing data were not included. Treatment with adjunctive minocycline was associated with an adjusted win ratio of 1.34 (95%CI, 0.73-2.41) for the distribution of the mRS at 90 days. mITT, modified intention-to-treat; mRS, modified Rankin Scale.
